# Investigation of Dearomatizing Spirocyclizations and Spirocycle Functionalization En Route to Spirocalcaridines A and B—Some Trials and Tribulations

**DOI:** 10.3390/molecules30051143

**Published:** 2025-03-03

**Authors:** Ravi P. Singh, Delphine Gout, James X. Mao, Peter Kroll, Carl J. Lovely

**Affiliations:** 1 Department of Chemistry and Biochemistry, University of Texas at Arlington, Arlington, TX 76016, USAxjamesmao@tamu.edu (J.X.M.); pkroll@uta.edu (P.K.); lovely@chemistry.msstate.edu (C.J.L.); 2 High Performance Research Computing (HPRC), Texas A&M University, College Station, TX 77843, USA; 3 Department of Chemistry, Mississippi State University, Starkville, MS 39762, USA

**Keywords:** hypervalent iodine, dearomatizing spirocyclization, allylic hydroxylation, dearomatization

## Abstract

Spirocalcaridines A and B are among the most challenging members of the marine invertebrate-derived *Leucetta* alkaloids. Approaches to the construction and elaboration of the highly compact spirocyclic core are described. The synthesis of tricyclic guanidine via tandem oxidative amination dearomatizing spirocyclization (TOADS) using hypervalent iodine set the stage for total synthesis via the migration of the C4/C8 double bond to the C4/C5 position, followed by oxidation. The undesired but not surprising propensity of the spirocyclic cyclohexadienone to undergo rearrangement to the phenol hindered the desired olefin migration. Furthermore, initial efforts to install the oxidation sequentially, first at C5 and then at C4 in the complete carbon skeleton, were fraught with unforeseen challenges and unusual outcomes. In addition, the scope and limitations of hypervalent iodine-mediated tandem oxidative dearomatizing spirocyclization on various substrates were explored. Urethanes and thiourethanes underwent spirocyclization with an excellent yield, whereas the reaction with allylic substrates and species lacking the *p*-methoxy substituent did not proceed. Attempts to prepare other guanidine precursors are briefly discussed.

## 1. Introduction

Dearomatizing reactions are of substantial interest in modern synthetic organic chemistry, offering versatile and efficient approaches to the construction of complex, functionally rich molecular architectures [1] including natural products [2]. When conducted intramolecularly, this flexible methodology leads to the formation of spirocyclic compounds, where two rings share a common atom, serving as a pivotal step in the synthesis of various natural products [3], pharmaceuticals, and functional materials [4,5]. By breaking aromaticity, the resulting products possess unique reactivity profiles and three-dimensional structural motifs, facilitating access to diverse chemical space and enabling the creation of molecules with tailored properties. *Leucetta* alkaloids have received significant attention from medicinal chemists because of their potentially useful biological activities and have challenged the creativity and ability of the synthetic chemistry community to solve the synthetic challenges posed by the presence of unusual structural features that often-belying intrinsic challenges [6,7,8,9]. Spirocalcaridines A (**4**) and B (**5**) were described by the Crews lab in 2003 [10]. These two alkaloids, along with calcaridine A (**6**), were isolated from the Calcareous sponge *Leucetta* sp. These molecules exhibit unprecedented structural features for this family of marine sponges, including the first intrinsically chiral members of this group. For example, at the time, (+)-calcaridine A (**6**) was the only member of the family to contain a rearranged 4,4-dibenzyl-5-imidazol(on)e framework, although recently, additional examples of this skeleton have appeared in the literature [11,12]. On the other hand, spirocalcaridine A (**4**) has a unique structure featuring a hexahydrocyclopentamidazol-2-ylidenamine ring system spiro-fused to a cyclohexadienone ring. In addition to **4**, the corresponding OMe derivative (−)-spirocalcaridine B (**5**) was isolated from the same sample. Interestingly, spirocalcaridine B (**5**) does not seem to be an artifact from the isolation process, as both (**4**) and (**5**) were shown to be stable in methanol solutions for extended periods by Crews [10]. The constitutions of these molecules have been determined by NMR spectroscopy, but the relative stereochemistry of the three chiral centers are as yet unknown. The two compounds are deficient in appropriately positioned protons for use in NOE studies, precluding this assignment; in addition, the location of the methoxy group in spirocalcaridine B is considered tentative. The spirocalcaridines are not the only member of this family in which hydrogen deficiency has resulted in ambiguity regarding the precise structure. Spiroleucettadine was initially misassigned, and several unsuccessful synthetic efforts [13,14] to construct the proposed framework have raised questions regarding its constitution, ultimately resulting in revisions to the structure [15] and confirmation through total synthesis [2]. Given this stereochemical ambiguity for **4** (and **5**), there are four possible (relative) stereochemical arrangements (**8**–**11**) that can be suggested, but, based on ring strain considerations of the trans fusion of the two five membered rings, the two cis [3.3.0] diastereomers (**8** and **9**) are considered more likely. In fact, it is clear from DFT calculations at the wB97XD/def2tzvp level conducted on possible spirocalcaridine A stereoisomers that the cis-carbinolamines (**8** and **9**) are much more stable than trans ones. From observing optimized structures, it seems that they not only benefit from less strain but also may be stabilized due to a feasible intramolecular H-bond between the two –OH groups, which does not exist in the trans stereoisomers (**10** and **11**). Out of the two cis isomers, the structure with an endo *p*-methoxyphenyl group is lower in energy and is likely the more correct structure.

**Figure 1:**
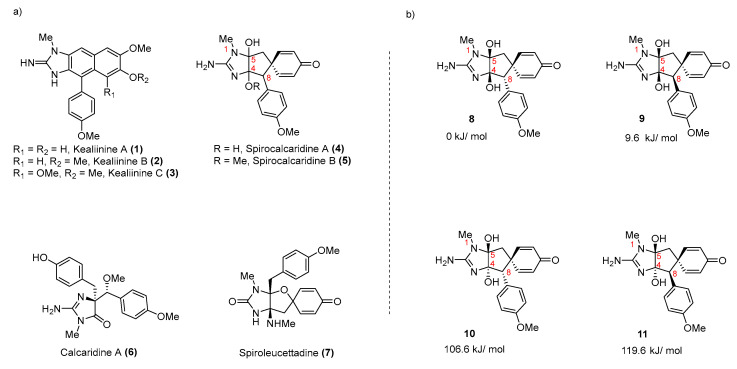
(**a**) A selection of the more highly oxidized *Leucetta* alkaloids. (**b**) Possible diastereomeric structures of spirocalcaridine A and their energies.

In general, imidazole containing natural products has been synthesized via two complementary approaches: first through the de novo construction of the imidazole ring [2,16] and second via functionalizing an existing imidazole ring [17,18]. In general, our group has pursued the latter approach, which has provided us with significant success in the total synthesis of several other family members [17,18,19]. However, functionalizing the existing imidazole ring to access the spirocalcaridines presented significant roadblocks, prompting us to investigate a de novo approach to imdazole construction. Indeed, in our exploratory approaches to the spirocalcaridines, we investigated dearomatization chemistries developed by Larock [20] and Li [21] to establish the spiro-fused 5,6 rings [22]. Furthermore, significant efforts were made to achieve de novo imidazole synthesis, leading to several highly functionalized intermediates suitable for total synthesis, along with some undesired yet constructive outcomes [19,22,23,24,25,26,27,28]. We have reported an unusual one-pot spirocyclization-N-cyanation reaction assisted by cyanogen bromide, leading directly to the spiro-fused derivative, a potential intermediate in synthetic approaches to several *Leucetta* alkaloids [29]. Hypervalent iodine-mediated dearomatizing spirocyclization represents a cutting-edge synthetic strategy that leverages the unique reactivity of hypervalent iodine reagents to facilitate the construction of spirocyclic compounds from aromatic precursors [30]. Moreover, the mild reaction conditions and broad substrate scope of hypervalent iodine-mediated reactions make it a versatile and environmentally benign strategy for accessing structurally and stereochemically diverse spirocycles, thus fueling innovation in synthetic methodology and molecular design. We have also reported the synthesis of tricyclic guanidine derivatives from propargyl guanidines via a hypervalent iodine-mediated tandem oxidative amination dearomatizing spirocyclization (TOADS) reaction, which set the stage for the total synthesis of spirocalcaridines [16], specifically through the migration of the C4/C8 double bond to the C4/C5 position followed by the oxidation and removal of protecting groups. Herein, we report on our efforts towards the exploration of these advanced intermediates for the completion of total synthesis and expand the scope and limitations of hypervalent iodine-mediated dearomatizing spirocyclization with this substrate (Figure 1).

## 2. Results and Discussions

**Scheme 1:**
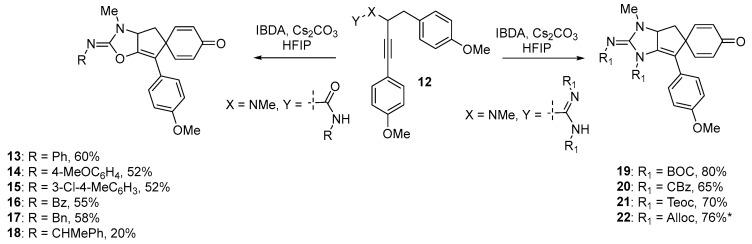
Previously reported dearomatizing spirocyclization of ureas and guanidines (* compound **22** has not been previously described).

As depicted in Scheme 1, our earlier report describes the hypervalent iodine-mediated oxidative dearomatizing spirocyclization of propargyl guanidines to construct the complete core present in spirocalcaridines A and B (**12**→**19**, **20**, and **21**) in addition to the previously unreported bis alloc analog (22*). Apart from the propargyl guanidines, propargylic ureas also participate in this chemistry upon treatment with iodosobenzene diacetate (IBDA), providing spiro-fused iminooxazoles (**12→13**, **14**, **15**, **16**, **17**, and **18**) [16].

**Scheme 2:**
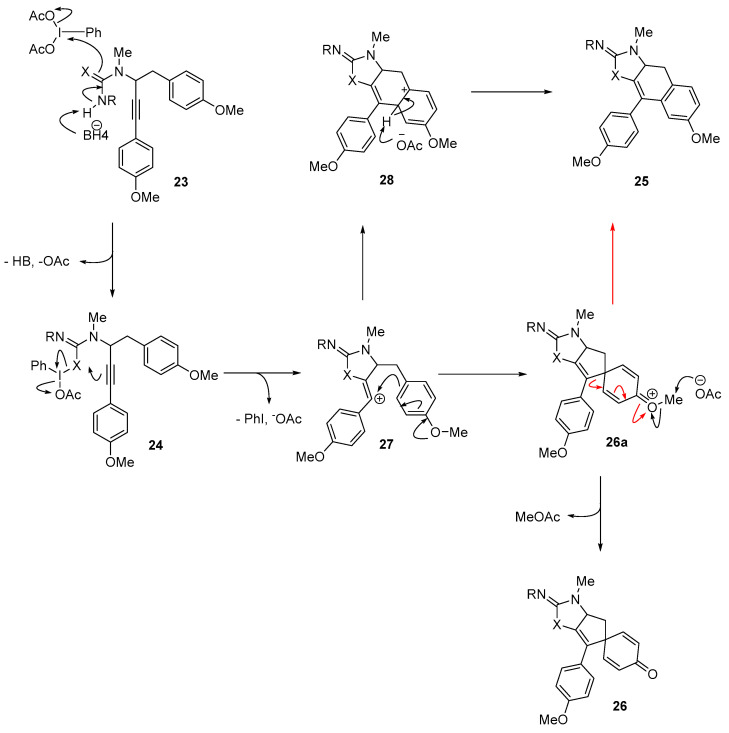
Putative mechanism behind the synthesis of spirocyclic guanidine and naphthoxazole in HFIP and TFE, respectively.

HFIP was found to be the only solvent to produce the spiro-fused iminooxazoles (**13** to **18**) and 2-iminoimidazoles (**19** to **22**). Interestingly, we observed the formation of naphthoxazole **25** (X = O, Scheme 2) upon using TFE as a solvent with the urea. A putative mechanism is given in Scheme 2; specifically, the urea oxygen or the guanidine nitrogen of **23** is activated by IBDA through ligand exchange, forming the electrophilic species **24**. Intramolecular addition to the electrophilic heteroatom then delivers the stabilized vinylic carbocation **27**, which then affords the spirocyclic product **26** upon the ipso addition of the electron-rich aromatic ring followed by demethylation, presumably by acetate ions. It is postulated that in TFE, due to its relatively lower dipolarity/polarizability (0.908) compared to HFIP (1.007) [31], the nucleophilicity of the acetate ion is impacted, which in turn results in the rearrangement of the spiro intermediate to the naphthalene derivative **28** prior to demethylation. Alternatively, the naphthalene framework **25** can be formed through the phenyl ring engaging directly with the vinyl carbocation to form a tertiary carbocation **28** which becomes aromatized to produce **25**. Other pathways, such as a concerted process or a radical-based pathway, cannot be ruled out at this time.

**Scheme 3:**
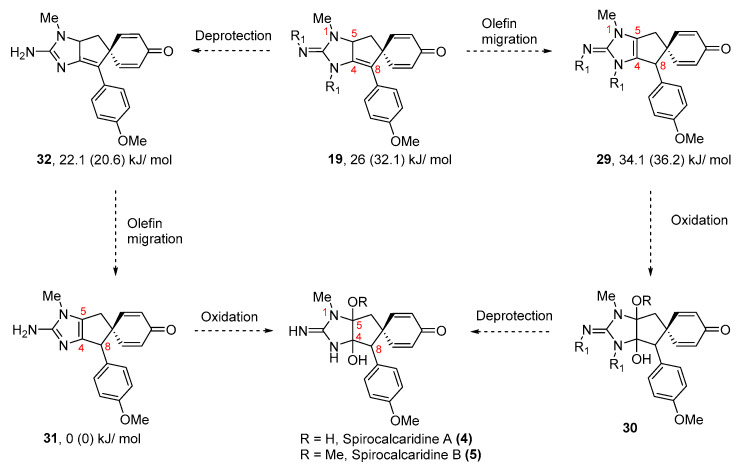
Proposed synthetic scheme to elaborate the spirocyclic intermediate to complete total synthesis and the energies of the TOADS products and corresponding rearranged products in the gas phase (after applying the solvation model MeOH).

These tricylic guanidines (**19** to **22**) contain the complete carbon skeleton of spirocalcaridine A and require the migration of the double bond from C4/C8 to the desired position C4/C5 (**19→29**, Scheme 3). Our expectation was that this migration would be driven by the aromatization of the imidazole. This type of process, in a general sense, has been observed in the Diels–Alder products from vinylimidazoles, where the initial adduct places a double bond exo-cyclic to the imidazole and this migrates either under thermal conditions (i.e., directly after the cycloaddition) or in the presence of electrophiles [32]. The precise repositioning of the olefin would allow us to perform oxidation across this double bond using chemistry developed in our group [33] previously to produce **30**, and, finally, the deprotection of the imidazole would complete total synthesis. Alternatively, it was also anticipated that the deprotection of the carbamates forming **32** followed by double bond migration would allow us to access **31** and, eventually, oxidation would conclude total synthesis.

**Scheme 4:**
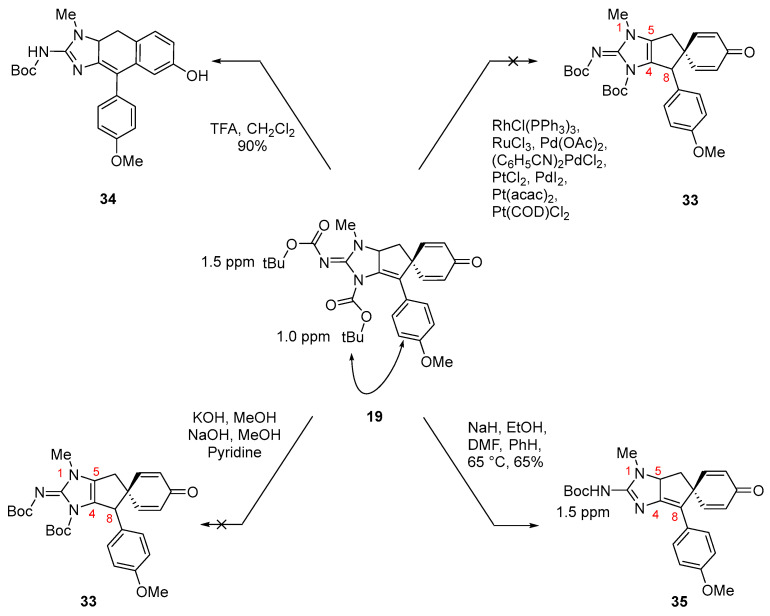
Efforts towards achieving double bond migration.

Numerous examples of double bond migrations promoted by transition metals [34,35], acids [36,37], and bases [38,39,40] are described in the literature. Since the bis-BOC substrate **19** is relatively easy to synthesize, attempts to affect the double bond migration on **19** using these conditions were made first. Several transition metal catalysts were tested [41,42], including RhCl(PPh_3_)_3_ [43], RuCl_3_ [44], Pd(OAc)_2_ [45], (C_6_H_5_CN)_2_PdCl_2_ [46], PtCl_2_ [47], PdI_2_ [42], Pt(acac)_2_ [47], and Pt(COD)Cl_2_ [47], to perform the double bond migration. In most cases, we observed complex mixtures including products formed via cyclohexadienone–phenol rearrangement (Scheme 4). Base-catalyzed reactions were also not effective for double bond migration; weaker bases such as DBU and pyridine were ineffective, whereas complex reaction mixtures were observed when stronger bases, e.g., NaOH and KOH, were used. In one attempt to perform this reaction using sodium ethoxide (made in situ by reacting NaH and EtOH) [40], we observed the cleavage of one BOC group. This outcome was not necessarily surprising per se, but what was unexpected was that the deprotected product was found to be significantly less polar on TLC. Based on this observation, it was hypothesized that the BOC group on the ring nitrogen (N3) is cleaved instead of the one on the 2-imino nitrogen. Since the double bond has moved inside the ring and is now in conjugation with the C4/C8 double bond, it is more stable. This hypothesis is further supported by ^1^H NMR data. The ^1^H NMR spectrum of **19** shows two distinct signals for the *t*-Bu groups. The one in the vicinity of the *p*-anisyl substituent has a signal at δ_H_ 1.0 ppm, whereas the other one on the exocyclic nitrogen appears at 1.5 ppm. After deprotection, the product with a mono BOC group shows the *t*-Bu signal at 1.5 ppm and the 1.0 ppm signal disappears, suggesting that the ring BOC has been cleaved. In addition, the removal of this BOC group allows greater rotational mobility in the *p*-anisyl group, and there is now more space for it to conjugate with the C4–C8 double bond. The upfield shift in the methyl signals of the t-Bu group is presumably a function of lying above the shielding cone of the *p*-anisyl group. This outcome is in accordance with our previously reported result, where a similar outcome was observed after treating the bis-BOC substrate with TFA. The process resulted in the cleavage of one of the BOC groups and cyclohexadienone–phenol rearrangement to produce **34**; the order of events is not known. The dihydronaphthimidazole intermediate **34** is crystalline, and the X-ray crystal structure clearly shows the connectivity of the BOC group as stated above. The remaining N-BOC group appears at δ_H_ 1.50 in the ^1^H NMR spectrum of **34** [16].

As we were unable to migrate the double bond, there was some concern regarding the viability of this step due to the increased strain of the double bond along the ring fusion, and thus DFT calculations were performed [48] to illuminate and possibly guide the next steps. The free energies of both **19** and **29** were calculated at the wB97XD [49]/def2tzvp [50,51] level. Interestingly, it was found that the rearranged product **29** was in fact 8.1 kJ/mole less stable than the precursor **19** in the gas phase. Calculations performed with solvation models [52] also showed that **19** was more stable, but the difference was attenuated quite substantially. In contrast, when the deprotected congener **32** and the corresponding rearranged congener **31** were evaluated, the relative stabilities switched. In this case, the incorporation of a solvation model resulted only in a modest change in relative stability. Whether this change is a function of the imidazole ring being fully aromatic in **31** compensating for lost stabilization due to the conjugation of the C4–C5 bond, whereas in **29** the imidazole is not aromatic, is unclear. However, this suggests that deprotection prior to rearrangement might be a constructive avenue for investigation.

**Scheme 5:**
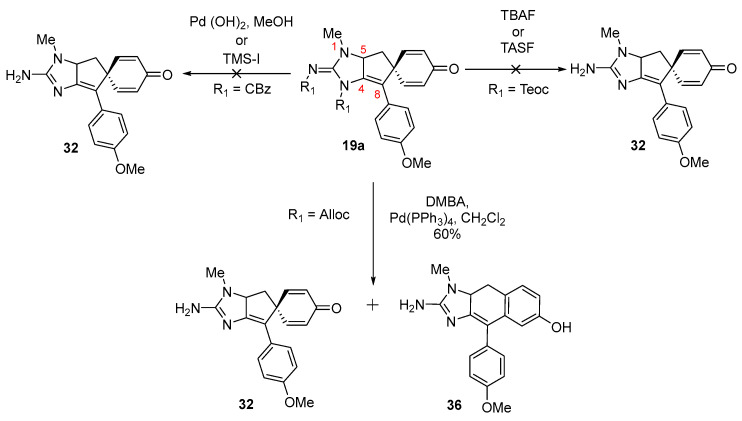
Efforts towards achieving the removal of protecting groups.

After unsuccessful attempts to achieve double bond migration and deprotection with the bis-BOC substrate, the focus shifted to the removal of protecting groups on the closely related analogs, specifically, the bis-Cbz, bis-Alloc, and bis-Teoc groups. The removal of bis-Cbz **20** was investigated using Pd(OH)_2_ and TMSI, but in both cases, the start of material decomposition was observed. A similar outcome was witnessed when the bis-Teoc intermediate **21** was treated with TBAF or TASF; the starting material was found to be unstable, and decomposition was observed. The deprotection of bis-Alloc derivative **22** using 1, 3-dimethyl barbituric acid (DMBA) and Pd(PPh_3_)_4_ resulted in the formation of a mixture of two products; one formed as a result of the usual and required deprotection process (**32**, Scheme 5), and the other was formed as a result of deprotection followed by cyclohexadienone–phenol rearrangement (**36**, Scheme 5). The structures were tentatively assigned based on the NMR data of the mixture, and these two products were found to be inseparable, possibly due to the continuous rearrangement of **32** to **36** during silica gel chromatography.

**Scheme 6:**
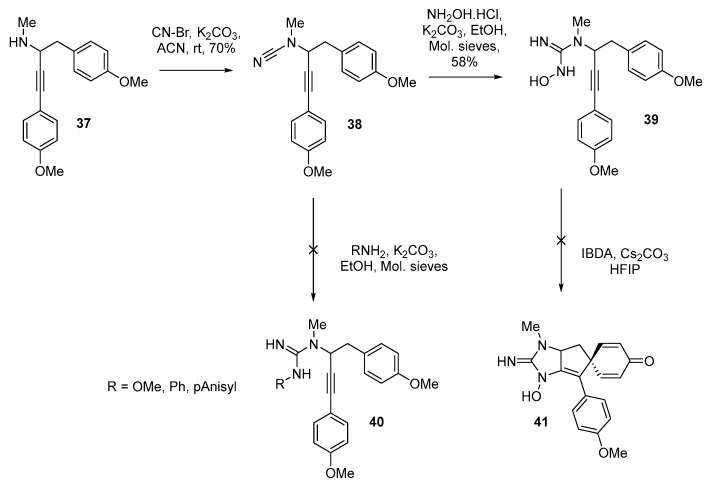
Efforts towards achieving the synthesis of unprotected guanidines.

As the efforts outlined in Scheme 5 and Scheme 6 depict, the removal of the nitrogen-protecting groups and the migration of the double bond provided a significant roadblock in the completion of total synthesis and thus forced us to explore alternative strategies. Accordingly, we explored substrates containing a propargylic guanidine precursor lacking N-protecting groups, avoiding deprotection at a later stage. The synthesis of these free guanidines was envisioned through the amination of corresponding cyanamide **38** (Scheme 6). Accordingly, as reported, the propargyl amine **37** was treated with CN-Br in the presence of K_2_CO_3_ to deliver the cyanamide via N-cyanation (Scheme 6) [29]. Cyanamide **38** was anticipated to be a potential substrate to access the corresponding guanidine, and an attempt was made to achieve guanylation followed by spirocyclization. Cyanamide **38** was converted to the corresponding N-hydroxyguanidine [53] **39** through treatment with hydroxylamine hydrochloride and K_2_CO_3_ in anhydrous EtOH. Other amines including anilines and methoxyamine were not effective for guanylation. N-hydroxyguanidine **39** was subjected to the TOADS conditions, but, unfortunately, a complex mixture formation was observed.

**Scheme 7:**
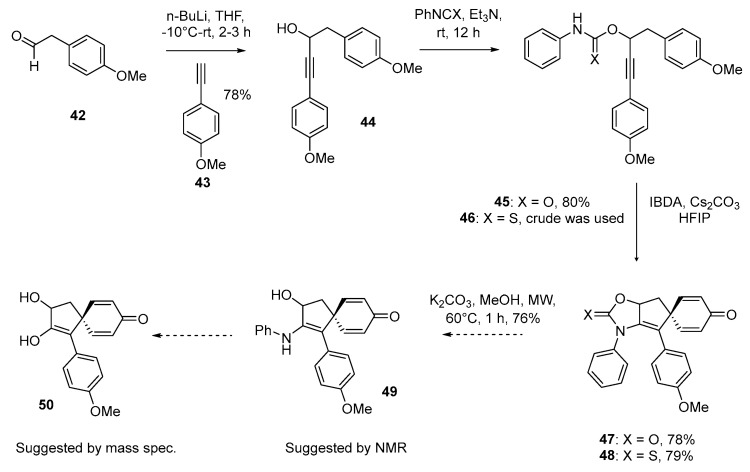
Dearomatizing spirocyclization of propargyl urethanes and thiourethanes.

An alternative strategy was considered, which involved the late-stage introduction of guanidine, post spirocyclization. This was the first investigation where the feasibility of the propargyl alcohol framework, rather than using the corresponding amine, was evaluated. Accordingly, acetaldehyde **42** was treated with lithium acetylide of 4-ethynylanisole **43** to construct the propargyl alcohol **44**, which was then converted to the corresponding phenyl urethane **45** and thiourethane **46** by reacting it with phenyl isocyanate and isothiocyanate, respectively. Both of these substrates were subjected to the previously established oxidative dearomatization conditions, and both substrates produced the spirocyclic compounds with good yields. Initially, it was uncertain whether cyclization took place via nitrogen or X (O/S), and the structures of the spirocyclic compounds were assigned tentatively as **47** and **48**. However, a hydrolysis experiment was performed on **47**, following the methods reported for the cleavage of oxazolidinones [54,55]. In particular, intermediate **47** was treated with K_2_CO_3_ in MeOH to produce a polar species. ^1^H and ^13^C NMR data showed that the cleavage was completed but the N-phenyl group was still intact, suggesting that **49** was the product. Interestingly, however, mass spectrometry data showed the mass of hydrolyzed product **50**. Since the conversion of enamines to enols is well known [56], it is conceivable that the hydrolysis had occurred under the mass spectrometry conditions. This outcome certainly confirms that cyclization is taking place through nitrogen to produce oxazolone **47**. While these spirocycles hold promise for applications in the total synthesis of spirocalcaridines, the discovery of the spirocyclization of urethane brings new opportunities in relation to the synthesis of spirocyclic α-hydroxy ketones. At this stage, guanidine substrates were revisited (Scheme 7).

**Scheme 8:**
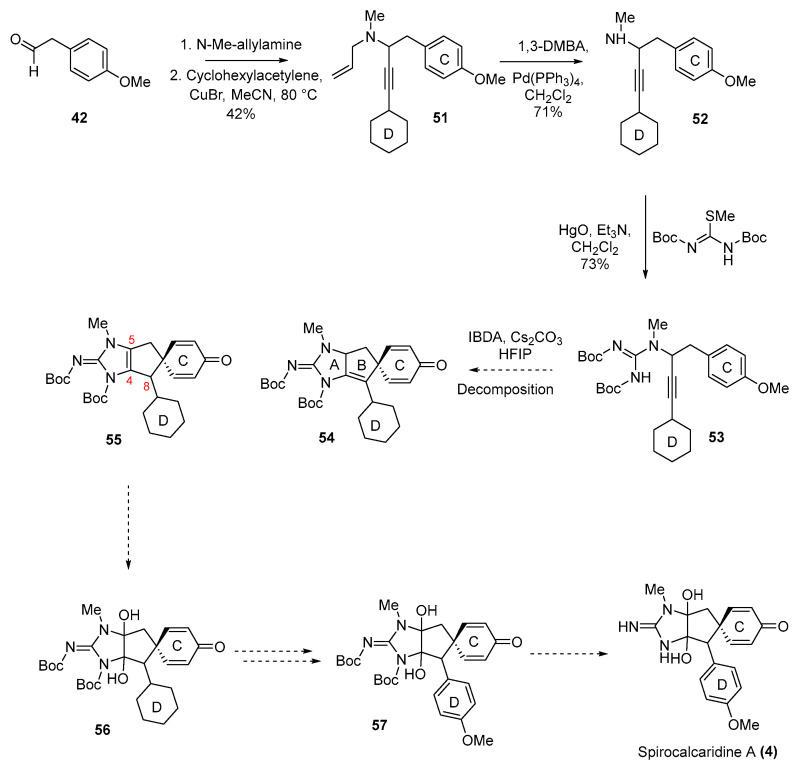
Scope of spirocyclization towards propargylic guanidines in the absence of stabilizing *p*-anisyl group—part 1.

As stated above, there are a number of ways to migrate the C4/C8 double bond inside the imidazole ring [32,57,58], and it is thought that the C4/C8 double bond migration to C4/C5 position has been recalcitrant because it is both tetrasubstituted and in conjugation with the *p*-methoxyphenyl D-ring [59,60], although, in the presence of N-protecting groups, it is unlikely to be fully coplanar due to steric crowding. Thus, the construction of a propargyl guanidine containing a cyclohexyl D-ring instead of *p*-anisyl D-ring was explored as a means of mitigating conjugation with C4/C8 double bond. The synthesis of propargyl guanidine **53** was identical to its aryl congener through a three-component coupling and was completed upon the treatment of the propargyl amine **52** with N,N′-di-BOC-S-methylisothiourea and mercuric oxide in the presence of Et_3_N in CH_2_Cl_2._ The platform was set for the construction of spirocyclic frameworks (**54** or **55**), and these intermediates would provide access to spirocalcaridines after oxidation state adjustments [61,62] and deprotection steps (Scheme 8), although it should be noted that the cyclohexyl group was just a model in the first instance. Thus, bis-BOC-guanidine **53** was subjected to the previously developed TOADS reaction conditions. Unfortunately, the reaction resulted in the decomposition of the substrate. This outcome is consistent with the intermediacy of vinylic carbocation **27** (Scheme 3) and its reduced stability in this case in the absence of the strongly electron-donating *p*-methoxyphenyl group [63]. Although the result of this particular campaign was disappointing, it offered some insights into the limitations of TOADS chemistry.

**Scheme 9:**
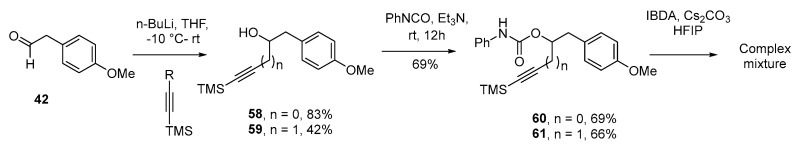
Scope of spirocyclization towards propargylic guanidines in the absence of stabilizing *p*-anisyl group—part 2.

Despite some limitations, the versatility of the TOADS reaction is quite remarkable, and we wanted to test the viability of a few other alkynes as nucleophiles. The synthesis of TMS propargyl urethanes appeared simpler than the corresponding guanidines, and the construction of propargyl urethane **60** and homopropargyl urethane **61** was undertaken simultaneously. In particular, aldehyde **42** was treated with lithium (trimethylsilyl)acetylide and (3-(trimethylsilyl)prop-2-yn-1-yl)lithium to afford the corresponding alcohols **58** and **59** [64], which were converted to the corresponding urethanes **60** and **61**, respectively, by reacting with phenylisocyanate and Et_3_N in CH_2_Cl_2_. The two TMS-protected urethanes **60** and **61** were subjected to the TOADS conditions. However, the conversion was not clean, and both substrates produced a complex mixture, providing additional insights that can be used in the limitation of the TOADS reaction (Scheme 9).

**Scheme 10:**
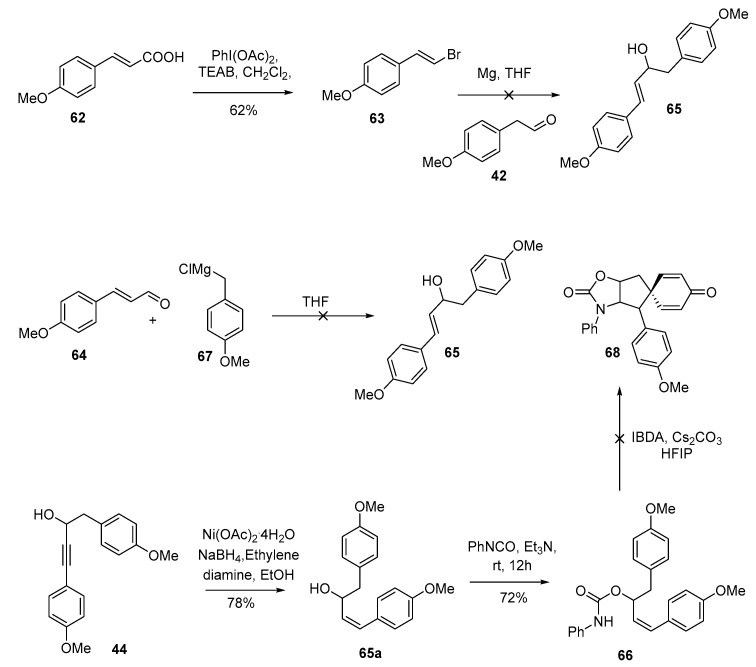
Scope of spirocyclization towards allylic urethanes.

Based on the outcome of the chemistries described in the synthetic schemes above, it seemed likely that a spirocyclic framework lacking both a C4/C8 and a C4/C5 double bond might serve more effectively as a pathway towards the total synthesis of spirocalcaridines A and B. In order to construct such a spirocyclic system, allylic substrate **66** was required, which would not only undergo the IBDA-assisted TOADS reaction to deliver spirocyclic framework **68** but also would provide insights regarding the general feasibility of TOADS chemistry. To the best of our knowledge, there were no prior reports on the synthesis of an analogous allylic guanidine. Again, as the construction of an allylic alcohol framework appeared easier in the first instance, our efforts were directed towards achieving its assembly. Accordingly, two parallel approaches were begun to construct allylic alcohol **65**. Having aldehyde **42** in hand, it was planned to react this with the Grignard of styryl bromide derivative **63** to deliver allylic alcohol **65**. Styryl bromide **63** was accessed via the IBDA-assisted decarboxylative bromination of 4-methoxycinnamic acid **62** [65]. However, all attempts to convert the styryl bromide to the corresponding Grignard were unsuccessful. Alternatively, the effect of changing the reaction sequence and reacting trans-*p*-methoxycinnamaldehyde with *p*-methoxybenzyl magnesium chloride was evaluated, again to no avail. Encouraged by a prior report on the efficient syn-reduction of an alkyne to an alkene [66,67], the previously synthesized propargyl alcohol **44** was reduced using NiBH_4_ (prepared in situ from Ni(OAc)_2_ and NaBH_4_) to afford the corresponding Z-alcohol **65a** in an excellent yield. The Z-allylic alcohol, thus obtained, was treated with phenylisocyanate to produce the corresponding urethane **66**. The subjection of the urethane **66** to the standard TOADS conditions did not afford the desired product, but a complex mixture was formed instead. It is important to mention here that after the addition of HFIP to urethane **66**, the color of the solution changed from colorless to light yellow. We wanted to investigate the HFIP solution of **66**; consequently, an aliquot was taken and was briefly examined. The HFIP-treated product was found to be relatively polar on TLC; the ^1^H NMR spectrum was also different. The complexity of the NMR data due to the presence of other products hindered us from reaching a conclusion, and once again, because of our focus on the completion of total synthesis, no attempts were made to further characterize the HFIP-treated product or to screen more oxidants in relation to the TOADS reaction of allylic urethanes (Scheme 10).

**Scheme 11:**
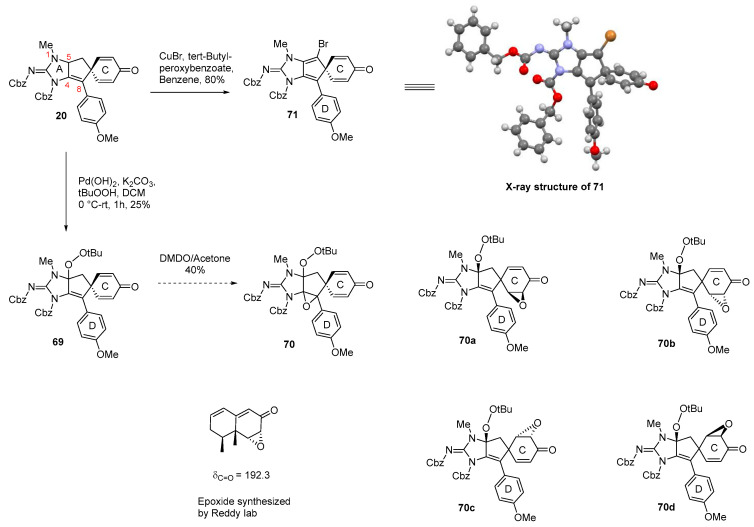
Exploration of oxidative strategies to complete total synthesis.

One of the advantages of synthetic chemistry is the ability to use different reagents or change the order of events to either circumvent roadblocks or to obtain different selectivities. Accordingly, we sought to investigate oxidative chemistries prior to deprotection and whether the C4–C8 double bond might engage in oxidative processes, thereby obviating the need for rearrangement. In a scouting experiment, the oxidation of the allylic position of bis-Cbz substrate **20** using conditions developed by Corey and Yu was tested. [68]. Although the yield was modest, the installation of the required functionality at C5 was accomplished and the peroxy intermediate **69** was obtained in a 25% yield, which was sufficient to carry out further scouting experiments. The initial plan was to carry out the epoxidation of the C4/C8 double bond of **69** and subject it to site-selective reductive ring opening. In reality, the peroxy intermediate **69** was treated with dimethyldioxirane (DMDO) in acetone for 2 days. The epoxidation was very slow, but this was somewhat expected because of the sterically hindered nature of the double bond. NMR data showed that a mixture of products was formed, which seemed reasonable due to anticipating *syn*/*anti* epoxidation with respect to the peroxy moiety. Several unsuccessful attempts were made to separate the products chromatographically; eventually, the mixture was subjected to conditions to achieve reductive epoxide ring opening [69]. The TLC profile of this epoxide ring-opening reaction was exciting as two new polar spots were formed with no starting material remaining. The two components were separated, and the NMR spectra were recorded of the two isolated components. It was clear from these data that the isolated products were in fact not single compounds. On closer examination of the NMR data of the initial epoxidation product and comparison with previously reported data [70], it became obvious that epoxidation had actually occurred on the cyclohexadienone ring (Scheme 11, **70a**–**70d**, δ_C=O_ = 192.3 (product) vs. 185.2 substrate) and not the cyclopentene. It was assumed that oxidation *anti* to the *p*-methoxybenzene group had occurred, giving rise to two possible constitutional isomers. Taking into consideration the possible future hurdles during epoxidation, no further attempt was made to take this approach forward.

One other reaction worth mentioning was uncovered during an attempt to improve the yield of the allylic hydroxylation reaction with the bis-Cbz analog **20**; it was observed that when stoichiometric CuBr was used with *tert*-butyl peroxybenzoate as an oxidant, a new oxidation product was formed [71]. In fact, the aim was to improve the yield of allylic oxidation, and the exploration of other metals was warranted, with copper serving as a viable alternative for palladium in oxidation [72]. Initially, catalytic CuBr was used in the presence of *tert*-butyl peroxybenzoate and benzene as a solvent. The reaction was sluggish and low-yielding at room temperature, while decomposition was observed at elevated temperatures. To circumvent this observation, a small excess of the reagent and catalyst were used. With 1 equiv. of catalyst and 5 equiv. of the oxidant, one major spot was observed on TLC, and starting material **20** was completely consumed. The yield of the isolated product was excellent, but, initially, determining the structure of this product was a challenge. However, it was clear from the ^1^H-NMR data that both the -CH_2_ and -CH protons on the B-ring were missing, and an assumption of ring desaturation was made. Mass spectrometry data indicated that bromine had been included in the product; based on the ^1^H NMR data, the most obvious location was on the B-ring. Fortunately, the material was crystalline, and upon obtaining an X-ray crystal structure, it was revealed that the product formed was in fact ring-oxidized product **71**. We did not perform any control reactions or further explore this chemistry, but the origin of the observed product was consistent with the net oxidation of the C5,C6 bond followed by bromine addition. It is assumed that this is via a radical pathway, but a polar mechanism cannot be fully ruled out in the absence of additional data.

## 3. Conclusions

In summary, we have evaluated several approaches to advancing the multifunctional spirocyclic core of spirocalcaridines A and B obtained via TOADS chemistries towards the completion of total synthesis. Significant roadblocks have emerged in the elaboration of the spirocyclic framework to permit the completion of total synthesis. The migration of the double bond from C4/C8 to C4/C5 position has been very challenging, and DFT calculations suggest that this may in fact be fatal to the approach based on an increase in strain. In fact, DFT calculations show that theenergy difference between the reactant and the product (**19** and **33**, R1 = BOC, Scheme 4) of the isomerization is about 8.1 kJ/mol with the reactant being more stable. The oxidation of C5 was shown to be partially successful, and a new oxidative bromination reaction was discovered using CuBr and *tert*-butyl peroxybenzoate. There are continuing efforts to utilize these versatile intermediates towards the completion of total synthesis, and the results will be reported in due course.

## Data Availability

The original contributions presented in this study are included in the article/Supplementary Material. Any reasonable request for original data supporting the work reported in this manuscript will be honored by the corresponding author.

## Supplementary Materials

The following supporting information can be downloaded at: https://www.mdpi.com/article/10.3390/molecules30051143/s1.
