# Supplementary material for: Investigation of Dearomatizing Spirocyclizations and Spirocycle Functionalization En Route to Spirocalcaridines A and B—Some Trials and Tribulations
Source: Molecules. 2025 Mar 3;30(5):1143. doi: 10.3390/molecules30051143 (PMC11902021; doi:10.3390/molecules30051143)

Supporting Information for

Investigation of Dearomatizing Spirocyclizations and Spirocycle Functionalization En Route to Spirocalcaridines A and B—Some Trials and Tribulations

Ravi P. Singh [1,*,†], Delphine Gout [1], James X. Mao [1,2], Peter Kroll [1] and Carl J. Lovely [1,3]

[1] Department of Chemistry and Biochemistry, University of Texas at Arlington, Arlington, TX 76016, USA; xjamesmao@tamu.edu (J.X.M.); pkroll@uta.edu (P.K.); lovely@chemistry.msstate.edu (C.J.L.)
[2] High Performance Research Computing (HPRC), Texas A&M University, College Station, TX 77843, USA
[3] Department of Chemistry, Mississippi State University, Starkville, MS 39762, USA
* Correspondence: rsingh@sutrobio.com
† Current Address: Sutro Biopharma, 111 Oyster Point Blvd, South San Francisco, CA 94080, USA.

Experimental procedures and characterization data for compounds **12, 22, 35, 38, 39, 44, 45, 47, 48-53, 58-61, 65, 66, 69-71.**

Copies of **^{1}H NMR** and **^{13}C NMR** data for compounds **12, 22, 35, 38, 39, 44, 45, 47, 48-53, 58-61, 65, 66, 69-71.**

General Information:

All reagents were purchased from commercial suppliers and were used as received unless otherwise stated. Reactions were performed in oven-dried glassware (24 h at 120 °C) under an atmosphere of dry nitrogen using solvents that had been dried and purified using alumina columns. Analytical thin layer chromatography (TLC) was performed on silica gel 60F$_{254}$ aluminum precoated plates (0.25 mm layer) and were visualized using UV light at 254 nm. All chromatographic purifications were performed using the flash chromatography method on silica gel (230-400 mesh). ^{1}H and ^{13}C NMR (δ in ppm) spectra were recorded in $CDCl_3$ (unless otherwise noted) at 500 and 125.8 MHz, respectively (unless otherwise noted). In some cases (where noted) ^{1}H and ^{13}C NMR spectra were recorded at 300 and 75 MHz respectively. Residual $CHCl_3$ (δ = 7.26) as reference for ^{1}H NMR and carbon absorption of $CDCl_3$ (δ = 77.0) as internal reference for ^{13}C NMR were used. Data are reported as s = singlet, d = doublet, t = triplet, q = quartet, dd = doublet of doublets, dt = doublet of triplets, td = triplet of doublets, tt = triplet of triplets, m = multiplet. Infrared spectra were recorded neat using an ATR instrument. High resolution mass spectra (HR-MS) were acquired in the Shimadzu Center for Advanced Analytical Chemistry using electrospray ionization with mass being measured using TOF.

1-(1-(4-Methoxyphenyl)-4-(4-methoxyphenyl)but-3-yn-2-yl)-1,3-bis(allyloxycarbonyl)-2-methylguanidine (12):

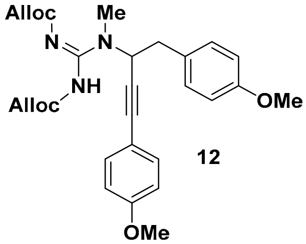

12

In a round bottom flask was added the 1, 3 bis (allyloxy carbonyl) 2-methyl-2-pseudothiourea (0.175 g, 0.67 mmol), HgO (0.147 g, 0.67 mmol mmol), Et_3N (0.094 mL, 0.67 mmol) and anhydrous CH2Cl2 (5 mL). The reaction mixture was allowed to stir for 10 min. A solution of *N*-methyl propargylamine (0.2 g, 0.67 mmol) in anhydrous CH2Cl2 (1.5 mL) was added and reaction mixture was allowed to stir for additional 16 h. The reaction mixture was filtered through celite, and solvent was removed under vacuum. The obtained crude was purified by column chromatography over silica gel (solid load, 0-15% EtOAc/ Hexanes as eluent) to afford **12**.

Physical appearance: Colorless gum.

Yield: 0.2 g, 58%.

^{1}H NMR: δ 7.35 – 7.28 (m, 2H), 7.26 (d, *J* = 8.3 Hz, 2H), 6.89 – 6.76 (m, 4H), 6.17 – 5.74 (m, 3H), 5.34 (dq, *J* = 17.2, 1.6 Hz, 2H), 5.24 (d, *J* = 11.0 Hz, 2H), 4.66 – 4.57 (m, 4H), 3.82 – 3.74 (m, 6H), 3.20 – 2.99 (m, 5H).

^{13}C NMR: δ 163.4, 162.7, 159.8, 158.7, 155.8, 133.3, 132.2, 131.9, 130.8, 128.7, 118.7, 118.2, 114.6, 114.0, 113.8, 86.5, 84.2, 67.4, 66.8, 55.4, 55.3, 52.7, 39.2, 33.7.

FT-IR (neat, cm^{-1}): 2935, 2836, 2227, 1750, 1635, 1602.

HR-MS (*m/z*): calc for $[M+Na]^+$ $C_{28}H_{32}N_3O_6$, 528.2105; found 528.2102.

Allyl (Z)-2'-(((allyloxy)carbonyl)imino)-6'-(4-methoxyphenyl)-3'-methyl-4-oxo-2',3',3a',4'-tetrahydro-1'H-spiro[cyclohexane-1,5'-cyclopenta[*d*]imidazole]-2,5-diene-1'-carboxylate (22):

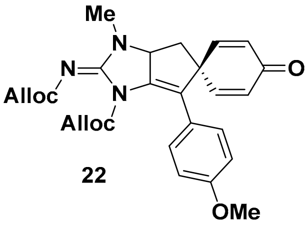

22

To a stirred solution of propargyl guanidine, **12** (0.2 g, 0.4 mmol) in HFIP (6 mL) at rt was added Cs_2CO_3 (0.195 g, 0.6 mmol). After 30 minutes IBDA (0.155 g, 0.48 mmol) was added to the reaction mixture (color changes from light yellow to light brown). The resulting reaction mixture was stirred at same temperature for 1 h. The reaction mixture was poured into an

ice-cooled saturated solution of sodium chloride (25 mL) and extracted with CH_2Cl_2 (2 x 50 mL). The combined organic extracts were dried with sodium sulfate and concentrated under reduced pressure. The crude was purified by column chromatography over silica gel (solid load, 0-80% EtOAc/ Hexanes as eluent) to afford allyl (Z)-2'-(((allyloxy)carbonyl)imino)-6'-(4-methoxyphenyl)-3'-methyl-4-oxo-2',3',3a',4'-tetrahydro-1'H-spiro[cyclohexane-1,5'-cyclopenta[*d*]imidazole]-2,5-diene-1'-carboxylate, **22**.

^{1}H NMR: δ 7.23 (dd, *J* = 10.0, 2.9 Hz, 1H), 7.07 – 6.99 (m, 2H), 6.78 – 6.69 (m, 2H), 6.59 (dd, *J* = 10.0, 3.0 Hz, 1H), 6.47 – 6.38 (m, 1H), 6.26 – 6.17 (m, 1H), 6.02 (ddt, *J* = 16.6, 10.3, 5.9 Hz, 1H), 5.37 (dq, *J* = 16.3, 1.6 Hz, 1H), 5.27 – 5.19 (m, 2H), 5.00 – 4.93 (m, 2H), 4.78 (dd, *J* = 8.1, 6.7 Hz, 1H), 4.64 (qdt, *J* = 13.1, 5.9, 1.6 Hz, 2H), 4.35 (ddt, *J* = 12.7, 6.3, 1.3 Hz, 1H), 4.21 (ddt, *J* = 12.7, 6.3, 1.3 Hz, 1H), 3.75 (s, 3H), 2.97 (s, 3H), 2.41 – 2.33 (m, 2H). **^{13}C NMR**: δ 185.4, 159.7, 159.4, 152.4, 152.3, 151.2, 148.8, 134.5, 133.1, 130.5, 129.8, 129.6, 128.5, 127.9, 125.3, 119.5, 118.0, 113.6, 68.1, 67.0, 64.2, 57.9, 55.3, 45.5, 30.2.

FT-IR (neat, cm^{-1}): 2934, 2838, 1752, 1659, 1614, 1510, 1435, 1368, 1278, 1198.

HR-MS (m/z): calc for $[M+H]^+$ $C_{27}H_{28}N_3O_6$, 490.1973; found: 490.1971.

Physical appearance: Colorless gum.

Yield: 0.147 g, 76%.

tert-Butyl (4'-(4-methoxyphenyl)-1'-methyl-4-oxo-6',6a'-dihydro-1'H-spiro[cyclohexane-1,5'-cyclopenta[d]imidazole]-2,5-dien-2'-yl)carbamate (35):

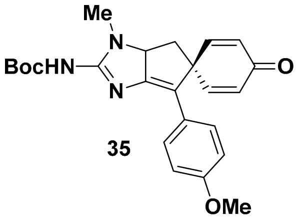

To a stirred solution of bis Boc 2-aminoimidazole **58** (0.025 g, 0.047 mmol) in DMF: toluene: EtOH (0.21 mL, 1:1:0.1) at 0 °C was added NaH (0.002 mg, 0.047 mmol, 60% suspension in mineral oil). The reaction mixture was stirred at 65 °C for 1.0 h and then was poured into ice-cooled water (1 mL) and extracted with CH_2Cl_2 (2 x 5 mL). The organic extracts were

dried with sodium sulfate and concentrated under reduced pressure. The crude product was purified by column chromatography over silica gel (solid load, 0-40% EtOAc/ Hexanes as eluent) to afford the desired product **35**.

Physical appearance: Colorless gum.

Yield: 0.013 g, 65%.

^{1}H NMR: δ 10.10 (s, 1H), 7.15 (dd, *J* = 10.0, 2.9 Hz, 1H), 7.06 (d, *J* = 9.1 Hz, 2H), 6.81 (t, *J* = 9.1 Hz, 3H), 6.37 (dd, *J* = 9.9, 1.9 Hz, 1H), 6.30 (dd, *J* = 9.9, 1.9 Hz, 1H), 4.64 (t, *J* = 7.8 Hz, 1H), 3.75 (s, 3H), 2.96 (s, 3H), 2.31 (d, *J* = 7.8 Hz, 2H), 1.50 (s, 9H).

^{13}C NMR: δ 185.6, 164.8, 163.2, 158.8, 153.6, 151.6, 139.5, 129.6, 129.5, 127.5, 125.5, 114.5, 112.4, 79.7, 65.8, 58.3, 55.4, 45.2, 30.9, 28.3.

FT-IR (neat, cm^{-1}): 3326, 3304, 2974, 2930, 2840, 1660, 1602, 1435, 1247, 1147.

HR-MS (*m/z*): calc for $[M+H]^+$ $C_{24}H_{28}N_3O_4$, 422.2074; found: 422.2078.

1-(4-(Methoxy)phenyl)-4-(4-methoxyphenyl)-*N*-methylbut-3-yn-2-cyanamide (38):

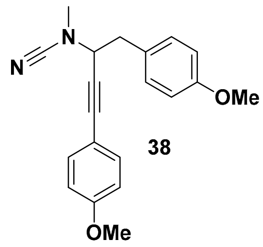

38

In a round bottom flask was added 1-(4-(methoxy)phenyl)-4-(4-methoxyphenyl)-*N*-methylbut-3-yn-2-amine **37** (2.5 g, 8.5 mmol) in acetonitrile (85 mL) followed by the addition of K_2CO_3 (2.3 g, 16.9 mmol). The reaction mixture was stirred at rt for 30 min. To this was added cyanogen bromide (3.2 g, 29.7 mmol) and the reaction mixture was stirred at rt for 16 h. The reaction mixture was diluted with cold water (100 mL), extracted with EtOAc (2 x 100 mL). The organic extracts were combined and washed with water (50 mL) followed by brine (50 mL), dried over sodium sulfate and concentrated under reduced pressure. The obtained crude was purified by column chromatography over silica gel (solid load, 0-10% EtOAc/ Hexanes as eluent) to afford compound **38**.

Physical appearance: Light yellow solid.

m.p. = 64-68 °C.

Yield: 1.9 g, 70%.

^{1}H NMR: 7.34 (d, *J* = 8.6 Hz, 2H), 7.21 (d, *J* = 8.6 Hz, 2H), 6.84 (d, *J* = 8.6 Hz, 2H), 6.80 (d, *J* = 8.6 Hz, 2H), 4.01 (t, *J* = 7.4 Hz, 1H), 3.74 (s, 3H), 3.74 (s, 3H), 3.08-3.05 (m, 2H), 2.85 (s, 3H).

^{13}C NMR: δ = 160.1, 158.8, 133.4, 130.5, 128.2, 116.9, 114.1, 114.0, 113.9, 87.4, 83.0, 56.7, 55.4, 55.3, 39.4, 37.6.

FT-IR (neat, cm^{-1}): 3004, 2961, 2908, 2836, 2209, 1732, 1688, 1605, 1584, 1318, 1241, 1174, 1052, 824, 803, 759, 557, 530.

HR-MS (*m/z*): calc for $[M+Na]^+$ $C_{20}H_{20}N_2O_2Na$, 343.1417; found: 343.1419.

1-(1,4-bis(4-Methoxyphenyl)but-3-yn-2-yl)-3-hydroxy-1-methylguanidine (39):

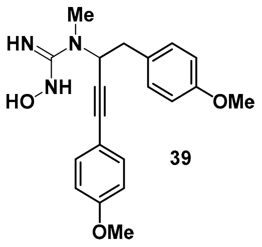

39

To a stirred suspension of **38** (0.25 g, 0.781 mmol) and molecular sieves (4 Å, 8-12 mesh) in EtOH (8 mL) was added K_2CO_3 (0.86 g, 6.25 mmol) followed by the addition of hydroxylamine hydrochloride (0.217 g, 3.12 mmol) and the reaction mixture was stirred at rt for 12 h. The reaction mixture was filtered through Celite and the filter cake was washed with 10% MeOH/CH_2Cl_2 (2 x 25 mL). The combined filtrates were concentrated under reduced pressure and the crude residue thus obtained was purified by column chromatography over silica gel (solid load, 0-10% MeOH/ CH_2Cl_2 as eluent) to afford product **39**.

Physical appearance: Brown solid.

m.p. = 110-112 °C.

Yield: 0.16 g, 58%.

^{1}H NMR: δ 7.36 – 7.29 (m, 2H), 7.19 (dd, *J* = 9.8, 7.7 Hz, 2H), 6.86 – 6.77 (m, 4H), 4.76 (t, *J* = 7.4 Hz, 1H), 4.22 (s, 1H), 3.80 – 3.73 (m, 6H), 3.00 – 2.92 (m, 2H), 2.82 (d, *J* = 18.3 Hz, 3H).

^{13}C NMR: δ 159.6, 158.50, 156.3, 133.2, 130.6, 130.1, 115.0, 114.0, 113.9, 85.7, 85.7, 55.4, 55.3, 53.7, 39.0, 31.3.

FT-IR (neat, cm^{-1}): 3515, 3322, 3182, 2100, 1510.

HR-MS (*m/z*): calcd. for $[M + H]^+C_{20}H_{24}N_3O_3$, 354.4229; found 354.4221.

1,4-bis(4-Methoxyphenyl)but-3-yn-2-ol (44):

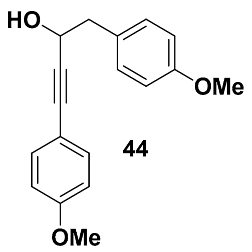

44

To a stirred solution of anisole **43** (1.0 g, 7.56 mmol) in anhydrous THF (20 mL) at -78 °C was added *n*-BuLi (5 mL, 7.5 mmol, 1.5 M in hexanes) dropwise. The resulting light-yellow solution was allowed to warm to 0 °C and stirred for 30 minutes. The solution was re-cooled to -78 °C and to this was added dropwise, a solution of aldehyde **42** (0.57 g, 3.78 mmol, dissolved in 5 mL THF). The reaction mixture was allowed to warm to rt and stirred for 30 minutes. The mixture was diluted with saturated aqueous ammonium chloride solution (10 mL) and EtOAc (50 mL). The two layers were separated and the aqueous solution was back extracted with EtOAc (2 x 15 mL). The combined organic extracts were washed with brine (10 mL), dried over sodium sulfate and concentrated under reduced pressure. The crude product was purified by column chromatography over silica gel (solid load, 0-25% EtOAc/ Hexanes as eluent) to afford the desired product **44**.

Physical appearance: Light yellow solid.

m.p. = 66-68 °C.

Yield: 0.835 g, 78%

^{1}H NMR: δ 7.36 – 7.31 (m, 2H), 7.25 (d, *J* = 8.7 Hz, 2H), 6.90 – 6.85 (m, 2H), 6.85 – 6.80 (m, 2H), 4.74 (q, *J* = 6.1 Hz, 1H), 3.80 (s, 3H), 3.79 (s, 3H), 3.03 (t, *J* = 5.9 Hz, 2H), 2.13 (d, *J* = 5.9 Hz, 1H).

^{13}C NMR: δ 159.8, 158.7, 133.2, 131.0, 128.8, 114.7, 114.0, 113.9, 88.3, 85.8, 63.9, 55.4, 55.4, 43.4.

FT-IR (neat, cm^{-1}): 3538, 2958, 2915, 2838, 2221, 1601, 1507, 1242, 1171.

HR-MS (*m/z*): calcd. for $[M + Na]^+ C_{18}H_{18}O_3Na$, 305.1148; found 305.1146.

1,4-bis(4-Methoxyphenyl)but-3-yn-2-yl phenylcarbamate (45):

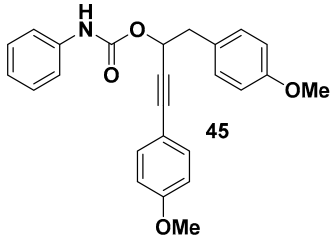

45

To a stirred solution propargyl alcohol **44** (0.05 g, 0.17 mmol) in CH_2Cl_2 (2.0 mL) at rt was added Et_3N (0.03 mL, 0.21 mmol) followed by the addition of phenylisocyanate (0.023 g, 0.19 mmol). The resulting light-yellow solution was stirred for 16 h. The reaction mixture was diluted with water (2 mL) and CH_2Cl_2 (5 mL). The two layers were separated, and the aqueous phase was back extracted with CH_2Cl_2 (2 x 5 mL). The combined organic solutions were washed with brine (2.5 mL), dried over sodium sulfate and concentrated under reduced pressure. The crude thus obtained was purified by column chromatography over silica gel (solid load, 0-25% EtOAc/ Hexanes as eluent) to afford the desired product **45**.

Physical appearance: Light yellow gum.

Yield: 0.057 g, 80%

^{1}H NMR: δ 7.41 – 7.29 (m, 3H), 7.32 – 7.24 (m, 5H), 7.06 (tt, *J* = 7.4, 1.2 Hz, 1H), 6.89 – 6.78 (m, 4H), 5.80 (t, *J* = 6.5 Hz, 1H), 3.79 (s, 3H), 3.78 (s, 3H), 3.18 – 3.12 (m, 2H).

^{13}C NMR: δ 160.0, 158.7, 152.5, 137.8, 133.5, 131.0, 129.2, 128.2, 123.7, 118.8, 114.4, 114.0, 113.9, 86.7, 84.9, 66.5, 55.4, 55.3, 40.8.

FT-IR (neat, cm^{-1}): 3320, 3037, 2999, 2933, 2835, 2224, 1707, 1602, 1508, 1212.

HR-MS (*m/z*): calcd. for $[M + Na]^+ C_{25}H_{24}NO_4Na$, 424.1519; found 424.1517.

4'-(4-Methoxyphenyl)-3'-phenyl-6',6a'-dihydrospiro[cyclohexane-1,5'-cyclopenta[d]oxazole]-2,5-diene-2',4(3'H)-dione (47):

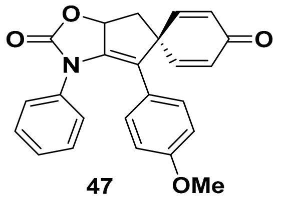

47

To a stirred solution propargyl urethane **45** (0.025 g, 0.06 mmol) in HFIP (1.8 mL) at rt was added Cs_2CO_3 (0.024 g, 0.074 mmol). After stirring the reaction mixture for 10 minutes, IBDA (0.03 g, 0.093 mmol) was added and the reaction mixture was stirred for 1 h. The reaction was diluted with water (2 mL) and CH_2Cl_2 (5 mL). The two layers were separated, and aqueous phase was back extracted with CH_2Cl_2 (2 x 5 mL). The combined organic extracts were washed with brine (2.5 mL), dried over sodium sulfate and concentrated under reduced pressure. The crude material thus obtained was purified by column chromatography over silica gel (solid load, 0-50% EtOAc/ Hexanes as eluent) to afford the desired product **47**.

Physical appearance: Colorless gum.

Yield: 0.019 g, 78%

^{1}H NMR: δ 7.23 (dd, *J* = 10.0, 3.0 Hz, 1H), 7.19 – 7.16 (m, 3H), 7.16 – 7.09 (m, 2H), 6.64 (dd, *J* = 10.0, 2.9 Hz, 1H), 6.55 – 6.50 (m, 2H), 6.45 (dd, *J* = 10.0, 2.0 Hz, 1H), 6.44 – 6.39 (m, 2H), 6.22 (dd, *J* = 10.0, 1.9 Hz, 1H), 5.68 (dd, *J* = 9.2, 6.1 Hz, 1H), 3.65 (s, 3H), 2.67 – 2.51 (m, 2H).

Note: While acquiring the ^{13}C NMR spectrum for 12 h, cyclohexadienone-phenol rearrangement (**47→47a**) was observed in deuterated chloroform (presumably because of the presence of residual DCl).

^{13}C NMR (contaminated with **47a** during cyclohexadienone-phenol rearrangement, peaks which correspond to the rearranged product **47a** are *underlined*): δ 185.4, 159.1, *158.6*, *157.5*, 156.7, *155.3*, 152.40, 151.34, *138.9*, 138.0, *135.7*, *134.5*, 133.8, *131.5*, 130.1, *129.7*, 129.52, 128.9, 128.8, *128.5*, *127.8*, *127.7*, *126.7*, *125.6*, 123.5, 123.2, *121.2*, 116.3, *113.4*, 113.1, *113.0*, *112.5*, *110.1*, 81.9, *76.3*, 57.8, *55.4*, 55.2, 51.0, 45.2, *33.5*, 29.8.

FT-IR (neat, cm^{-1}): 2944, 2929, 2837, 1718, 1659, 1601.

HR-MS (*m/z*): calcd. for $[M + H]^+ C_{24}H_{20}NO_4$, 386.1387; found 386.1379.

4'-(4-Methoxyphenyl)-3'-phenyl-6',6a'-dihydrospiro[cyclohexane-1,5'-cyclopenta[d]oxazole]-2,5-diene-2',4(3'H)-dione (48):

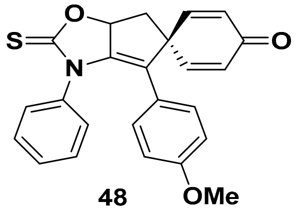

48

To a stirred solution of propargyl alcohol **44** (0.05 g, 0.17 mmol) in CH_2Cl_2 (2.0 mL) at rt was added Et_3N (0.03 mL, 0.21 mmol) followed by the addition of phenyl isothiocyanate (0.023 g, 0.17 mmol). The resulting light-yellow solution was stirred for 16 h. The reaction mixture was diluted with water (2 mL) and CH_2Cl_2 (5 mL). The two layers were separated, and aqueous portion was back extracted with CH_2Cl_2 (2 x 5 mL). The combined organic solutions were washed with brine (2.5 mL), dried over sodium sulfate and concentrated under reduced pressure. The crude material **46** thus obtained was used in the next step without further purification.

To a stirred solution propargyl thiourethane **46** (0.071 g, 0.17 mmol) in HFIP (3.4 mL) at rt was added Cs_2CO_3 (0.066 g, 0.203 mmol). After stirring the reaction mixture for 10 minutes, IBDA (0.082 g, 0.254 mmol) was added, and the reaction mixture was stirred for 1 h. The reaction mixture was diluted with water (5 mL) and CH_2Cl_2 (10 mL). The separated aqueous

layer was back extracted with CH_2Cl_2 (2 x 5 mL). The combined organic solutions were washed with brine (2.5 mL), dried over sodium sulfate and concentrated under reduced pressure. The crude product thus obtained was purified by column chromatography over silica gel (solid load, 0-80% EtOAc/ Hexanes as eluent) to afford the desired product **48**.

Physical appearance: Colorless gum.

Yield: 0.052 g, 78%

^{1}H NMR (500 MHz, Methanol-*d*$_4$): δ 7.70 (dt, *J* = 8.6, 1.4 Hz, 2H), 7.64 – 7.56 (m, 1H), 7.43 – 7.36 (m, 2H), 7.02 (dd, *J* = 10.0, 2.9 Hz, 1H), 7.01 – 6.95 (m, 2H), 6.92 (dd, *J* = 9.9, 2.9 Hz, 1H), 6.90 – 6.84 (m, 2H), 6.18 (ddd, *J* = 13.9, 10.0, 1.9 Hz, 2H), 5.34 (dd, *J* = 7.0, 4.3 Hz, 1H), 3.79 (s, 3H), 2.74 (dd, *J* = 13.5, 7.0 Hz, 1H), 2.30 (dd, *J* = 13.5, 4.3 Hz, 1H).

^{13}C NMR (125 MHz, Methanol-*d*$_4$): δ 185.5, 161.2, 157.3, 152.0, 151.2, 135.1, 131.8, 131.3, 129.3, 129.2, 129.0, 128.0, 127.5, 124.6, 115.3, 113.8, 76.6, 56.7, 54.6, 44.0.

FT-IR (neat, cm^{-1}): 2935, 2929, 2834, 1722, 1659, 1600.

3-Hydroxy-1-(4-methoxyphenyl)-2-(phenylamino)spiro[4.5]deca-1,6,9-trien-8-one (49):

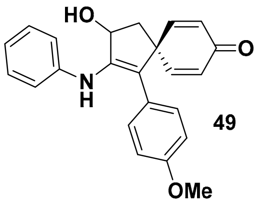

49

Suggested by NMR

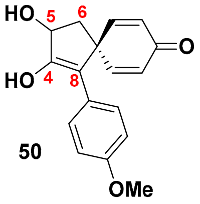

50

Suggested by mass spec.

In a small microwave vial (0.5 – 2 mL) at rt was added **47** (0.010 g, 0.026 mmol) and K_2CO_3 (0.007 g, 0.051 mmol) in MeOH (0.3 mL). The resulting suspension was heated to 60 °C for 1 h. The reaction mixture was diluted with water (2 mL) and CH_2Cl_2 (2.5 mL). The resulting two layers were separated and the aqueous solution was back extracted with CH_2Cl_2 (2 x 2.5 mL). The combined organic extracts were washed with brine (2.5 mL), dried over sodium sulfate and concentrated under reduced pressure. The crude thus obtained was purified by column chromatography over silica gel (solid load, 0-5% MeOH/ CH_2Cl_2 as eluent) to afford the desired product **49**.

Physical appearance: Colorless gum.

Yield: 0.007 g, 76%

^{1}H NMR (500 MHz, Methanol-d_4): δ 7.27 (dd, *J* = 10.1, 2.9 Hz, 1H), 7.05 (dd, *J* = 10.1, 2.9 Hz, 1H), 7.00 – 6.90 (m, 4H), 6.65 (td, *J* = 7.6, 1.1 Hz, 3H), 6.60 – 6.55 (m, 2H), 6.21 (ddd, *J* = 10.1, 5.6, 2.0 Hz, 2H), 5.10 (dd, *J* = 6.8, 4.2 Hz, 1H), 3.64 (s, 3H), 2.46 (dd, *J* = 13.4, 6.8 Hz, 1H), 2.01 (dd, *J* = 13.4, 4.3 Hz, 1H).

^{13}C NMR (126 MHz, Methanol-d_4): δ 187.1, 158.8, 158.5, 158.0, 142.1, 141.4, 128.4, 128.1, 127.9, 127.4, 126.9, 119.5, 118.1, 117.4, 112.9, 74.0, 54.2, 47.9, 42.9.

FT-IR (neat, cm^{-1}): 3232, 2943, 2929, 2838, 1718, 1702, 1659, 1600.

HR-MS (*m/z*): calcd. for $[M + H]^+$ Mass spectrometry data showed the mass of **50 ($[M+H]^+$ 285)**.

***N*-Allyl-4-cyclohexyl-1-(4-methoxyphenyl)-*N*-methylbut-3-yn-2-amine (51):**

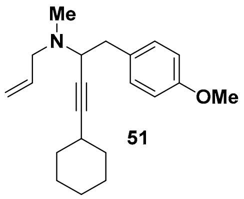

51

In a thick-walled, high-pressure tube (75 mL) were added aldehyde **42** (1.0 g, 6.65 mmol), and CH_3CN (25 mL). The solution was degassed with nitrogen for 15 minutes. To this were added *N*-allylmethylamine (1.0 mL, 9.98 mmol) and oven-dried molecular sieves (3 Å, 8-12 mesh, 3.0 g). Degassing was discontinued after another 5 minutes. The tube was sealed with a Teflon screw cap and placed in a preheated 90 °C oil-bath and left for 24 h. The reaction tube was removed from oil bath and allowed to cool to room temperature. To the reaction mixture was added CuBr (0.19 g, 1.33 mmol) followed by the addition of cyclohexylacetylene (1.0 g, 9.98 mmol), and the tube was resealed and returned to the 90 °C oil-bath for 48 h. The reaction tube was removed from oil bath and cooled to room temperature. The reaction mixture was filtered through Celite and rinsed with EtOAc (25 mL). The filtrate was washed with water (2 x 25 mL) and sat. NaCl solution (25 mL). The organic extracts were dried over Na_2SO_4 and concentrated under vacuum. The residue was purified by flash chromatography over silica gel (solid load, 0-10% EtOAc/ Hexanes as eluent) to afford propargyl amine **51.**

Physical appearance: Light yellow oil.

Yield: (0.87 g, 42%).

^{1}H NMR: δ 7.18 (d, *J* = 8.6 Hz, 2H), 6.82 (d, *J* = 8.6 Hz, 2H), 5.83 (dddd, *J* = 17.2, 10.1, 7.2, 5.8 Hz, 1H), 5.19 (dd, *J* = 17.1, 1.8 Hz, 1H), 5.11 (dd, *J* = 10.2, 1.8 Hz, 1H), 3.78 (s, 3H), 3.57 (ddd, *J* = 9.5, 5.4, 2.0 Hz, 1H), 3.14 (ddt, *J* = 13.4, 5.8, 1.6 Hz,

1H), 3.02 (ddt, *J* = 13.4, 7.1, 1.2 Hz, 1H), 2.85 (dd, *J* = 13.1, 5.5 Hz, 1H), 2.79 (dd, *J* = 13.1, 9.5 Hz, 1H), 2.42 – 2.36 (m, 1H), 2.26 (s, 3H), 1.79 – 1.72 (m, 2H), 1.68 (dtd, *J* = 13.0, 6.4, 3.5 Hz, 2H), 1.47 – 1.38 (m, 3H), 1.36 – 1.24 (m, 3H).

^{13}C NMR: δ 158.2, 136.3, 131.3, 130.5, 117.5, 113.6, 91.2, 76.5, 58.2, 58.0, 55.3, 39.9, 37.6, 33.1, 29.1, 26.1, 24.8.

FT-IR (neat, cm^{-1}): 2927, 2851, 2788, 2216, 1612, 1511, 1447, 1244.

HR-MS (*m/z*): calc for $[M+H]^+$ $C_{21}H_{30}NO$, 312.2322; found: 312.2317.

4-Cyclohexyl-1-(4-methoxyphenyl)-*N*-methylbut-3-yn-2-amine (52):

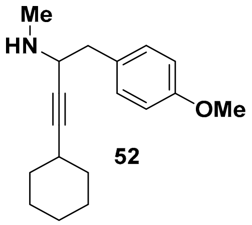

52

A stirred solution of **51** (0.50 g, 1.6 mmol) and *N,N*-dimethylbarbituric acid (0.30 g, 1.92 mmol) in anhydrous CH_2Cl_2 (16 mL) was degassed by purging nitrogen for 10 minutes. To this was added $Pd(PPh_3)_4$ (0.093 g, 0.080 mmol) in one portion and the reaction mixture was allowed to stir at 35 °C for 2.5 h. The reaction mixture was cooled to room temperature and the solvent was removed under vacuum. The crude material was purified by column chromatography over silica gel (solid load, 0-10% MeOH/ CH_2Cl_2 as eluent) to afford the deprotected amine **52**.

Physical appearance: Purple gum.

Yield: (0.31 g, 71%)

^{1}H NMR: δ 7.18 (d, *J* = 8.7 Hz, 2H), 6.81 (d, *J* = 8.6 Hz, 2H), 3.77 (s, 3H), 3.61 – 3.55 (m, 1H), 2.97 – 2.85 (m, 2H), 2.50 (s, 3H), 2.37 (s, 1H), 1.74 (d, *J* = 9.0 Hz, 2H), 1.65 (d, *J* = 9.8 Hz, 2H), 1.52 – 1.44 (m, 1H), 1.39 (d, *J* = 6.9 Hz, 2H), 1.31 – 1.23 (m, 3H).

^{13}C NMR: δ 158.5, 130.8, 129.4, 113.7, 79.6, 78.7, 55.3, 53.3, 40.8, 33.2, 32.8, 29.1, 26.0, 24.9.

FT-IR (neat, cm^{-1}): 3306, 2926, 2851, 2791, 2238, 1635, 1511, 1445, 1245.

HR-MS (*m/z*): calc for $[M+H]^+$ $C_{18}H_{26}NO$, 272.2009 found: 272.2000.

1-(4-Cyclohexyl)-1-(4-methoxyphenyl)but-3-yn-2-yl)-1,3-bisbutoxycarbonyl)-2-methylguanidine (53):

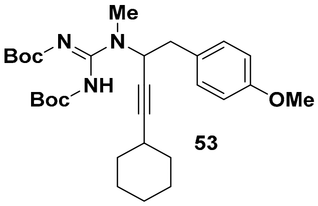

In a round bottom flask was added the 2-methyl-2-pseudothiourea (0.214 g, 0.736 mmol), HgO (0.16 g, 0.736 mmol), Et_3N (1.2 mL, 0.736 mmol) and anhydrous CH_2Cl_2 (5.0 mL). The reaction mixture was allowed to stir for 10 min. A solution of *N*-methyl propargylamine **52** (0.20 g, 0.736 mmol) in anhydrous CH_2Cl_2 (2.0 mL) was added and reaction mixture was allowed to stir for additional 16 h. The reaction mixture was filtered through Celite and solvent was removed under vacuum. The obtained crude was purified by column chromatography over silica gel (solid load, 0-10% EtOAc/ Hexanes as eluent) to afford corresponding guanidine **53**.

Physical appearance: Colorless solid.

m. p. = 102-106 °C.

Yield: (0.307 g, 73%)

^{1}H NMR: δ 7.21 (d, *J* = 8.6 Hz, 2H), 6.80 (d, *J* = 8.6 Hz, 2H), 3.77 (s, 3H), 3.01 (s, 4H), 2.87 (dd, *J* = 12.8, 9.5 Hz, 1H), 2.33 (d, *J* = 14.1 Hz, 1H), 1.75 – 1.65 (m, 2H), 1.60 (ddt, *J* = 9.4, 6.6, 3.1 Hz, 2H), 1.47 (s, 19H), 1.36 (dq, *J* = 13.4, 4.7 Hz, 2H), 1.29 – 1.19 (m, 3H).

^{13}C NMR: δ 162.5, 158.5, 154.8, 150.8, 130.9, 129.4, 113.6, 91.2, 81.9, 79.3, 76.6, 55.3, 39.4, 32.9, 32.6, 29.0, 28.3, 28.2, 25.9, 24.7.

FT-IR (neat, cm^{-1}): 2977, 2930, 2851, 1746, 1639, 1599, 1288, 1136.

HR-MS (*m/z*): calc for $[M+H]^+$ $C_{29}H_{44}N_3O_5$, 514.3275; found: 514.3269.

1-(4-Methoxyphenyl)-4-(trimethylsilyl)but-3-yn-2-ol (58):

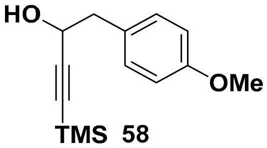

To a stirred solution of TMS acetylene (0.50 g, 5.09 mmol) in anhydrous THF (15 mL) at -78 °C was added *n*-BuLi (3.4 mL, 5.09 mmol, 1.5 M in hexanes) dropwise. The resulting light-yellow solution was allowed to warm to 0 °C and stirred for 30 minutes. The solution was cooled back to -78 °C and to this was added dropwise, a solution of aldehyde (0.382 g, 2.54 mmol, dissolved in 3 mL THF). The reaction mixture was allowed to warm to rt and stirred for 30 minutes. The mixture

was diluted with saturated ammonium chloride solution (10 mL) and EtOAc (50 mL). Two layers were separated and aqueous was back extracted with EtOAc (2 x 15 mL). The combined organic layer was washed with brine (10 mL), dried over sodium sulfate and concentrated under reduced pressure. The crude thus obtained was purified by column chromatography over silica gel (solid load, 0-20% EtOAc/ Hexanes as eluent) to afford the desired product **58**.

Physical appearance: Orange gum.

Yield: 0.52 g, 83%

^{1}H NMR: δ 7.24 – 7.13 (m, 2H), 6.92 – 6.80 (m, 2H), 4.50 (q, *J* = 6.1 Hz, 1H), 3.79 (s, 3H), 3.00 – 2.87 (m, 2H), 1.98 (d, *J* = 5.8 Hz, 1H), 0.16 (s, 9H).

^{13}C NMR: δ 158.7, 131.0, 128.5, 113.9, 106.2, 90.5, 63.8, 55.4, 43.2, 0.1.

FT-IR (neat, cm^{-1}): 3420, 2957, 2901, 2836, 2171, 1712, 1612, 1511.

HR-MS (*m/z*): calcd. for $[M + Na]^+ C_{14}H_{20}O_2SiNa$, 271.1125; found 271.1123.

1-(4-Methoxyphenyl)-5-(trimethylsilyl)pent-4-yn-2-ol (59):

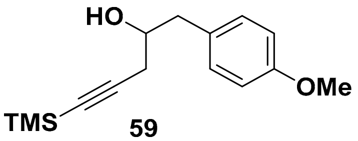

59

To a stirred solution of TMS propyne (0.25 g, 2.22 mmol) in anhydrous THF (7.5 mL) at -78 °C was added *n*-BuLi (1.5 mL, 2.22 mmol, 1.5M in hexanes) dropwise. The resulting light yellow solution was allowed to warm to 0 °C and stirred for 30 minutes. The solution was cooled back to -78 °C and to this was added dropwise, a solution of aldehyde (0.17 g, 1.11 mmol, dissolved in 1.5 mL THF). The reaction mixture was allowed to warm to rt and stirred for 30 minutes. The mixture was diluted with saturated ammonium chloride solution (5.0 mL) and EtOAc (20 mL). Two layers were separated and aqueous was back extracted with EtOAc (2 x 10 mL). The combined organic layer was washed with brine (10 mL), dried over sodium sulfate and concentrated under reduced pressure. The crude thus obtained was purified by column chromatography over silica gel (solid load, 0-20% EtOAc/ Hexanes as eluent) to afford the desired product **59**.

Physical appearance: Yellow oil.

Yield: 0.124 g, 42%

^{1}H NMR: δ 7.17 – 7.12 (m, 2H), 6.89 – 6.81 (m, 2H), 3.96 – 3.87 (m, 1H), 3.78 (s, 3H), 2.84 (dd, *J* = 13.7, 5.6 Hz, 1H), 2.76 (dd, *J* = 13.7, 7.2 Hz, 1H), 2.47 – 2.34 (m, 2H), 2.07 (d, *J* = 4.8 Hz, 1H), 0.17 (s, 9H).

^{13}C NMR: δ 158.5, 130.5, 129.9, 114.1, 103.2, 87.9, 71.1, 55.3, 41.6, 31.7, 27.9.

FT-IR (neat, cm^{-1}): 3445, 3293, 2956, 2907, 2835, 2173, 1719, 1611, 1510.

HR-MS (*m/z*): calcd. for $[M + Na]^+ C_{15}H_{22}O_2SiNa$, 285.1281; found 285.1286.

1-(4-Methoxyphenyl)-4-(trimethylsilyl)but-3-yn-2-yl phenylcarbamate (60):

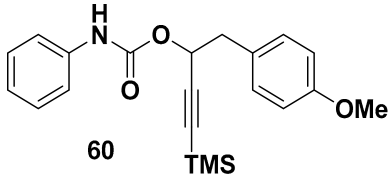

60

To a stirred solution propargyl alcohol **58** (0.05 g, 0.20 mmol) in CH_2Cl_2 (2.0 mL) at rt was added Et_3N (0.0335 mL, 0.24 mmol) followed by the addition of phenyl isocyanate (0.0264 g, 0.22 mmol). The resulting light-yellow solution was stirred for 16 h. The reaction mixture was diluted with water (2 mL) and CH_2Cl_2 (5 mL). Two layers were separated and aqueous was back extracted with CH_2Cl_2 (2 x 5 mL). The combined organic layer was washed with brine (2.5 mL), dried over sodium sulfate and concentrated under reduced pressure. The crude thus obtained was purified by column chromatography over silica gel (solid load, 0-20% EtOAc/ Hexanes as eluent) to afford the desired product **60**.

Physical appearance: Colorless solid.

m.p. = 102-104 °C

Yield: 0.051 g, 69%

^{1}H NMR: δ 7.36 (d, *J* = 8.1 Hz, 2H), 7.32 – 7.28 (m, 2H), 7.21 (d, *J* = 8.6 Hz, 2H), 7.07 (tt, *J* = 7.3, 1.2 Hz, 1H), 6.84 (d, *J* = 8.7 Hz, 2H), 6.68 (s, 1H), 5.62 – 5.54 (m, 1H), 3.79 (s, 3H), 3.05 (dq, *J* = 13.7, 7.2 Hz, 2H), 0.17 (s, 9H).

^{13}C NMR: δ 158.8, 152.2, 137.7, 131.0, 129.2, 127.9, 123.7, 118.8, 113.8, 102.3, 92.0, 66.2, 55.3, 40.6, -0.2.

FT-IR (neat, cm^{-1}): 3269, 3135, 3069, 3038, 2951, 2835, 2191, 1694, 1609, 1541.

HR-MS (*m/z*): calcd. for $[M + H]^+ C_{21}H_{26}NO_3Si$, 368.1676; found 368.1679.

1-(4-Methoxyphenyl)-5-(trimethylsilyl)pent-4-yn-2-yl phenylcarbamate (61):

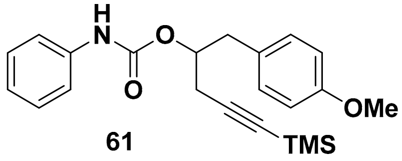

61

To a stirred solution propargyl alcohol **152** (0.05 g, 0.19 mmol) in CH_2Cl_2 (2.0 mL) at rt was added Et_3N (0.033 mL, 0.23 mmol) followed by the addition of phenyl isocyanate (0.025 g, 0.21 mmol). The resulting light yellow solution was stirred for 16 h. The reaction mixture was diluted with water (2 mL) and CH_2Cl_2 (5 mL). Two layers were separated and aqueous was back extracted with CH_2Cl_2 (2 x 5 mL). The combined organic layer was washed with brine (2.5 mL), dried over sodium sulfate and concentrated under reduced pressure. The crude thus obtained was used in the next step without further purification.

Physical appearance: Yellow oil.

Yield: 0.07 g, quantitative (crude).

^{1}H NMR: δ 7.22 – 7.13 (m, 2H), 7.06 (tt, *J* = 7.4, 1.0 Hz, 2H), 6.97 – 6.88 (m, 2H), 6.83 – 6.73 (m, 1H), 6.65 – 6.55 (m, 2H), 3.68 (h, *J* = 5.8 Hz, 1H), 2.65 (dd, *J* = 13.7, 5.9 Hz, 1H), 2.52 (dd, *J* = 13.7, 6.9 Hz, 1H), 2.14 (d, *J* = 5.9 Hz, 2H), -0.02 – -0.12 (m, 9H).

FT-IR (neat, cm^{-1}): 3266, 3132, 3000, 2951, 2835, 2185, 1609.

HR-MS (*m/z*): calcd. for $[M + Na]^+C_{22}H_{27}NO_3SiNa$, 404.1652; found 404.1654.

(Z)-1,4-bis(4-Methoxyphenyl)but-3-en-2-ol (65):

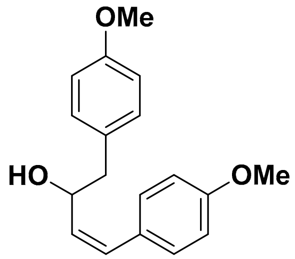

65

To a stirred solution propargyl alcohol **44** (0.04 g, 0.14 mmol) and ethylene diamine (0.0085 g, 0.141 mmol) in EtOH (0.75 mL) at 0 °C was added $Ni(OAc)_2.4H_2O$ (0.012 g, 0.042 mmol) followed by the addition of $NaBH_4$ (0.003 g, 0.085 mmol, dissolved in 0.375 mL of EtOH). The resulting light-yellow solution was stirred at this temperature for 6 h. The reaction mixture was diluted with water (2 mL) and CH_2Cl_2 (5 mL). Two layers were separated and aqueous was back extracted with CH_2Cl_2 (2 x 5 mL). The combined organic layer was washed with brine (2.5 mL), dried over sodium sulfate and concentrated under reduced pressure. The crude thus obtained was purified by column chromatography over silica gel (solid load, 0-20% EtOAc/ Hexanes as eluent) to afford the desired product **65**.

Physical appearance: Light yellow gum.

Yield: 0.031 g, 78%.

^{1}H NMR: δ 7.19 – 7.14 (m, 4H), 6.87 – 6.82 (m, 4H), 6.51 (d, *J* = 11.6 Hz, 1H), 5.63 (dd, *J* = 11.6, 9.1 Hz, 1H), 4.74 (q, *J* = 7.6 Hz, 1H), 3.80 (d, *J* = 1.0 Hz, 3H), 3.79 (s, 3H), 2.90 (dd, *J* = 13.8, 4.9 Hz, 1H), 2.82 (dd, *J* = 13.8, 7.8 Hz, 1H), 1.74 – 1.69 (m, 1H).

^{13}C NMR (126 MHz, Methanol-*d*$_4$): δ 158.9, 158.4, 130.3, 130.1, 130.0, 129.7, 113.4, 113.2, 78.3, 68.6, 57.0, 54.4, 43.0, 17.1.

FT-IR (neat, cm^{-1}): 3450, 3000, 2931, 2909, 2834, 1606, 1508.

HR-MS (*m/z*): calcd. for $[M + H]^+$ $C_{18}H_{21}O_3$, 285.1490, found 285.1487.

(*Z*)-1,4-bis(4-Methoxyphenyl)but-3-en-2-yl phenylcarbamate (66):

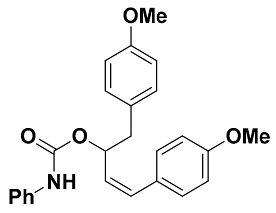

66

To a stirred solution propargyl alcohol **65** (0.035 g, 0.123 mmol) in CH_2Cl_2 (2.0 mL) at rt was added Et_3N (0.017 mL, 0.123 mmol) followed by the addition of phenylisocyanate (0.013 mL, 0.123 mmol). The resulting light-yellow solution was stirred for 16 h. The reaction mixture was diluted with water (2 mL) and CH_2Cl_2 (5 mL). Two layers were separated and aqueous was back extracted with CH_2Cl_2 (2 x 5 mL). The combined organic layer was washed with brine (2.5 mL), dried over sodium sulfate and concentrated under reduced pressure. The crude thus obtained was purified by column chromatography over silica gel (solid load, 0-40% EtOAc/ Hexanes as eluent) to afford the desired product **66** (9:1 *Z:E*).

Physical appearance: Colorless gum.

Yield: 0.035 g, 72%.

^{1}H NMR: δ 7.36 – 7.24 (m, 3H), 7.23 – 7.01 (m, 6H), 6.88 – 6.77 (m, 4H), 6.62 (s, 1H), 6.52 (d, *J* = 11.8 Hz, 1H), 5.94 (dtd, *J* = 9.2, 6.3, 1.1 Hz, 1H), 5.57 (dd, *J* = 11.8, 9.2 Hz, 1H), 3.85 – 3.72 (m, 6H), 3.06 (dd, *J* = 14.0, 6.3 Hz, 1H), 2.95 (dd, *J* = 13.9, 6.3 Hz, 1H), 2.05 (s, 1H), 1.27 (t, *J* = 7.1 Hz, 1H).

^{13}C NMR: δ 159.0, 158.5, 152.9, 138.0, 132.0, 130.8, 130.6, 130.0, 129.7, 129.4, 129.2, 129.1, 129.0, 128.8, 128.1, 125.9, 124.9, 123.5, 118.8, 113.9, 113.9, 72.7, 55.3, 40.4.

FT-IR (neat, cm^{-1}): 3266, 3136, 2951, 2850, 1693, 1610.

HR-MS (*m/z*): calcd. for $[M - H]^- C_{25}H_{24}NO_4$, 402.1711; found 402.1715.

Benzyl (Z)-2'-(((benzyloxy)carbonyl)imino)-3a'-(tert-butylperoxy)-6'-(4-methoxyphenyl)-3'-methyl-4-oxo-2',3',3a',4'-tetrahydro-1'H-spiro[cyclohexane-1,5'-cyclopenta[*d*]imidazole]-2,5-diene-1'-carboxylate (69):

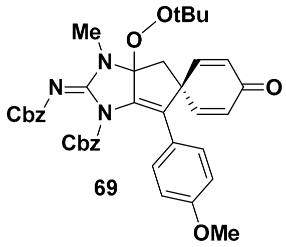

69

To a stirred solution of **20** (0.20 g, 0.34 mmol) in anhydrous CH_2Cl_2 (4.0 mL) at 0 °C was added K_2CO_3 (0.023 g, 0.17 mmol) followed by the addition of TBHP (0.1 mL, 0.51 mmol, 5.1M in decane). After stirring at this temperature was added $Pd(OH)_2$ (60 mg, 20% on C). The resulting black suspension was allowed to warm to rt and stirred for 1 h. The reaction mixture was filtered through celite, diluted with water (2 mL) and CH_2Cl_2 (10 mL). Two layers were separated and aqueous was back extracted with EtOAc (2 x 5 mL). The combined organic layer was washed with brine (2.5 mL), dried over sodium sulfate and concentrated under reduced pressure. The crude thus obtained was purified by column chromatography over silica gel (solid load, 0-25% EtOAc/ Hexanes as eluent) to afford the desired product **69**.

Physical appearance: Colorless oil.

Yield: 0.057 g, 25%

^{1}H NMR: δ 7.48 (dd, *J* = 10.1, 2.9 Hz, 1H), 7.45 – 7.27 (m, 5H), 7.25 – 7.18 (m, 1H), 7.21 – 7.10 (m, 2H), 7.07 – 6.91 (m, 2H), 6.88 – 6.81 (m, 2H), 6.61 – 6.53 (m, 2H), 6.52 (dd, *J* = 10.0, 2.9 Hz, 1H), 6.42 (dd, *J* = 10.1, 1.9 Hz, 1H), 6.21 (dd, *J* = 10.0, 1.9 Hz, 1H), 5.19 (d, *J* = 12.1 Hz, 1H), 5.15 – 5.06 (m, 1H), 4.88 (d, *J* = 12.0 Hz, 1H), 4.81 (d, *J* = 12.0 Hz, 1H), 3.74 (s, 3H), 2.98 (s, 3H), 2.52 (d, *J* = 13.2 Hz, 1H), 2.16 (d, *J* = 13.3 Hz, 1H), 1.09 (s, 9H).

^{13}C NMR: δ 185.3, 159.8, 159.7, 153.2, 152.8, 152.1, 149.5, 136.4, 134.6, 134.3, 131.4, 130.0, 128.9, 128.8, 128.6, 128.5, 128.4, 128.3, 128.0, 124.7, 113.6, 100.1, 80.9, 68.6, 68.1, 55.9, 55.2, 45.4, 27.5, 26.4.

FT-IR (neat, cm^{-1}): 3305, 2961, 2930, 1708, 1654, 1600.

HR-MS (*m/z*): calcd. for $[M + H]^+ C_{39}H_{40}N_3O_8$, 678.2815, found 678.2820.

Benzyl (Z)-2'-(((benzyloxy)carbonyl)imino)-3a'-(tert-butylperoxy)-6'-(4-methoxyphenyl)-3'-methyl-5-oxo-2',3',3a',4'-tetrahydro-1'H-7-oxaspiro[bicyclo[4.1.0]heptane-2,5'-cyclopenta[d]imidazol]-3-ene-1'-carboxylate (70):

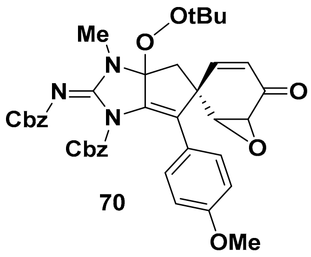

70

Mixture of diastereomers

To a stirred solution of **69** (0.025 g, 0.037 mmol) in acetone (0.4 mL) at 0 °C was added DMDO (0.04M in acetone, 0.044 mmol, 1.1 mL). The resulting light-yellow solution was allowed to warm to rt and stirred for 72 h. The reaction mixture was concentrated under reduced pressure. The crude thus obtained was purified by column chromatography column chromatography over silica gel (solid load, 0-50% EtOAc/ Hexanes as eluent) to afford the desired product **70** as mixture of two isomers.

Physical appearance: Colorless oil.

Yield: 0.01 g, 40%, 1:1 mixture of two isomers.

^{1}H NMR: δ 7.40 – 7.36 (m, 2H), 7.36 – 7.30 (m, 3H), 7.24 – 7.20 (m, 1H), 7.20 – 7.14 (m, 2H), 7.01 – 6.97 (m, 1H), 6.94 (dd, *J* = 10.4, 2.7 Hz, 1H), 6.88 – 6.84 (m, 3H), 6.62 – 6.59 (m, 2H), 6.07 (dd, *J* = 10.5, 2.0 Hz, 0.5H), 5.94 (dd, *J* = 10.5, 2.0 Hz, 0.5H), 5.78 (dd, *J* = 10.5, 2.0 Hz, 0.5H), 5.16 (d, *J* = 12.1 Hz, 1H), 5.08 (dd, *J* = 12.1, 2.3 Hz, 1H), 4.85 (dd, *J* = 12.0, 6.4 Hz, 1H), 4.72 (dd, *J* = 12.0, 9.0 Hz, 1H), 3.76 (s, 1.5H), 3.75 (s, 1.5H), 3.67 (dd, *J* = 3.6, 2.0 Hz, 0.5H), 3.23 (dd, *J* = 3.9, 2.8 Hz, 0.5H), 3.17 (dd, *J* = 3.6, 2.0 Hz, 0.5H), 3.01 (s, 3H), 2.69 (d, *J* = 13.5 Hz, 0.5H), 2.63 (d, *J* = 13.5 Hz, 0.5H), 2.32 (dd, *J* = 15.5, 13.6 Hz, 1H), 1.08 (s, 9H).

^{13}C NMR (126 MHz, Benzene-*d*$_6$): δ 192.6, 192.4, 159.9, 159.7, 149.6, 149.5, 148.8, 148.1, 137.4, 137.3, 134.8, 134.7, 130.5, 129.8, 129.2, 128.7, 128.6, 128.5, 128.4, 128.3, 128.2, 128.1, 128.0, 126.5, 125.0, 124.6, 124.4, 113.8, 113.7, 100.0, 99.7, 80.4, 80.3, 68.2, 68.1, 67.8, 67.7, 60.7, 60.2, 59.8, 55.2, 54.6, 54.5, 54.1, 53.1, 43.9, 42.9, 29.9, 29.8, 26.8, 26.0, 20.3, 14.0.

FT-IR (neat, cm^{-1}): 3304, 2953, 2924, 2852, 1707, 1599.

HR-MS (*m/z*): calcd. for $[M + H]^+ C_{39}H_{40}N_3O_9$, 694.2759: found 694.2751.

Benzyl (Z)-2'-(((benzyloxy)carbonyl)imino)-4'-bromo-6'-(4-methoxyphenyl)-3'-methyl-4-oxo-2',3'-dihydro-1'H-spiro[cyclohexane-1,5'-cyclopenta[d]imidazole]-2,5-diene-1'-carboxylate (71):

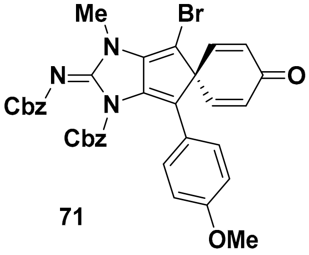

71

To a stirred suspension of *tert*-butyl peroxybenzoate (0.05 g, 0.26 mmol) and CuBr (0.012 g, 0.084 mmol) in anhydrous benzene (1.0 mL) at rt was added **20** (0.050 g, 0.084 mmol). The resulting black suspension was stirred for 1 h. The reaction mixture was filtered through Celite, diluted with water (2 mL) and CH_2Cl_2 (10 mL). Two layers were separated and aqueous was back extracted with EtOAc (2 x 5 mL). The combined organic layer was washed with brine (2.5 mL), dried over sodium sulfate and concentrated under reduced pressure. The crude thus obtained was purified by column chromatography over silica gel (solid load, 0-30% EtOAc/ Hexanes as eluent) to afford the desired product **71**.

Physical appearance: Black solid.

Yield: 0.0452 g, 80%.

^{1}H NMR: δ 7.40 – 7.31 (m, 5H), 7.24 (dt, *J* = 23.1, 8.0 Hz, 4H), 6.95 (dd, *J* = 18.1, 7.9 Hz, 4H), 6.65 (d, *J* = 8.7 Hz, 2H), 6.45 (d, *J* = 9.9 Hz, 2H), 6.34 (d, *J* = 9.9 Hz, 2H), 5.13 (s, 2H), 4.67 (s, 2H), 3.75 (s, 3H), 3.41 (s, 3H).

^{1}H NMR (500 MHz, Benzene-*d*$_6$): δ 7.38 – 7.32 (m, 2H), 7.15 – 7.05 (m, 3H), 7.04 – 6.95 (m, 1H), 6.98 – 6.79 (m, 6H), 6.47 – 6.40 (m, 2H), 6.37 – 6.31 (m, 2H), 5.79 – 5.72 (m, 2H), 5.30 (s, 2H), 4.51 (s, 2H), 3.18 (s, 3H), 2.71 (s, 3H).

^{13}C NMR: δ 185.6, 159.4, 158.4, 150.2, 149.3, 145.8, 139.4, 136.1, 133.8, 133.0, 132.6, 129.7, 129.1, 128.9, 128.7, 128.5, 128.4, 128.3, 125.3, 122.6, 113.3, 83.3, 69.5, 68.4, 67.5, 55.3, 29.5.

^{13}C NMR (126 MHz, Benzene-*d*$_6$): δ 184.2, 159.4, 158.2, 149.3, 145.0, 138.8, 136.9, 134.2, 132.7, 132.4, 129.6, 129.0, 128.7, 128.6, 128.4, 128.3, 128.0, 125.6, 121.8, 113.1, 82.7, 68.9, 68.0, 66.9, 54.5, 28.4.

FT-IR (neat, cm^{-1}): 2959, 2921, 2850, 1758, 1702, 1661, 1601.

HR-MS (*m/z*): calcd. for $[M + H]^{+}C_{35}H_{29}N_3O_6Br$, 666.1234; found 666.1254.

Figure S1, Structure 8, Method: B3LYP-D3BJ/6-311G(d,p), Energy/Hartree=-1202.2009235

C	-6.15497	-1.30783	-0.06231
C	-3.81401	-1.10588	0.34761
C	-3.52700	-0.84503	-0.99235
C	-2.20700	-0.60913	-1.37443
C	-1.15717	-0.62456	-0.45810
C	-1.46721	-0.88984	0.88431
C	-2.77174	-1.13046	1.28042
C	0.23198	-0.30110	-0.93454
C	1.39466	-1.23092	-0.54364
C	2.69557	-0.38477	-0.85540
C	2.23387	1.05822	-1.10883
C	0.75264	1.15630	-0.61987
C	-0.01464	2.13613	-1.45067
C	-0.73807	3.14306	-0.95397
C	-0.81714	3.39902	0.49755
C	-0.02587	2.49022	1.34855
C	0.68602	1.47740	0.84467
C	2.64188	-1.24656	1.27625
C	4.73755	0.00489	0.63681
H	-6.25159	-0.32103	-0.52869
H	-6.05964	-2.06957	-0.84449
H	-7.04260	-1.51321	0.53348
H	-0.67014	-0.93383	1.61067
H	-3.01451	-1.33827	2.31524
H	-4.30814	-0.82180	-1.73931
H	-1.99603	-0.40643	-2.41906
H	0.22104	-0.36098	-2.02803
H	1.24402	0.83566	1.51441
H	-0.05787	2.69121	2.41340
H	-1.29826	3.81907	-1.58979
H	0.02378	1.96916	-2.52446
H	2.26967	1.22750	-2.18550
H	2.86819	1.80372	-0.62823
H	4.70355	0.93185	1.22086
H	5.19128	0.20778	-0.33341
H	5.38895	-0.71031	1.14876
H	3.12944	-1.78264	-2.06094
H	1.24090	-3.12703	-0.76898
H	4.03708	-1.43711	2.76048
H	2.50434	-2.10789	3.07813
O	-1.48333	4.31248	0.96179
O	-5.06168	-1.35001	0.84065
O	1.38751	-2.39247	-1.37682
O	3.41951	-0.86142	-1.95120
N	1.48100	-1.63749	0.84162
N	3.42869	-0.55808	0.40086
N	3.04808	-1.42721	2.57006

Figure S1, Structure 9, Method: B3LYP-D3BJ/6-311G(d,p), Energy/Hartree=-1202.1989373

C	6.16882	-1.07501	0.82342
C	4.20416	-0.28834	-0.27817
C	3.35464	-1.02281	0.55213
C	1.97454	-0.95470	0.36932
C	1.40981	-0.16136	-0.62991
C	2.27965	0.56146	-1.45483
C	3.65421	0.50504	-1.28890
C	-0.08000	0.01658	-0.80959
C	-0.92451	-1.27877	-0.68877
C	-1.48298	-1.31135	0.79099
C	-1.10376	0.02268	1.43551
C	-0.72359	0.99642	0.27060
C	0.25041	2.01621	0.78173
C	0.04102	3.33631	0.76114
C	-1.18737	3.92565	0.19747
C	-2.16124	2.95967	-0.34406
C	-1.95324	1.63909	-0.31097
C	-3.12103	-1.46175	-0.81820
C	-3.86205	-1.86014	1.55620
H	5.85357	-0.75800	1.82388
H	5.93823	-2.13809	0.69176
H	7.24166	-0.92354	0.71916
H	1.86783	1.18887	-2.23774
H	4.32411	1.06525	-1.92917
H	3.74992	-1.65472	1.33532
H	1.34217	-1.54719	1.01663
H	-0.25382	0.43563	-1.80042
H	-2.68893	0.97460	-0.74321
H	-3.05926	3.38472	-0.77863
H	0.76806	4.03585	1.15800
H	1.16766	1.62158	1.20359
H	-0.22816	-0.14314	2.06320
H	-1.89501	0.43737	2.06065
H	-4.39534	-1.01075	1.99790
H	-3.30690	-2.37671	2.34023
H	-4.58910	-2.56206	1.14203
H	-0.67212	-3.02533	0.87728
H	-0.53595	-2.83009	-1.73347
H	-5.15277	-1.29494	-0.78229
H	-4.44490	-1.48170	-2.31304
O	-1.38562	5.13198	0.17782
O	5.56433	-0.28309	-0.18706
O	-0.16913	-2.46724	-0.91798
O	-0.98465	-2.38508	1.54002
N	-2.07579	-1.28663	-1.56937
N	-2.92251	-1.45080	0.53291
N	-4.38101	-1.65677	-1.32136

Figure S1, Structure 10, Method: B3LYP-D3BJ/6-311G(d,p), Energy/Hartree=-1202.1618582

C	-6.04910	-1.77770	-0.17402
C	-3.66806	-1.85905	-0.05715
C	-3.52792	-0.54824	-0.51438
C	-2.25364	0.00057	-0.65016
C	-1.10540	-0.72202	-0.33094
C	-1.26501	-2.04219	0.10889
C	-2.52458	-2.60421	0.24476
C	0.26316	-0.11959	-0.53316
C	1.44415	-0.81541	0.13762
C	2.57935	0.20274	-0.11247
C	2.00545	1.45345	0.51480
C	0.46947	1.38607	0.02145
C	0.26088	2.37010	-1.09055
C	-0.52312	3.44871	-0.99495
C	-1.28574	3.75350	0.23467
C	-1.16465	2.76642	1.32172
C	-0.39458	1.67864	1.21270
C	3.30328	-1.89733	-0.01833
C	5.06097	-0.12931	0.17333
H	-6.14514	-0.88627	0.45613
H	-6.08847	-1.47959	-1.22793
H	-6.87146	-2.45890	0.03797
H	-0.39196	-2.64184	0.32671
H	-2.65013	-3.62507	0.58416
H	-4.39036	0.05322	-0.76587
H	-2.16597	1.01605	-1.01469
H	0.47655	-0.08561	-1.60108
H	-0.33465	0.94995	2.01147
H	-1.75453	2.96117	2.21012
H	-0.64374	4.14724	-1.81544
H	0.80173	2.16375	-2.00923
H	2.47730	2.38313	0.18937
H	2.04749	1.39474	1.59855
H	5.74785	-0.77106	0.72841
H	5.14700	0.87202	0.59816
H	5.36681	-0.09112	-0.88067
H	3.09846	1.29392	-1.65460
H	1.41921	-1.70598	1.85759
H	5.16572	-2.65446	-0.36490
H	3.87622	-3.75605	-0.45182
O	-1.98080	4.75518	0.32950
O	-4.86220	-2.49662	0.11940
O	1.15322	-0.83434	1.55007
O	2.72788	0.41545	-1.52860
N	2.03309	-2.08978	-0.22220
N	3.69826	-0.59614	0.35367
N	4.23404	-2.89514	-0.06552

Figure S1, Structure 11, Method: B3LYP-D3BJ/6-311G(d,p), Energy/Hartree=-1202.1573237

C	5.09788	-3.15025	0.63764
C	3.63674	-1.42811	-0.12932
C	2.49031	-2.16633	0.16230
C	1.22785	-1.62645	-0.09116
C	1.08229	-0.35509	-0.63723
C	2.24851	0.36329	-0.94419
C	3.50664	-0.15272	-0.68972
C	-0.24382	0.28817	-0.96375
C	-1.55396	-0.51095	-0.90943
C	-2.07804	-0.28746	0.51885
C	-2.07424	1.22038	0.61722
C	-0.65675	1.57931	-0.04679
C	0.35539	1.83111	1.03109
C	0.95430	3.00955	1.22785
C	0.70086	4.17462	0.35760
C	-0.23931	3.94671	-0.75700
C	-0.85026	2.77095	-0.93569
C	-2.77042	-2.20420	-0.36756
C	-3.93017	-1.48482	1.71962
H	4.64330	-3.23027	1.63176
H	4.68107	-3.92819	-0.01212
H	6.17432	-3.28985	0.72184
H	2.16405	1.35078	-1.38481
H	4.40464	0.40631	-0.92124
H	2.55957	-3.16016	0.58288
H	0.35202	-2.21364	0.13555
H	-0.16492	0.66389	-1.98283
H	-1.54612	2.61887	-1.75444
H	-0.40951	4.78877	-1.41838
H	1.67534	3.15843	2.02366
H	0.60055	0.98430	1.66060
H	-2.13207	1.60975	1.63618
H	-2.88362	1.64387	0.02910
H	-4.88157	-1.97740	1.50773
H	-4.16940	-0.54591	2.22139
H	-3.34272	-2.11539	2.39602
H	-1.20600	-0.31392	2.27316
H	-2.69475	-0.43727	-2.46223
H	-4.30944	-3.49471	0.00432
H	-3.11404	-4.04149	-1.07987
O	1.24142	5.25526	0.54393
O	4.91476	-1.85761	0.08453
O	-2.43854	0.21694	-1.80346
O	-1.13420	-0.81806	1.45773
N	-1.76990	-1.92277	-1.15187
N	-3.23114	-1.17819	0.48042
N	-3.35483	-3.43552	-0.31029

Figure S1, Structure 8, Method: WB97XD-def2tzvp, Energy/Hartree=-1201.8656019

C	-6.17028	-1.27467	-0.04029
C	-3.85509	-0.99725	0.37851
C	-3.55132	-0.86800	-0.96971
C	-2.23293	-0.65600	-1.35296
C	-1.20074	-0.56716	-0.43154
C	-1.52902	-0.70295	0.91958
C	-2.83064	-0.91524	1.31944
C	0.19575	-0.30138	-0.91821
C	1.30634	-1.29257	-0.53323
C	2.64273	-0.51864	-0.85409
C	2.25004	0.93599	-1.13366
C	0.78826	1.11994	-0.63438
C	0.07504	2.12738	-1.47680
C	-0.53609	3.21040	-1.00845
C	-0.54414	3.52832	0.43064
C	0.17158	2.57727	1.29901
C	0.76594	1.48958	0.81749
C	2.54740	-1.33812	1.27698
C	4.64339	-0.11139	0.66839
H	-6.27589	-0.34432	-0.60741
H	-6.05067	-2.10948	-0.73847
H	-7.06630	-1.43340	0.55554
H	-0.74877	-0.66131	1.66457
H	-3.08052	-1.02052	2.36710
H	-4.31897	-0.92856	-1.72764
H	-2.01106	-0.55806	-2.40975
H	0.17018	-0.37152	-2.01071
H	1.27044	0.82001	1.50307
H	0.18400	2.81067	2.35646
H	-1.05098	3.90534	-1.65992
H	0.06334	1.91952	-2.54338
H	2.28142	1.08286	-2.21287
H	2.92830	1.65801	-0.67904
H	4.57005	0.78721	1.28921
H	5.10154	0.15279	-0.28349
H	5.31560	-0.82314	1.15416
H	3.02382	-1.94486	-2.04861
H	1.03781	-3.17171	-0.74304
H	3.92607	-1.53999	2.77166
H	2.36750	-2.14455	3.09846
O	-1.09698	4.51587	0.87112
O	-5.09992	-1.20495	0.86958
O	1.22118	-2.44706	-1.34697
O	3.34417	-1.03913	-1.93418
N	1.37986	-1.68931	0.84866
N	3.36169	-0.70262	0.39702
N	2.94390	-1.51606	2.56680

Figure S1, Structure 9, Method: WB97XD-def2tzvp, Energy/Hartree=-1201.8619656

C	6.16679	-0.99371	0.83132
C	4.20481	-0.26372	-0.27997
C	3.36716	-0.96220	0.58213
C	1.99142	-0.90545	0.40643
C	1.41706	-0.16000	-0.61406
C	2.27575	0.52676	-1.46979
C	3.64616	0.48232	-1.31317
C	-0.07352	-0.00557	-0.79678
C	-0.89679	-1.31269	-0.66600
C	-1.47825	-1.32332	0.79976
C	-1.12799	0.02657	1.42027
C	-0.73352	0.97739	0.25279
C	0.21864	2.01051	0.77145
C	-0.00268	3.32169	0.75183
C	-1.23013	3.89433	0.17508
C	-2.18040	2.92025	-0.38834
C	-1.95321	1.61007	-0.35374
C	-3.08266	-1.50668	-0.82092
C	-3.88273	-1.71720	1.54480
H	5.85859	-0.63799	1.81972
H	5.94016	-2.06134	0.74685
H	7.23828	-0.84614	0.71713
H	1.85923	1.11892	-2.27646
H	4.30367	1.01976	-1.98376
H	3.76799	-1.55975	1.38835
H	1.36745	-1.47151	1.08596
H	-0.24587	0.39157	-1.79685
H	-2.67171	0.93998	-0.80703
H	-3.07491	3.32955	-0.84168
H	0.71235	4.02478	1.16068
H	1.13539	1.63058	1.20756
H	-0.26623	-0.11674	2.07129
H	-1.93529	0.44291	2.02296
H	-4.42989	-0.80654	1.81004
H	-3.37329	-2.08970	2.43279
H	-4.59447	-2.48355	1.23204
H	-0.66236	-3.02982	0.96200
H	-0.47296	-2.87103	-1.67420
H	-5.11944	-1.39261	-0.78797
H	-4.39952	-1.52123	-2.32357
O	-1.44542	5.08992	0.16134
O	5.55635	-0.25321	-0.19743
O	-0.12512	-2.47764	-0.86910
O	-0.98476	-2.36127	1.58374
N	-2.03176	-1.35105	-1.55781
N	-2.89756	-1.49082	0.52234
N	-4.33084	-1.68793	-1.33491

Figure S1, Structure 10, Method: WB97XD-def2tzvp, Energy/Hartree=-1201.8249962

C	-6.04163	-1.75097	-0.17949
C	-3.67744	-1.84254	-0.05171
C	-3.52897	-0.53811	-0.50425
C	-2.25616	0.00138	-0.63440
C	-1.11606	-0.72065	-0.31377
C	-1.28575	-2.03465	0.12055
C	-2.54265	-2.58915	0.25167
C	0.25408	-0.12492	-0.51901
C	1.43718	-0.82451	0.14131
C	2.56852	0.18550	-0.12757
C	2.00204	1.43994	0.49200
C	0.47331	1.37330	0.01867
C	0.25920	2.35156	-1.09528
C	-0.50406	3.43658	-1.00269
C	-1.24615	3.75932	0.23195
C	-1.12717	2.78091	1.32426
C	-0.37478	1.68949	1.21270
C	3.28717	-1.89831	-0.02071
C	5.03645	-0.14117	0.18699
H	-6.13846	-0.86009	0.44941
H	-6.07498	-1.45166	-1.23210
H	-6.87039	-2.42490	0.02607
H	-0.41967	-2.64312	0.34293
H	-2.66736	-3.60991	0.58908
H	-4.38521	0.06942	-0.75981
H	-2.16656	1.01683	-0.99932
H	0.45374	-0.10079	-1.58939
H	-0.31822	0.97122	2.02126
H	-1.70124	2.98610	2.21916
H	-0.62209	4.12595	-1.82967
H	0.78375	2.13403	-2.02041
H	2.47559	2.36274	0.15233
H	2.05888	1.39724	1.57557
H	5.71353	-0.75337	0.78384
H	5.10315	0.87558	0.57392
H	5.37260	-0.13913	-0.85699
H	3.09890	1.25130	-1.67493
H	1.45298	-1.69887	1.86102
H	5.15507	-2.65058	-0.33361
H	3.86860	-3.75375	-0.45496
O	-1.92219	4.76462	0.32718
O	-4.86855	-2.46772	0.11704
O	1.16130	-0.84370	1.54091
O	2.71708	0.38422	-1.53029
N	2.02329	-2.09325	-0.22242
N	3.67951	-0.60596	0.34451
N	4.21482	-2.88958	-0.07489

Figure S1, Structure 11, Method: WB97XD-def2tzvp, Energy/Hartree=-1201.8200429

C	5.13150	-3.07747	0.63949
C	3.65628	-1.38791	-0.12341
C	2.52543	-2.13392	0.17691
C	1.26015	-1.61221	-0.07220
C	1.09449	-0.35274	-0.62207
C	2.24653	0.37310	-0.93652
C	3.50717	-0.12274	-0.68779
C	-0.23774	0.27493	-0.95053
C	-1.53931	-0.53668	-0.90627
C	-2.07327	-0.30757	0.51279
C	-2.07544	1.19838	0.59917
C	-0.66495	1.55731	-0.05393
C	0.33175	1.83465	1.02949
C	0.90659	3.01653	1.23076
C	0.64278	4.17153	0.35411
C	-0.27638	3.92424	-0.77078
C	-0.86015	2.74255	-0.94862
C	-2.74140	-2.22223	-0.35584
C	-3.95654	-1.47317	1.67504
H	4.68357	-3.15842	1.63533
H	4.72123	-3.86394	-0.00226
H	6.20836	-3.20822	0.72073
H	2.14929	1.35658	-1.38329
H	4.39259	0.45019	-0.92987
H	2.60635	-3.12413	0.60230
H	0.39528	-2.21315	0.16321
H	-0.15395	0.64011	-1.97258
H	-1.54138	2.58117	-1.77754
H	-0.45475	4.75623	-1.44067
H	1.61401	3.17523	2.03521
H	0.58600	0.99794	1.66889
H	-2.14297	1.59045	1.61562
H	-2.88447	1.61758	0.00804
H	-4.91987	-1.92812	1.43935
H	-4.17237	-0.52738	2.17187
H	-3.41278	-2.12311	2.36810
H	-1.24350	-0.33589	2.27953
H	-2.71588	-0.50674	-2.42721
H	-4.24485	-3.53855	0.08056
H	-3.06997	-4.07764	-1.03764
O	1.15725	5.25586	0.54259
O	4.93057	-1.80132	0.08491
O	-2.40385	0.16328	-1.81544
O	-1.15062	-0.82337	1.45991
N	-1.73743	-1.94787	-1.12801
N	-3.21703	-1.19400	0.46463
N	-3.32148	-3.44605	-0.29838

Scheme-3, Structure 19, Method: WB97XD-def2tzvp, Energy/Hartree=-1050.1546253

O	-0.82296	5.09817	0.04879
O	-5.07016	-1.40538	-0.17428
N	1.70877	-2.21226	-0.58258
N	3.58022	-1.27977	0.24363
N	3.60549	-3.60682	-0.04478
C	3.01269	-2.49028	-0.12539
C	2.76786	-0.19953	-0.27355
H	3.14636	0.11699	-1.26002
C	2.41965	1.01333	0.57959
H	3.07333	1.87338	0.43652
H	2.43716	0.72452	1.63192
C	0.93680	1.32876	0.16522
C	0.39956	-0.08357	-0.16085
C	1.44508	-0.87545	-0.39298
C	0.20483	1.94431	1.31556
H	0.14968	1.33645	2.21353
C	-0.35191	3.15112	1.28881
H	-0.86521	3.56462	2.14786
C	-0.31595	3.99551	0.07963
C	0.36493	3.41614	-1.09427
H	0.37820	4.02375	-1.99067
C	0.92527	2.21061	-1.05113
H	1.41179	1.80610	-1.93365
H	3.01824	-4.37298	-0.34990
C	5.01397	-1.14968	0.31089
H	5.43038	-2.06158	0.73159
H	5.27131	-0.30721	0.95345
H	5.45542	-0.98868	-0.68081
C	-1.02446	-0.44313	-0.15252
C	-1.99592	0.39826	-0.70378
H	-1.69832	1.33679	-1.15336
C	-3.32951	0.05343	-0.69542
H	-4.07628	0.70804	-1.12511
C	-3.74270	-1.15252	-0.13129
C	-2.79615	-1.99825	0.43417
H	-3.08454	-2.93043	0.89839
C	-1.45649	-1.63318	0.42192
H	-0.73869	-2.28367	0.90845
C	-5.54031	-2.60684	0.38853
H	-5.10784	-3.47963	-0.11091
H	-6.61783	-2.60908	0.24235
H	-5.32293	-2.65967	1.46000
H	1.00001	-2.92218	-0.54837

Scheme-3, Structure 29, Method: WB97XD-def2tzvp, Energy/Hartree=-1050.1515407

O	2.28624	4.16260	-0.74079
O	4.63858	-2.02482	0.01405
N	-2.27866	-1.91103	0.64845
N	-3.82667	-0.60287	-0.23948
N	-4.41471	-2.86386	-0.04151
C	-3.57371	-1.91828	0.12759
C	-2.66359	0.12880	-0.08517
C	-2.30147	1.56911	-0.07744
H	-2.81798	2.10778	0.72362
H	-2.49805	2.10460	-1.00851
C	-0.75535	1.49249	0.20872
C	-0.53022	0.07976	0.93250
C	-1.72349	-0.65407	0.43535
C	-0.03760	1.50420	-1.10609
H	-0.35106	0.74834	-1.81869
C	0.92470	2.36364	-1.42299
H	1.41863	2.33980	-2.38613
C	1.38440	3.39496	-0.47591
C	0.67087	3.44814	0.81623
H	0.98172	4.22375	1.50509
C	-0.30438	2.59427	1.11022
H	-0.81382	2.66213	2.06734
H	-4.06464	-3.74849	0.30360
C	-5.02325	-0.22226	-0.94233
H	-5.01212	-0.58008	-1.97539
H	-5.10727	0.86371	-0.93583
H	-5.88717	-0.65795	-0.44314
C	0.82142	-0.53651	0.68189
C	1.91992	-0.14805	1.44754
H	1.78555	0.57237	2.24625
C	3.17798	-0.65372	1.20219
H	4.03048	-0.34665	1.79342
C	3.37263	-1.58032	0.17862
C	2.28768	-1.98763	-0.58796
H	2.40516	-2.70676	-1.38579
C	1.02876	-1.45983	-0.32950
H	0.19372	-1.77294	-0.94651
C	4.89441	-2.94660	-1.01868
H	4.33839	-3.87801	-0.87185
H	5.96049	-3.15726	-0.97994
H	4.64576	-2.52850	-1.99911
H	-1.75807	-2.75922	0.77222
H	-0.62616	0.24690	2.01247

Scheme-3, Structure 31, Method: WB97XD-def2tzvp, Energy/Hartree=-1050.1645288

O	2.26284	4.17429	-0.84647
O	4.63250	-2.03011	-0.02069
N	-2.22096	-1.92423	0.60962
N	-3.77163	-0.57700	-0.28318
N	-4.28895	-2.91024	-0.10182
C	-3.43257	-1.84092	0.11679
C	-2.65720	0.18877	-0.02062
C	-2.27409	1.62130	-0.01381
H	-2.76180	2.17792	0.79308
H	-2.45747	2.16642	-0.94242
C	-0.72342	1.49775	0.25407
C	-0.52057	0.08151	0.97817
C	-1.74215	-0.64158	0.50856
C	-0.03385	1.47644	-1.07509
H	-0.35527	0.69607	-1.75738
C	0.91370	2.33416	-1.43882
H	1.38675	2.28574	-2.41149
C	1.38689	3.39440	-0.53207
C	0.72270	3.46077	0.78529
H	1.05697	4.24565	1.45245
C	-0.23630	2.60582	1.12685
H	-0.70863	2.68289	2.10231
C	-4.99867	-0.18091	-0.92751
H	-5.31846	-0.96281	-1.61695
H	-4.82604	0.73417	-1.49087
H	-5.79515	0.00282	-0.20236
C	0.82385	-0.54373	0.71295
C	1.95116	-0.08260	1.39214
H	1.84361	0.69606	2.13856
C	3.20543	-0.58849	1.12743
H	4.07912	-0.22472	1.65195
C	3.36591	-1.59005	0.17178
C	2.25228	-2.07349	-0.50262
H	2.34212	-2.85971	-1.23847
C	0.99662	-1.54623	-0.22618
H	0.13487	-1.94088	-0.74968
C	4.84874	-3.03548	-0.98002
H	4.31496	-3.95655	-0.72446
H	5.91870	-3.23108	-0.97964
H	4.54348	-2.70910	-1.97943
H	-0.60041	0.25621	2.05732
H	-5.21178	-2.79177	0.28936
H	-3.86834	-3.76505	0.22921

Scheme-3, Structure 32, Method: WB97XD-def2tzvp, Energy/Hartree=-1050.1561215

O	-0.26138	5.26120	-0.01527
O	-5.19538	-1.15320	-0.05869
N	1.49680	-2.36056	-0.32978
N	3.50675	-1.41781	0.23226
N	3.30949	-3.78000	0.05327
C	2.74669	-2.54836	-0.02040
C	2.69500	-0.32485	-0.28546
H	3.03854	-0.08063	-1.30428
C	2.43183	0.93710	0.51211
H	3.10292	1.77074	0.30357
H	2.47357	0.70330	1.57723
C	0.94232	1.28143	0.12800
C	0.34014	-0.12180	-0.10462
C	1.34966	-0.98212	-0.30097
C	0.32554	2.00816	1.28284
H	0.22235	1.43053	2.19624
C	-0.04321	3.28555	1.25006
H	-0.45046	3.78703	2.11909
C	0.05323	4.08820	0.01649
C	0.53761	3.37646	-1.18137
H	0.55202	3.94345	-2.10397
C	0.93256	2.10703	-1.12726
H	1.28085	1.60711	-2.02598
C	4.93668	-1.38796	0.02741
H	5.43595	-2.15025	0.62564
H	5.31531	-0.42312	0.36208
H	5.21225	-1.52839	-1.02499
C	-1.09648	-0.40783	-0.08285
C	-2.05019	0.59946	-0.26587
H	-1.73362	1.61939	-0.43438
C	-3.40143	0.32459	-0.25414
H	-4.12973	1.11159	-0.40001
C	-3.85121	-0.97758	-0.05852
C	-2.92185	-1.99652	0.11843
H	-3.23707	-3.01937	0.26889
C	-1.56597	-1.70756	0.10366
H	-0.85357	-2.51114	0.23361
C	-5.69579	-2.45370	0.12825
H	-5.36066	-3.13148	-0.66361
H	-6.77983	-2.37090	0.09200
H	-5.39927	-2.86321	1.09943
H	2.65385	-4.54161	0.04747
H	4.12848	-3.90679	0.61865

Scheme-3, Structure 19, Method: WB97XD-def2tzvp/SMD:methanol, Energy/Hartree=-1050.1926412

O	-0.78582	5.09468	0.00598
O	-5.10472	-1.35007	-0.15527
N	1.72007	-2.23804	-0.50975
N	3.60067	-1.27703	0.24932
N	3.63544	-3.61045	-0.04509
C	3.03401	-2.48708	-0.10100
C	2.75805	-0.21002	-0.26095
H	3.10990	0.09604	-1.25745
C	2.41935	1.00897	0.58200
H	3.06995	1.86677	0.41746
H	2.43827	0.74067	1.63952
C	0.93160	1.31140	0.16791
C	0.39146	-0.10900	-0.12978
C	1.44254	-0.90266	-0.34703
C	0.21743	1.94856	1.31296
H	0.16805	1.36126	2.22452
C	-0.32237	3.16573	1.27066
H	-0.81773	3.59954	2.13132
C	-0.28232	3.97398	0.04955
C	0.37744	3.38480	-1.11639
H	0.39574	3.97682	-2.02386
C	0.92332	2.16997	-1.06013
H	1.40441	1.75148	-1.93852
H	3.01343	-4.36224	-0.32392
C	5.03566	-1.11545	0.24007
H	5.50399	-1.91790	0.80669
H	5.28522	-0.16705	0.71537
H	5.44111	-1.11834	-0.77891
C	-1.03639	-0.45417	-0.12483
C	-2.00369	0.44498	-0.58751
H	-1.70284	1.41315	-0.96723
C	-3.34527	0.12253	-0.58510
H	-4.08269	0.82705	-0.95039
C	-3.76920	-1.11912	-0.11576
C	-2.82832	-2.02575	0.35845
H	-3.12479	-2.99143	0.74314
C	-1.48249	-1.68424	0.35334
H	-0.77437	-2.39680	0.75887
C	-5.58466	-2.59785	0.31588
H	-5.17373	-3.42643	-0.26726
H	-6.66509	-2.57070	0.18962
H	-5.34800	-2.73979	1.37383
H	1.03326	-2.97374	-0.49394

Scheme-3, Structure 29, Method: WB97XD-def2tzvp/SMD:methanol, Energy/Hartree=-1050.1910544

O	2.32656	4.10013	-0.73710
O	4.65416	-1.99006	0.02035
N	-2.29509	-1.91370	0.60277
N	-3.83311	-0.59668	-0.26104
N	-4.43695	-2.86329	-0.02976
C	-3.58490	-1.90195	0.11006
C	-2.67296	0.13839	-0.08568
C	-2.30656	1.57669	-0.07852
H	-2.81380	2.11559	0.72660
H	-2.50483	2.10940	-1.01015
C	-0.76001	1.48580	0.20361
C	-0.53899	0.06914	0.92814
C	-1.73738	-0.65554	0.42669
C	-0.04961	1.48914	-1.11085
H	-0.37705	0.74767	-1.83211
C	0.92933	2.33565	-1.42465
H	1.41796	2.30781	-2.39141
C	1.39590	3.34306	-0.47087
C	0.69382	3.41586	0.81295
H	1.01519	4.18313	1.50736
C	-0.29720	2.57541	1.10685
H	-0.80480	2.64785	2.06341
H	-4.03867	-3.73178	0.31098
C	-5.05134	-0.17315	-0.90314
H	-5.16704	-0.64486	-1.88227
H	-5.02068	0.90717	-1.03143
H	-5.91485	-0.43074	-0.28865
C	0.81618	-0.54060	0.67978
C	1.90545	-0.14740	1.45685
H	1.75781	0.55980	2.26544
C	3.17198	-0.64014	1.21487
H	4.01459	-0.32929	1.82054
C	3.37984	-1.55653	0.18477
C	2.30416	-1.97129	-0.59144
H	2.43360	-2.68683	-1.39122
C	1.03835	-1.45689	-0.33715
H	0.21195	-1.78217	-0.95900
C	4.91728	-2.92299	-1.01393
H	4.36520	-3.85382	-0.85798
H	5.98526	-3.12671	-0.96987
H	4.66873	-2.50805	-1.99449
H	-1.79980	-2.76426	0.80895
H	-0.63850	0.23933	2.00552

Scheme-3, Structure 31, Method: WB97XD-def2tzvp/SMD:methanol, Energy/Hartree=-1050.2048574

O	2.41796	4.03882	-0.76842
O	4.62056	-2.05675	0.02022
N	-2.26084	-1.89989	0.62203
N	-3.79774	-0.54578	-0.28340
N	-4.35597	-2.85349	-0.04733
C	-3.48364	-1.80451	0.13540
C	-2.66467	0.20490	-0.05861
C	-2.27202	1.63486	-0.07455
H	-2.76315	2.20445	0.71939
H	-2.44984	2.15895	-1.01549
C	-0.72615	1.50739	0.21202
C	-0.53479	0.09203	0.95117
C	-1.75923	-0.62411	0.48255
C	-0.02058	1.48002	-1.10370
H	-0.36788	0.73838	-1.81567
C	0.97697	2.29980	-1.43124
H	1.46142	2.24927	-2.39922
C	1.47063	3.30606	-0.49059
C	0.77549	3.40924	0.79454
H	1.11812	4.17554	1.47990
C	-0.23545	2.59646	1.09987
H	-0.73808	2.69236	2.05711
C	-5.03245	-0.13513	-0.91339
H	-5.19352	-0.68536	-1.84217
H	-4.97174	0.92727	-1.13833
H	-5.88084	-0.30195	-0.24760
C	0.80777	-0.54310	0.69912
C	1.90695	-0.17424	1.47426
H	1.77448	0.53121	2.28711
C	3.16479	-0.68756	1.22667
H	4.01403	-0.39487	1.83226
C	3.35388	-1.60118	0.19119
C	2.26862	-1.99252	-0.58402
H	2.38339	-2.70616	-1.38780
C	1.01247	-1.45835	-0.32311
H	0.17787	-1.76809	-0.94090
C	4.86262	-2.98967	-1.01886
H	4.29244	-3.91011	-0.86630
H	5.92647	-3.21492	-0.97849
H	4.62022	-2.56540	-1.99705
H	-0.61369	0.28398	2.02627
H	-5.33141	-2.62533	0.08363
H	-4.09169	-3.67398	0.47761

Scheme-3, Structure 32, Method: WB97XD-def2tzvp/SMD:methanol, Energy/Hartree=-1050.1970187

O	-0.58164	5.18537	-0.02640
O	-5.15530	-1.29348	-0.09495
N	1.58250	-2.30332	-0.37456
N	3.55723	-1.32073	0.22918
N	3.45003	-3.66336	-0.03014
C	2.85715	-2.46570	-0.06492
C	2.71870	-0.23947	-0.26419
H	3.05660	0.04243	-1.27236
C	2.41449	0.99586	0.56045
H	3.07325	1.84424	0.37717
H	2.43685	0.74631	1.62260
C	0.92425	1.31161	0.15152
C	0.35990	-0.10462	-0.11189
C	1.39738	-0.93859	-0.30488
C	0.25774	1.99808	1.29741
H	0.19038	1.42442	2.21651
C	-0.20590	3.24676	1.25259
H	-0.65761	3.71833	2.11745
C	-0.14783	4.03515	0.01994
C	0.43940	3.38982	-1.15437
H	0.46049	3.96409	-2.07322
C	0.92390	2.14873	-1.09065
H	1.35804	1.69205	-1.97453
C	4.99723	-1.23106	0.10681
H	5.48636	-1.97736	0.73270
H	5.31236	-0.25135	0.46278
H	5.33068	-1.35464	-0.92954
C	-1.06983	-0.42966	-0.10235
C	-2.04967	0.53034	-0.38594
H	-1.76292	1.54301	-0.63667
C	-3.39468	0.21703	-0.37580
H	-4.13737	0.97317	-0.60078
C	-3.81410	-1.07759	-0.08207
C	-2.86253	-2.05112	0.19944
H	-3.15237	-3.06591	0.43436
C	-1.51439	-1.72109	0.18781
H	-0.78991	-2.49196	0.41544
C	-5.62608	-2.59701	0.19918
H	-5.25094	-3.32737	-0.52307
H	-6.71100	-2.54786	0.12966
H	-5.34291	-2.90281	1.21021
H	2.86862	-4.48093	-0.10109
H	4.36785	-3.77579	0.36319

Scheme-3, Structure 19, Method: WB97XD-def2tzvp/SMD:dichloromethane, Energy/Hartree=-1050.1953586

O	-0.72732	5.11747	0.01914
O	-5.11127	-1.32977	-0.14870
N	1.70544	-2.24684	-0.52249
N	3.58938	-1.29706	0.23189
N	3.61075	-3.63182	-0.03691
C	3.01644	-2.50626	-0.10628
C	2.75499	-0.22365	-0.27339
H	3.10941	0.08766	-1.26783
C	2.42012	0.99204	0.57668
H	3.07549	1.84801	0.42124
H	2.43642	0.71356	1.63175
C	0.93537	1.30572	0.16170
C	0.38808	-0.11105	-0.14016
C	1.43471	-0.90920	-0.36225
C	0.22368	1.94678	1.30855
H	0.16431	1.35409	2.21612
C	-0.29940	3.17053	1.27264
H	-0.79123	3.60409	2.13533
C	-0.24849	3.99409	0.05554
C	0.40311	3.39129	-1.11565
H	0.42750	3.98498	-2.02184
C	0.93286	2.17020	-1.06465
H	1.40577	1.74763	-1.94569
H	2.99024	-4.38634	-0.30961
C	5.02282	-1.14334	0.27526
H	5.46571	-1.98196	0.80844
H	5.26420	-0.22474	0.81005
H	5.45998	-1.09359	-0.72942
C	-1.04114	-0.45210	-0.13157
C	-2.00655	0.44933	-0.59421
H	-1.70102	1.41561	-0.97471
C	-3.34903	0.13282	-0.58797
H	-4.08445	0.83950	-0.95221
C	-3.77923	-1.10610	-0.11409
C	-2.83876	-2.01455	0.36016
H	-3.13851	-2.97749	0.74907
C	-1.49137	-1.67864	0.35086
H	-0.78476	-2.39179	0.75824
C	-5.59666	-2.57064	0.32609
H	-5.19487	-3.40569	-0.25521
H	-6.67747	-2.53812	0.20416
H	-5.35881	-2.71444	1.38408
H	1.01258	-2.97609	-0.51652

Scheme-3, Structure 29, Method: WB97XD-def2tzvp/SMD:dichloromethane, Energy/Hartree=-1050.1939979

O	2.23945	4.19684	-0.73827
O	4.66227	-1.99710	0.01941
N	-2.26673	-1.93354	0.59861
N	-3.81630	-0.63149	-0.26589
N	-4.40489	-2.89943	-0.03332
C	-3.55841	-1.93586	0.10699
C	-2.66540	0.11594	-0.08410
C	-2.30941	1.55715	-0.07945
H	-2.82500	2.09583	0.72077
H	-2.50722	2.08605	-1.01340
C	-0.76192	1.48140	0.20883
C	-0.52843	0.06552	0.92848
C	-1.72302	-0.66830	0.43094
C	-0.04653	1.50391	-1.10466
H	-0.35054	0.75062	-1.82392
C	0.90431	2.37989	-1.42004
H	1.39313	2.36414	-2.38685
C	1.34529	3.40964	-0.46951
C	0.64277	3.44912	0.82231
H	0.94366	4.22232	1.51923
C	-0.31998	2.57827	1.11703
H	-0.82520	2.63137	2.07649
H	-4.01022	-3.76922	0.30704
C	-5.04196	-0.21739	-0.89936
H	-5.15301	-0.67701	-1.88455
H	-5.02833	0.86522	-1.01085
H	-5.89885	-0.50039	-0.28724
C	0.82805	-0.54172	0.67720
C	1.91674	-0.15935	1.46084
H	1.76887	0.54301	2.27358
C	3.18228	-0.65384	1.21951
H	4.02372	-0.35010	1.82984
C	3.39320	-1.56252	0.18199
C	2.31688	-1.96573	-0.60100
H	2.44696	-2.67376	-1.40726
C	1.05168	-1.44994	-0.34647
H	0.22586	-1.76710	-0.97329
C	4.92877	-2.91421	-1.02402
H	4.37635	-3.84827	-0.88580
H	5.99622	-3.12062	-0.97914
H	4.68616	-2.48812	-2.00181
H	-1.76612	-2.77575	0.82243
H	-0.62430	0.23337	2.00683

Scheme-3, Structure 31, Method: WB97XD-def2tzvp/SMD:dichloromethane, Energy/Hartree=-1050.2070435

O	2.31587	4.14796	-0.78731
O	4.63119	-2.05731	0.01239
N	-2.22798	-1.92106	0.61642
N	-3.77989	-0.58195	-0.28383
N	-4.31041	-2.89807	-0.06239
C	-3.44969	-1.83986	0.12903
C	-2.65830	0.18313	-0.04769
C	-2.27733	1.61661	-0.06070
H	-2.77284	2.18202	0.73384
H	-2.45878	2.14184	-1.00038
C	-0.72860	1.50289	0.22445
C	-0.52280	0.08734	0.95556
C	-1.74340	-0.63865	0.49007
C	-0.02430	1.49383	-1.09428
H	-0.34894	0.73629	-1.80022
C	0.94095	2.34647	-1.43076
H	1.42134	2.30716	-2.40116
C	1.41051	3.37999	-0.49877
C	0.72286	3.44865	0.79950
H	1.04763	4.22285	1.48461
C	-0.25499	2.60113	1.11371
H	-0.74863	2.67604	2.07787
C	-5.02096	-0.18277	-0.90721
H	-5.21608	-0.78785	-1.79416
H	-4.94285	0.86079	-1.20331
H	-5.85738	-0.28583	-0.21335
C	0.82161	-0.54285	0.69795
C	1.92549	-0.16584	1.46276
H	1.79696	0.54767	2.26915
C	3.18242	-0.67929	1.21319
H	4.03457	-0.37953	1.81063
C	3.36894	-1.60304	0.18510
C	2.27768	-2.00202	-0.57899
H	2.38826	-2.72412	-1.37573
C	1.02184	-1.46818	-0.31552
H	0.18260	-1.78912	-0.92082
C	4.86942	-2.99644	-1.01740
H	4.30271	-3.91837	-0.85698
H	5.93371	-3.22073	-0.98167
H	4.62261	-2.58369	-2.00003
H	-0.59865	0.27368	2.03204
H	-5.28599	-2.69394	0.09955
H	-4.01682	-3.72944	0.42813

Scheme-3, Structure 32, Method: WB97XD-def2tzvp/SMD:dichloromethane, Energy/Hartree=-1050.1991657

O	-0.43281	5.22451	-0.02269
O	-5.17971	-1.22453	-0.06404
N	1.54016	-2.33370	-0.34786
N	3.53437	-1.36787	0.20450
N	3.38920	-3.71601	0.01232
C	2.81305	-2.50864	-0.04786
C	2.70532	-0.28227	-0.29184
H	3.03329	-0.01552	-1.30780
C	2.42698	0.96532	0.52332
H	3.09211	1.80510	0.32345
H	2.46359	0.72337	1.58693
C	0.93471	1.29624	0.13375
C	0.34910	-0.11425	-0.11037
C	1.37315	-0.96465	-0.30492
C	0.30141	2.00479	1.28805
H	0.22764	1.43225	2.20745
C	-0.11796	3.26842	1.25040
H	-0.54036	3.75362	2.12242
C	-0.05389	4.06280	0.01538
C	0.47984	3.38274	-1.17242
H	0.49492	3.95033	-2.09549
C	0.92437	2.12761	-1.11458
H	1.31770	1.65084	-2.00706
C	4.97535	-1.29593	0.11240
H	5.44110	-2.04553	0.75228
H	5.29561	-0.31931	0.47245
H	5.33242	-1.42758	-0.91527
C	-1.08438	-0.42031	-0.09245
C	-2.05457	0.56700	-0.30750
H	-1.75427	1.58738	-0.50411
C	-3.40362	0.27317	-0.29292
H	-4.13821	1.05067	-0.46384
C	-3.83972	-1.02968	-0.06327
C	-2.89601	-2.03006	0.14659
H	-3.19747	-3.05263	0.32688
C	-1.54333	-1.72024	0.13002
H	-0.82502	-2.51237	0.29539
C	-5.66501	-2.53257	0.16221
H	-5.31816	-3.22751	-0.60843
H	-6.75038	-2.46443	0.11904
H	-5.36807	-2.90520	1.14716
H	2.79179	-4.52378	-0.02653
H	4.30717	-3.83342	0.40237

Table S1. Crystal data and structure refinement of 71 CCDC Deposition # (2402251).

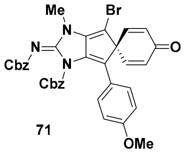

Empirical formula	$C_{35}H_{28}O_6Cl_3N_3Br_{0.971}$	
Formula weight	664.2 g/mol	
Temperature	293 K	
Wavelength	0.71073 Å	
Crystal system	Monoclinic	
Space group	P21	
Unit cell dimensions	a = 9.969(2) Å	[illegible]= 90 °
	b = 10.930(7) Å	[illegible]= 104.62(2) °
	c = 15.092(2) Å	[illegible] =90°
Volume	1590.99(1) Å^3	
Z	2	
Density (calculated)	1.3865 g/cm^3	
Absorption coefficient	1.305 mm $^{-1}$	
F(000)	680	
Crystal size	0.150 x 0.120 x 0.090 mm^3	
Crystal color, habit	Brown block	
Theta range for data collection	2.1 to 32.9°	
Index ranges	-15≤h≤15, -16≤k ≤16, -22≤l≤22	
Reflections collected	21198	
Independent reflections	6062 [R(int) = %, R(sigma) = %]	
Completeness to theta = 32.9°	99 %	
Absorption correction	multi-scan	
Max. and min. transmission	0.950 and 0.989	
Refinement method	Full-matrix least-squares on F^2	

Data / restraints / parameters	10700/0/408
Goodness-of-fit on F^2	1.34
Final R indices [I>3sigma(I)]	R1 = 4.65, wR2 = 4.11
R indices (all data)	R1 = 17.40, wR2 = 5.26
Extinction coefficient	n/a
Largest diff. peak and hole	-0.04 and + 0.06 $e^- Å^{-3}$

Table S2. Atomic Positional parameters, atomic occupation and U_{eq}($Å^2$)

Atom	Occupation	x	y	z	U_{eq}($Å^2$)
Br	0.9709(19)	0.86853(4)	0.02397(4)	0.46459(3)	0.06402(17)
O1	1	0.7705(3)	0.6254(2)	0.32571(18)	0.0589(12)
O2	1	0.6489(3)	0.6898(2)	0.63397(17)	0.0574(11)
N1	1	0.7652(3)	0.4926(2)	0.43641(19)	0.0399(11)
N2	1	0.7861(3)	0.3304(3)	0.5342(2)	0.0465(12)
O3	1	0.7853(3)	0.7009(2)	0.46782(19)	0.0703(13)
N3	1	0.6939(3)	0.5085(3)	0.58038(19)	0.0540(13)
O4	1	0.5171(3)	0.6293(3)	0.4960(2)	0.0857(14)
O5	1	0.8791(4)	0.6366(3)	0.0042(2)	0.0858(15)
C1	1	0.6002(4)	0.8626(4)	0.7191(3)	0.0494(15)
C2	1	0.8665(4)	0.2144(3)	0.3209(2)	0.0458(15)
C3	1	0.6465(4)	0.7988(3)	0.2373(3)	0.0519(17)
C4	1	0.8512(4)	0.1846(3)	0.4169(3)	0.0468(14)
C5	1	0.7748(4)	0.6188(4)	0.4142(3)	0.0435(15)
C6	1	0.8560(4)	0.4262(3)	0.2316(2)	0.0431(14)
C7	1	0.8170(4)	0.2881(3)	0.4552(3)	0.0431(15)
C8	1	0.7439(4)	0.4526(3)	0.5211(3)	0.0457(14)
C9	1	0.7871(5)	0.7486(3)	0.2880(3)	0.0625(19)
C10	1	0.6112(4)	0.6132(4)	0.5615(3)	0.0540(18)
C11	1	0.5887(4)	0.9888(4)	0.7234(3)	0.0600(18)
C12	1	0.7479(4)	0.1576(3)	0.2491(3)	0.0557(16)
C13	1	0.9830(4)	0.4727(4)	0.2256(3)	0.0586(17)
C14	1	0.7406(4)	0.4492(3)	0.1587(2)	0.0532(16)
C15	1	0.8424(4)	0.3571(3)	0.3137(2)	0.0427(14)
C16	1	0.7528(4)	0.5185(5)	0.0838(2)	0.0600(16)
C17	1	0.8105(4)	0.3902(3)	0.3913(3)	0.0395(14)
C18	1	1.0067(4)	0.1781(3)	0.3103(3)	0.0540(16)
C19	1	0.6048(5)	0.7917(3)	0.1420(3)	0.0610(19)
C20	1	0.8801(5)	0.5663(3)	0.0799(3)	0.0598(19)
O6	1	0.9208(5)	-0.0449(4)	0.1315(3)	0.135(2)
C21	1	0.5652(4)	0.8002(4)	0.6281(3)	0.0683(19)
C22	1	1.0236(5)	0.0974(4)	0.2477(3)	0.067(2)
C23	1	0.7661(5)	0.0780(3)	0.1865(3)	0.068(2)
C24	1	0.6389(4)	0.7971(4)	0.8021(3)	0.0625(18)
C25	1	0.9958(4)	0.5435(4)	0.1510(3)	0.0666(19)

C26	1	0.6512(4)	0.9837(5)	0.8887(3)	0.076(2)
C27	1	0.4772(5)	0.8384(4)	0.0947(3)	0.074(2)
C28	1	0.9044(6)	0.0384(5)	0.1840(3)	0.079(2)
C29	1	0.5604(5)	0.8553(4)	0.2843(3)	0.0628(18)
C30	1	0.6140(4)	1.0485(4)	0.8074(3)	0.072(2)
C31	1	0.4332(5)	0.9029(4)	0.2372(3)	0.071(2)
C32	1	0.6652(4)	0.8580(5)	0.8857(3)	0.072(2)
C33	1	0.7770(5)	0.2591(3)	0.6142(3)	0.0650(19)
C34	1	0.3906(5)	0.8946(4)	0.1425(3)	0.074(2)
C35	1	1.0024(6)	0.7051(5)	0.0038(4)	0.100(3)
H1c9	1	0.8458	0.7427	0.2466	0.075002
H2c9	1	0.8292	0.8029	0.3372	0.075002
H1c11	1	0.563	1.0354	0.6678	0.072034
H1c12	1	0.6548	0.1804	0.249	0.066791
H1c13	1	1.0642	0.4557	0.2739	0.070274
H1c14	1	0.6518	0.4166	0.1603	0.063827
H1c16	1	0.6724	0.5334	0.0344	0.072021
H1c18	1	1.0875	0.2143	0.3502	0.064804
H1c19	1	0.6646	0.7544	0.1089	0.073164
H1c21	1	0.4687	0.7786	0.6118	0.082013
H2c21	1	0.5849	0.854	0.5828	0.082013
H1c22	1	1.1158	0.0773	0.244	0.080817
H1c23	1	0.6869	0.0462	0.1424	0.081926
H1c24	1	0.6473	0.7096	0.8009	0.075005
H1c25	1	1.0844	0.5761	0.1491	0.079882
H1c26	1	0.6671	1.0255	0.9464	0.091765
H1c27	1	0.4484	0.8321	0.0292	0.088908
H1c29	1	0.589	0.8615	0.3498	0.07537
H1c30	1	0.6056	1.1359	0.809	0.086469
H1c31	1	0.3745	0.9417	0.2703	0.085744
H1c32	1	0.6931	0.8124	0.9418	0.086378
H1c33	1	0.7827	0.313	0.6652	0.077987
H2c33	1	0.6903	0.216	0.6011	0.077987
H3c33	1	0.852	0.2015	0.6288	0.077987
H1c34	1	0.3024	0.9271	0.1099	0.088425
H1c35	1	0.9866	0.7528	-0.0513	0.120543
H2c35	1	1.0248	0.7584	0.056	0.120543
H3c35	1	1.078	0.6496	0.0063	0.120543

Table S3. Anisotropic Displacement Parameters (in Å²)

Atom	U_{11}	U_{22}	U_{33}	U_{12}	U_{13}	U_{23}
Br	0.0810(3)	0.0375(2)	0.0721(3)	0.0097(3)	0.0166(2)	0.0045(3)
O1	0.093(2)	0.0372(16)	0.0487(18)	0.0013(15)	0.0227(16)	0.0005(13)
O2	0.0607(18)	0.0593(17)	0.0509(17)	0.0166(14)	0.0117(13)	-0.0138(14)
N1	0.0521(19)	0.035(2)	0.0350(17)	0.0058(13)	0.0151(13)	-0.0034(12)
N2	0.061(2)	0.0411(18)	0.0379(19)	0.0011(15)	0.0131(15)	-0.0031(15)
O3	0.121(3)	0.0389(16)	0.049(2)	0.0036(17)	0.0182(17)	-0.0119(14)
N3	0.067(2)	0.051(2)	0.0470(18)	0.009(2)	0.0201(15)	-0.0018(18)
O4	0.086(2)	0.095(3)	0.061(2)	0.023(2)	-0.0101(18)	-0.0222(18)
O5	0.116(3)	0.084(2)	0.063(2)	0.012(2)	0.0331(19)	0.0208(18)
C1	0.050(2)	0.051(2)	0.048(2)	0.0115(19)	0.0133(19)	-0.002(2)
C2	0.047(2)	0.037(2)	0.053(3)	0.0022(18)	0.0108(19)	-0.0060(18)
C3	0.074(3)	0.0281(19)	0.064(3)	0.000(2)	0.036(2)	0.0046(18)
C4	0.052(2)	0.0310(19)	0.054(2)	0.0040(17)	0.0090(18)	0.0006(17)
C5	0.048(3)	0.042(2)	0.040(3)	0.001(2)	0.010(2)	0.000(2)
C6	0.054(3)	0.039(2)	0.036(2)	0.0051(18)	0.0105(19)	-0.0071(17)
C7	0.039(2)	0.039(2)	0.047(3)	-0.0016(18)	0.0031(18)	0.0007(19)
C8	0.049(2)	0.040(2)	0.045(2)	0.0001(18)	0.0067(18)	-0.0058(18)
C9	0.087(3)	0.042(2)	0.070(3)	-0.005(2)	0.042(3)	0.003(2)
C10	0.062(3)	0.061(3)	0.044(3)	0.002(2)	0.022(2)	-0.004(2)
C11	0.055(3)	0.057(3)	0.067(3)	0.009(2)	0.014(2)	0.006(2)
C12	0.058(3)	0.043(2)	0.062(3)	-0.002(2)	0.006(2)	-0.005(2)
C13	0.048(3)	0.073(3)	0.056(3)	0.010(2)	0.017(2)	0.012(2)
C14	0.054(3)	0.051(2)	0.048(3)	-0.002(2)	0.001(2)	-0.011(2)
C15	0.042(2)	0.038(2)	0.044(2)	0.0062(18)	0.0047(18)	-0.0010(19)
C16	0.077(3)	0.053(2)	0.044(2)	0.004(3)	0.0038(19)	-0.010(3)
C17	0.036(2)	0.036(2)	0.045(2)	0.0009(16)	0.0069(18)	-0.0060(18)
C18	0.057(3)	0.050(2)	0.055(3)	0.012(2)	0.014(2)	0.000(2)
C19	0.093(4)	0.044(2)	0.055(3)	0.012(2)	0.035(3)	0.006(2)
C20	0.084(3)	0.058(3)	0.045(3)	0.015(2)	0.030(3)	0.001(2)
O6	0.195(4)	0.098(3)	0.138(4)	-0.019(3)	0.091(3)	-0.065(3)
C21	0.076(3)	0.061(3)	0.063(3)	0.023(3)	0.008(2)	-0.009(2)
C22	0.079(3)	0.061(3)	0.070(3)	0.020(3)	0.033(3)	0.003(2)
C23	0.100(4)	0.051(3)	0.051(3)	-0.019(2)	0.014(3)	-0.010(2)
C24	0.076(3)	0.053(3)	0.063(3)	0.014(2)	0.026(2)	0.006(2)
C25	0.057(3)	0.076(3)	0.075(3)	0.007(3)	0.031(2)	0.009(3)
C26	0.068(3)	0.101(5)	0.061(3)	0.010(3)	0.017(2)	-0.027(3)
C27	0.105(4)	0.063(3)	0.057(3)	0.018(3)	0.026(3)	0.017(2)
C28	0.124(4)	0.054(3)	0.071(3)	-0.005(4)	0.046(3)	-0.012(3)
C29	0.092(3)	0.050(2)	0.055(3)	0.002(2)	0.034(3)	0.001(2)
C30	0.073(3)	0.057(3)	0.086(4)	0.006(2)	0.019(3)	-0.015(3)
C31	0.097(4)	0.063(3)	0.070(3)	0.020(3)	0.048(3)	0.011(2)
C32	0.080(3)	0.092(4)	0.049(3)	0.022(3)	0.025(2)	0.009(3)
C33	0.099(4)	0.052(3)	0.044(3)	0.001(2)	0.019(2)	0.006(2)
C34	0.095(4)	0.059(3)	0.073(3)	0.013(3)	0.031(3)	0.018(2)
C35	0.130(5)	0.097(4)	0.095(4)	0.010(4)	0.066(3)	0.028(3)

Table S4. Main atomic bonds distances and angles (in Å and˚)

Br-N1	3.594(3)
Br-C4	1.889(3)
O1-C5	1.327(6)
O1-C9	1.487(4)
O2-C10	1.353(5)
O2-C21	1.457(5)
N1-C5	1.428(5)
N1-C8	1.417(6)
N1-C17	1.441(5)
N2-C7	1.384(6)
N2-C8	1.399(5)
N2-C33	1.458(5)
O3-C5	1.195(5)
N3-C8	1.284(6)
N3-C10	1.397(5)
O4-C10	1.191(5)
O5-C20	1.375(5)
O5-C35	1.441(7)
C1-C11	1.387(6)
C1-C21	1.493(6)
C1-C24	1.409(6)
C2-C4	1.530(6)
C2-C12	1.519(5)
C2-C18	1.500(6)
C3-C9	1.519(6)
C3-C19	1.395(6)
C3-C29	1.389(7)
C4-C7	1.353(5)
C6-C13	1.388(6)
C6-C14	1.399(4)
C6-C15	1.486(5)
C7-C17	1.466(5)
C9-H1c9	0.96
C9-H2c9	0.9597
C11-C30	1.391(6)
C11-H1c11	0.9599
C12-C23	1.330(6)
C12-H1c12	0.9601
C13-C25	1.398(7)
C13-H1c13	0.9599
C14-C16	1.391(5)
C14-H1c14	0.9598
C15-C17	1.339(6)
C16-C20	1.387(7)
C16-H1c16	0.9602
C18-C22	1.334(6)
C18-H1c18	0.9601
C19-C27	1.389(6)
C19-H1c19	0.9604
C20-C25	1.385(6)

O6-C28	1.245(7)
C21-H1c21	0.9604
C21-H2c21	0.9598
C22-C28	1.474(7)
C22-H1c22	0.9606
C23-C28	1.455(8)
C23-H1c23	0.9595
C24-C32	1.391(6)
C24-H1c24	0.9606
C25-H1c25	0.9598
C26-C30	1.384(6)
C26-C32	1.383(8)
C26-H1c26	0.9601
C27-C34	1.399(7)
C27-H1c27	0.9593
C29-C31	1.388(6)
C29-H1c29	0.9598
C30-H1c30	0.9603
C31-C34	1.387(6)
C31-H1c31	0.9592
C32-H1c32	0.9605
C33-H1c33	0.9593
C33-H2c33	0.9601
C33-H3c33	0.9605
C34-H1c34	0.9601
C35-H1c35	0.9604
C35-H2c35	0.9594
C35-H3c35	0.9602
N1-Br-C4	103.44(12)
C5-O1-C9	117.1(3)
C10-O2-C21	115.2(3)
Br-N1-C5	83.34(19)
Br-N1-C8	91.31(19)
Br-N1-C17	82.16(19)
C5-N1-C8	122.9(3)
C5-N1-C17	126.3(3)
C8-N1-C17	108.8(3)
C7-N2-C8	108.8(3)
C7-N2-C33	127.5(3)
C8-N2-C33	123.1(3)
C8-N3-C10	124.1(3)
C20-O5-C35	117.9(4)
C11-C1-C21	119.6(4)
C11-C1-C24	118.1(4)
C21-C1-C24	122.2(4)
C4-C2-C12	110.0(3)
C4-C2-C18	111.3(3)
C12-C2-C18	113.2(3)
C9-C3-C19	119.7(4)
C9-C3-C29	120.9(4)
C19-C3-C29	119.3(4)

Br-C4-C2	122.5(3)
Br-C4-C7	128.5(4)
C2-C4-C7	108.8(3)
O1-C5-N1	107.5(3)
O1-C5-O3	128.0(4)
N1-C5-O3	124.5(4)
C13-C6-C14	117.7(3)
C13-C6-C15	121.2(3)
C14-C6-C15	121.1(3)
N2-C7-C4	141.5(4)
N2-C7-C17	109.3(3)
C4-C7-C17	109.2(4)
N1-C8-N2	108.3(3)
N1-C8-N3	130.9(3)
N2-C8-N3	120.8(4)
O1-C9-C3	110.0(3)
O1-C9-H1c9	109.48
O1-C9-H2c9	109.48
C3-C9-H1c9	109.47
C3-C9-H2c9	109.45
H1c9-C9-H2c9	108.95
O2-C10-N3	108.2(3)
O2-C10-O4	125.4(4)
N3-C10-O4	126.2(4)
C1-C11-C30	120.7(4)
C1-C11-H1c11	119.57
C30-C11-H1c11	119.68
C2-C12-C23	123.5(4)
C2-C12-H1c12	118.2
C23-C12-H1c12	118.28
C6-C13-C25	121.7(3)
C6-C13-H1c13	119.17
C25-C13-H1c13	119.11
C6-C14-C16	120.9(4)
C6-C14-H1c14	119.58
C16-C14-H1c14	119.55
C6-C15-C17	133.2(3)
C14-C16-C20	120.6(3)
C14-C16-H1c16	119.74
C20-C16-H1c16	119.69
N1-C17-C7	104.3(4)
N1-C17-C15	142.7(3)
C7-C17-C15	112.9(3)
C2-C18-C22	122.6(3)
C2-C18-H1c18	118.71
C22-C18-H1c18	118.69
C3-C19-C27	120.3(4)
C3-C19-H1c19	119.86
C27-C19-H1c19	119.83
O5-C20-C16	115.6(4)
O5-C20-C25	125.0(4)

C16-C20-C25	119.4(4)
O2-C21-C1	109.0(3)
O2-C21-H1c21	109.48
O2-C21-H2c21	109.51
C1-C21-H1c21	109.45
C1-C21-H2c21	109.46
H1c21-C21-H2c21	109.94
C18-C22-C28	121.7(5)
C18-C22-H1c22	119.16
C28-C22-H1c22	119.14
C12-C23-C28	120.9(4)
C12-C23-H1c23	119.52
C28-C23-H1c23	119.56
C1-C24-C32	120.6(4)
C1-C24-H1c24	119.63
C32-C24-H1c24	119.76
C13-C25-C20	119.7(4)
C13-C25-H1c25	120.17
C20-C25-H1c25	120.12
C30-C26-C32	119.1(4)
C30-C26-H1c26	120.42
C32-C26-H1c26	120.49
C19-C27-C34	120.0(4)
C19-C27-H1c27	119.99
C34-C27-H1c27	119.96
O6-C28-C22	121.4(5)
O6-C28-C23	120.8(5)
C22-C28-C23	117.8(4)
C3-C29-C31	120.5(4)
C3-C29-H1c29	119.78
C31-C29-H1c29	119.72
C11-C30-C26	120.9(4)
C11-C30-H1c30	119.55
C26-C30-H1c30	119.51
C29-C31-C34	120.3(5)
C29-C31-H1c31	119.85
C34-C31-H1c31	119.84
C24-C32-C26	120.5(4)
C24-C32-H1c32	119.79
C26-C32-H1c32	119.71
N2-C33-H1c33	109.51
N2-C33-H2c33	109.45
N2-C33-H3c33	109.44
H1c33-C33-H2c33	109.52
H1c33-C33-H3c33	109.49
H2c33-C33-H3c33	109.42
C27-C34-C31	119.5(4)
C27-C34-H1c34	120.21
C31-C34-H1c34	120.32
O5-C35-H1c35	109.44
O5-C35-H2c35	109.5

O5-C35-H3c35	109.46
H1c35-C35-H2c35	109.49
H1c35-C35-H3c35	109.42
H2c35-C35-H3c35	109.51

^{1}H and ^{13}C NMR of 22 in $CDCl_3$:

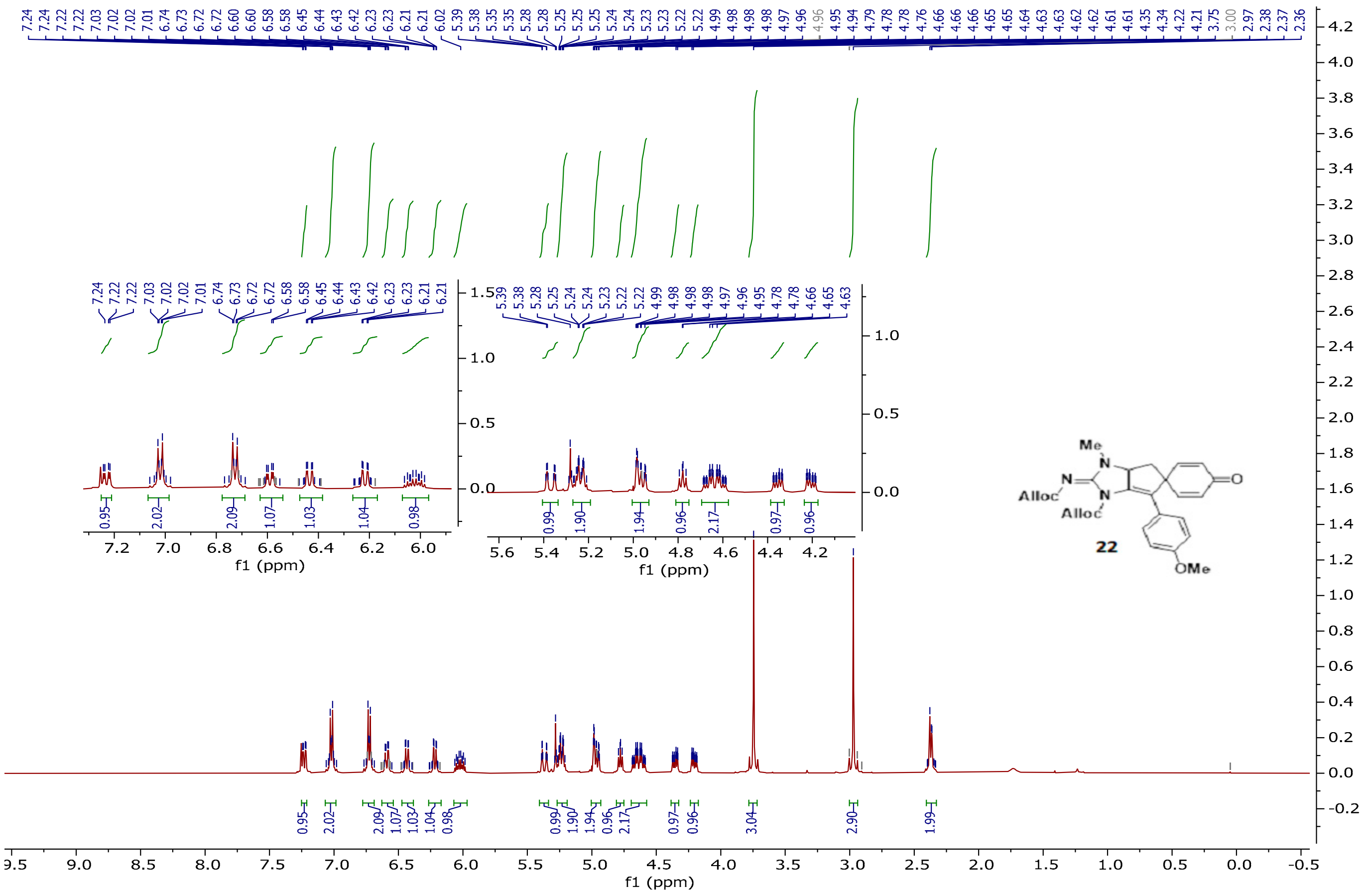

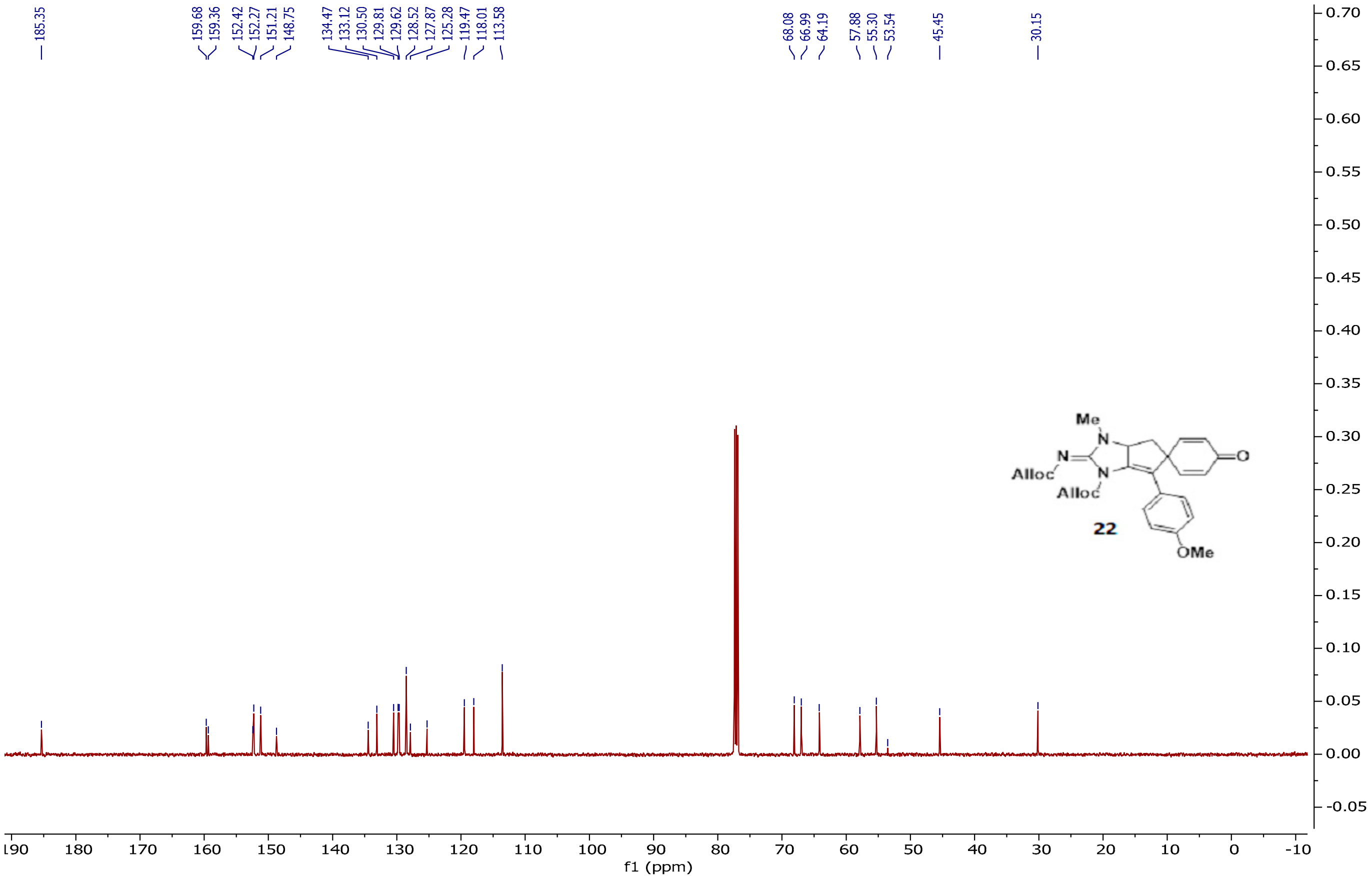

^{1}H and ^{13}C NMR of 35 in $CDCl_3$:

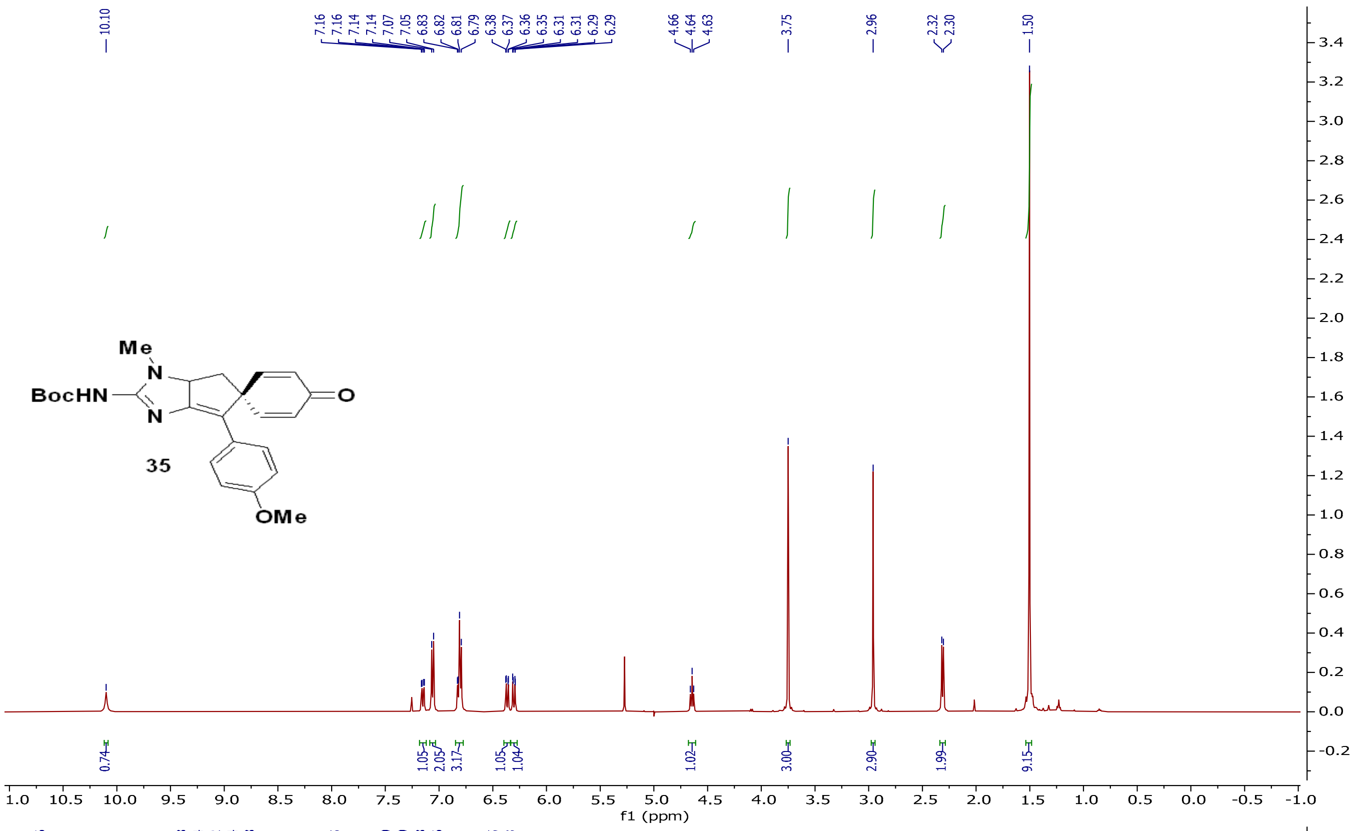

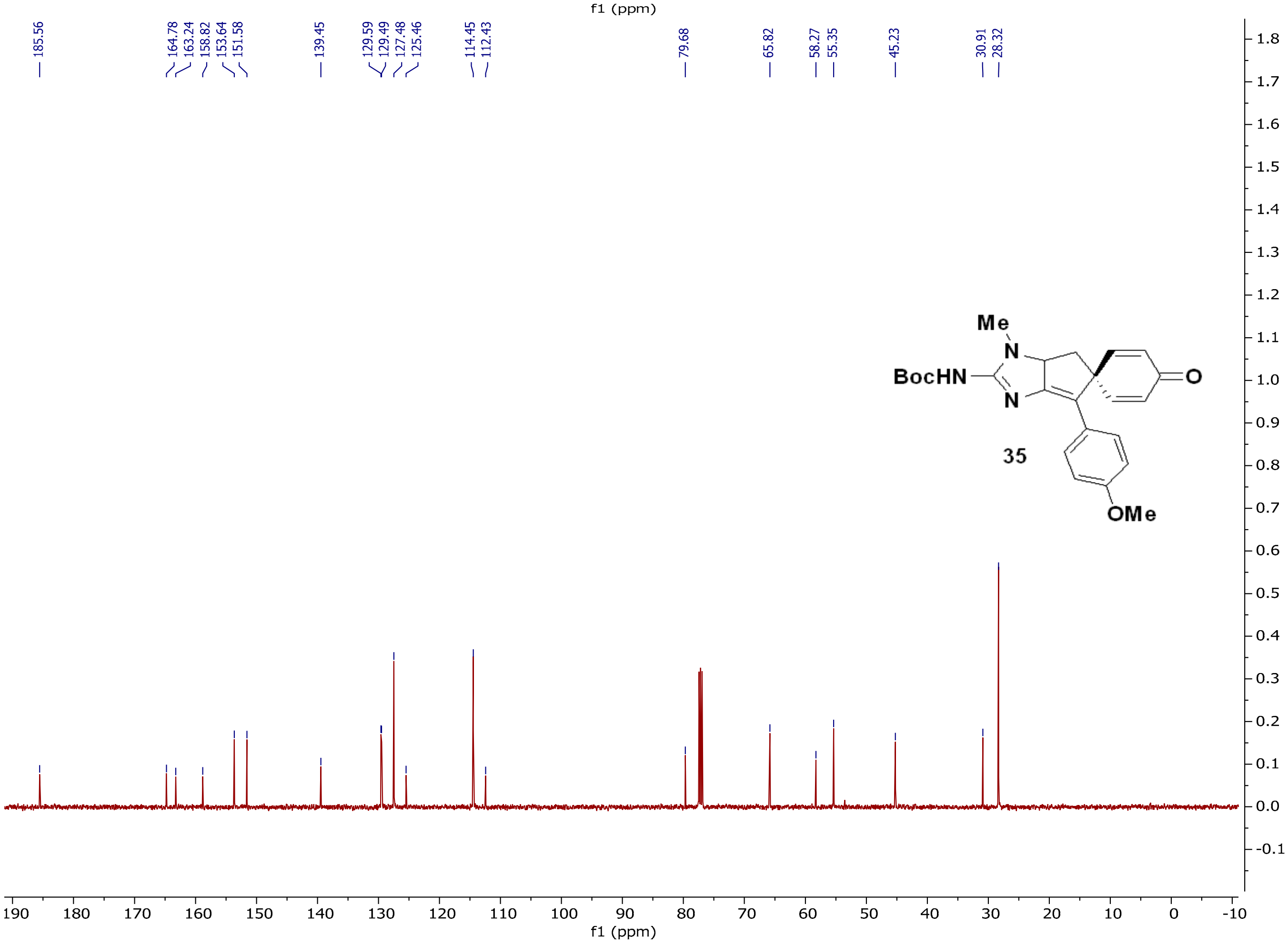

^{1}H and ^{13}C NMR of 38 in $CDCl_3$:

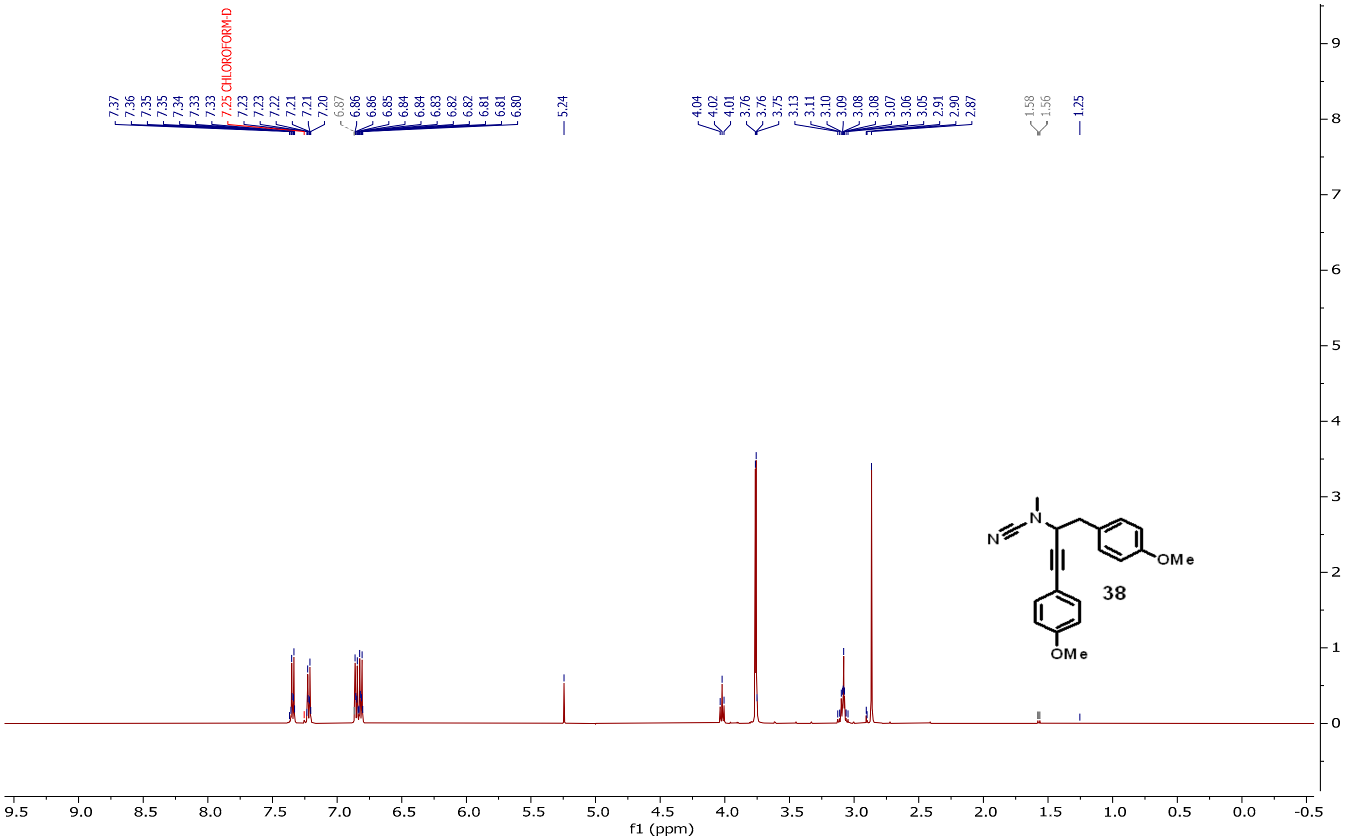

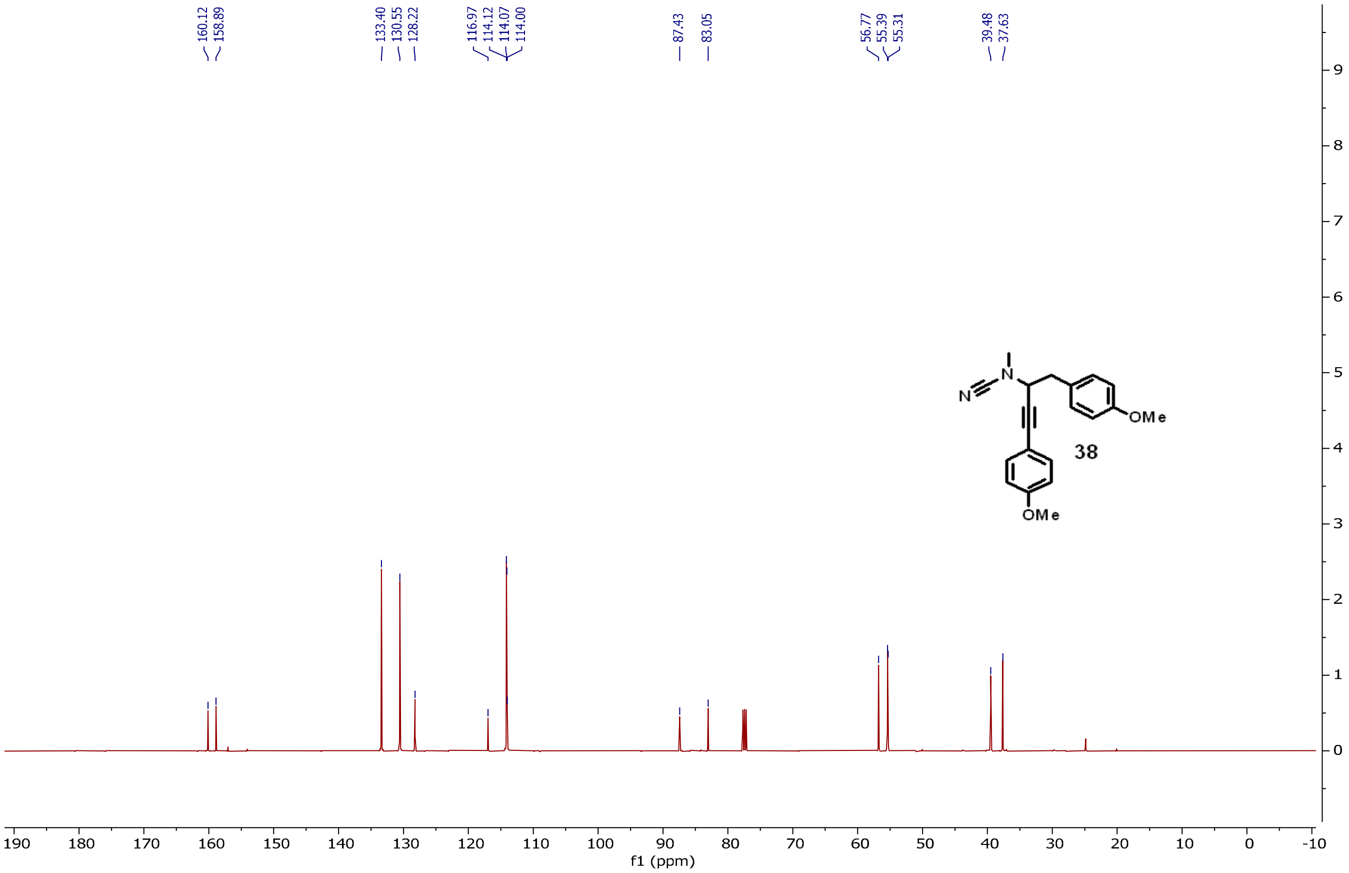

^{1}H and ^{13}C NMR of 39 in $CDCl_3$:

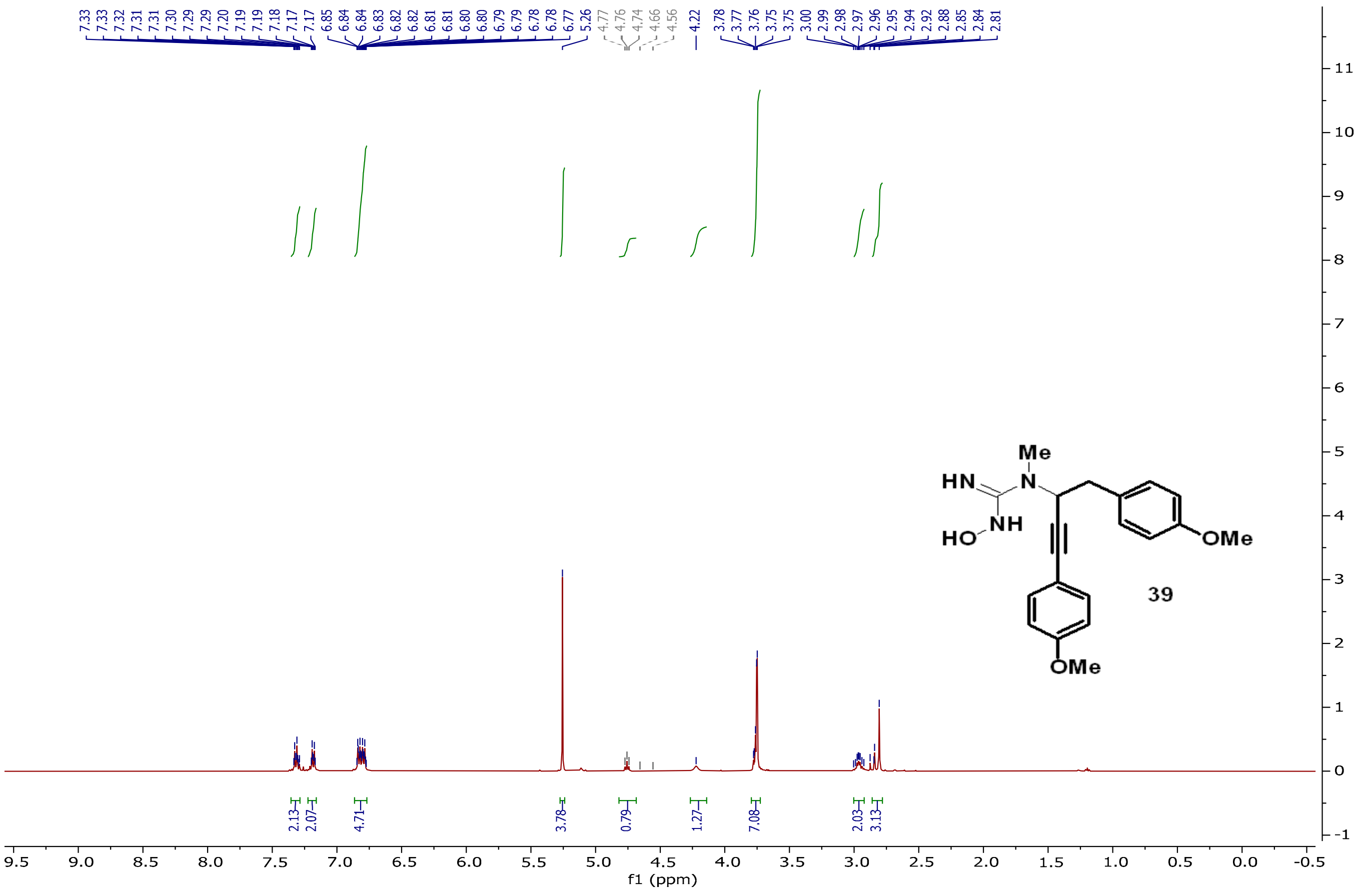

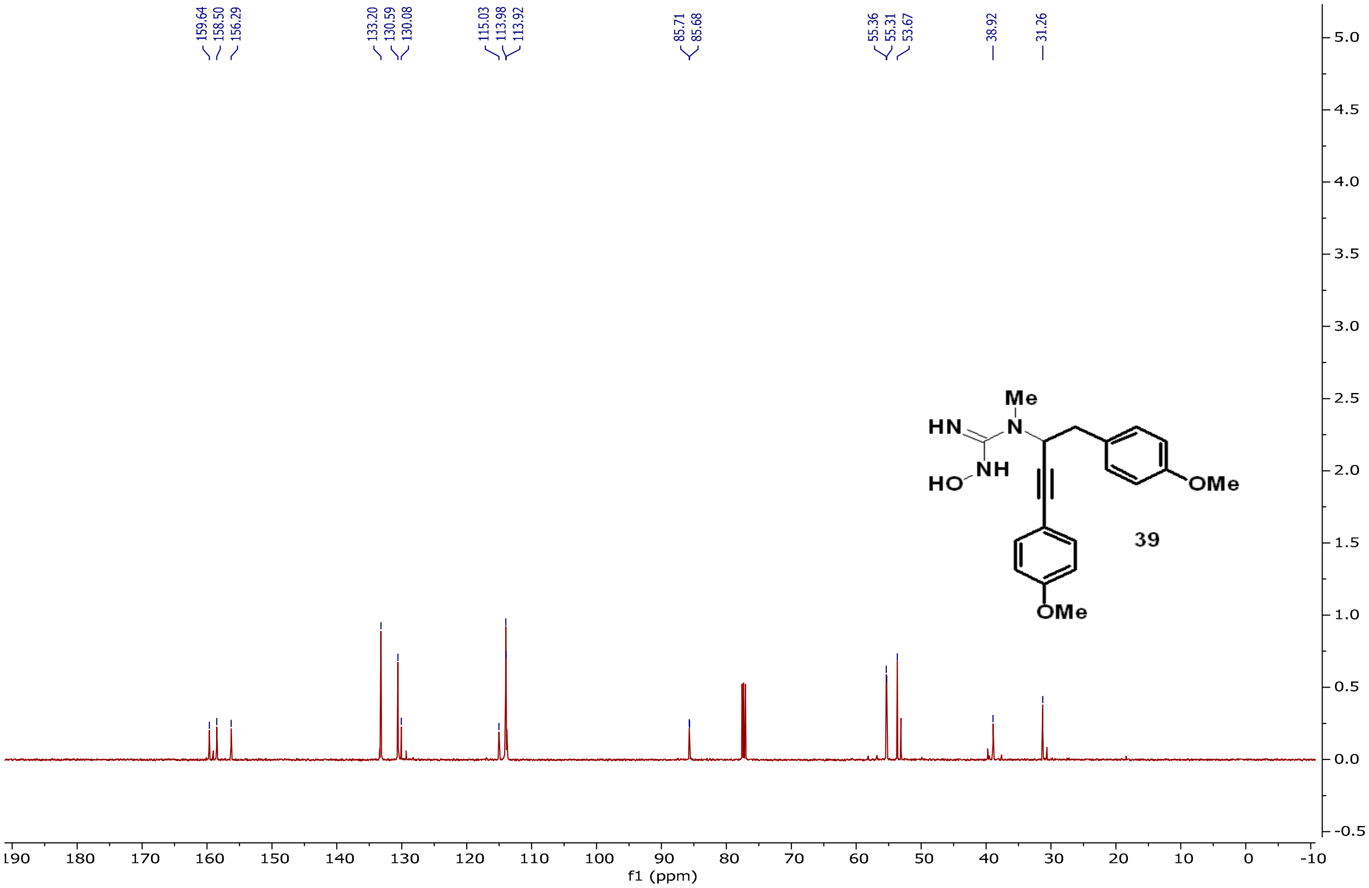

^{1}H and ^{13}C NMR of 44 in $CDCl_3$:

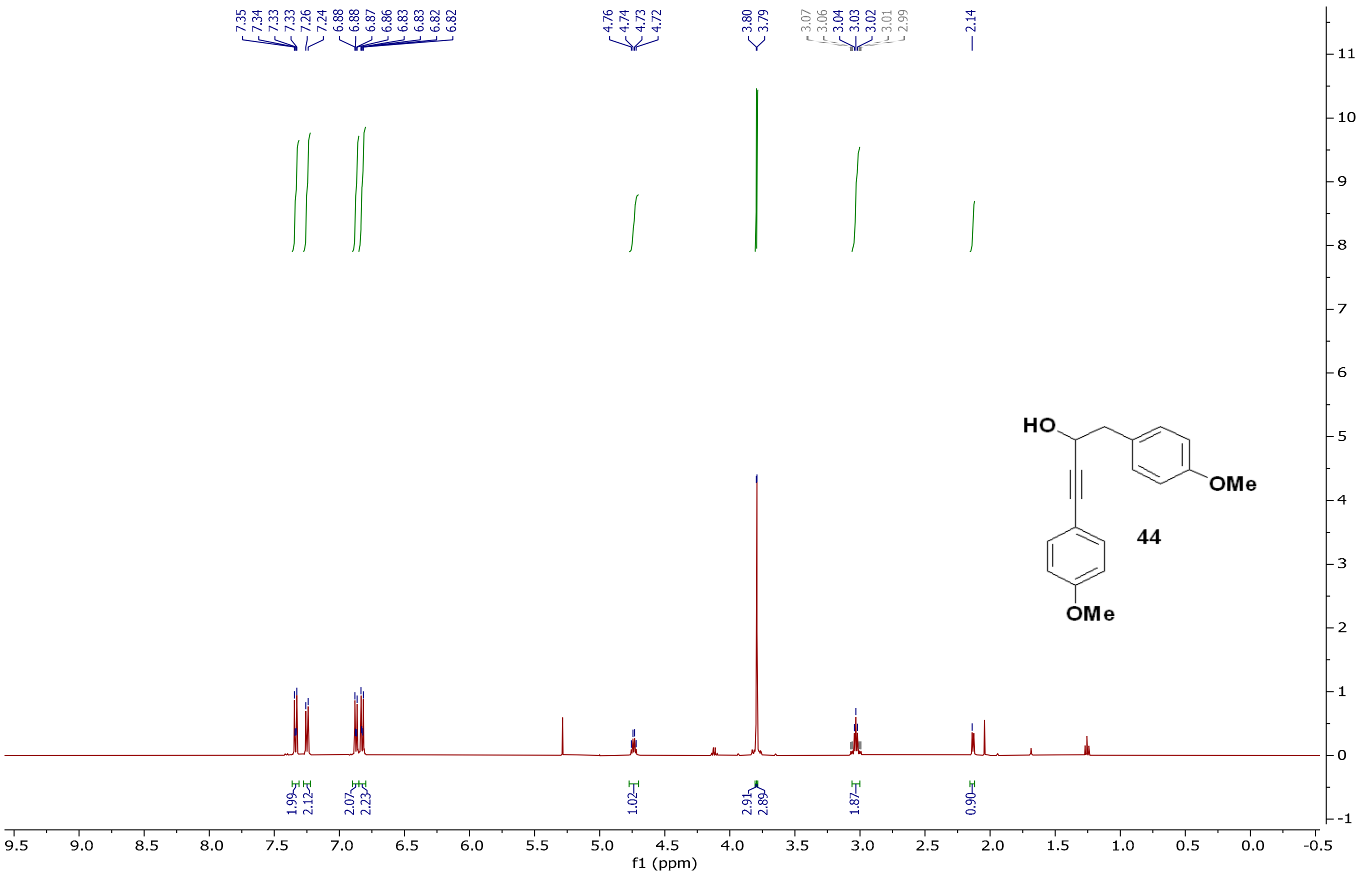

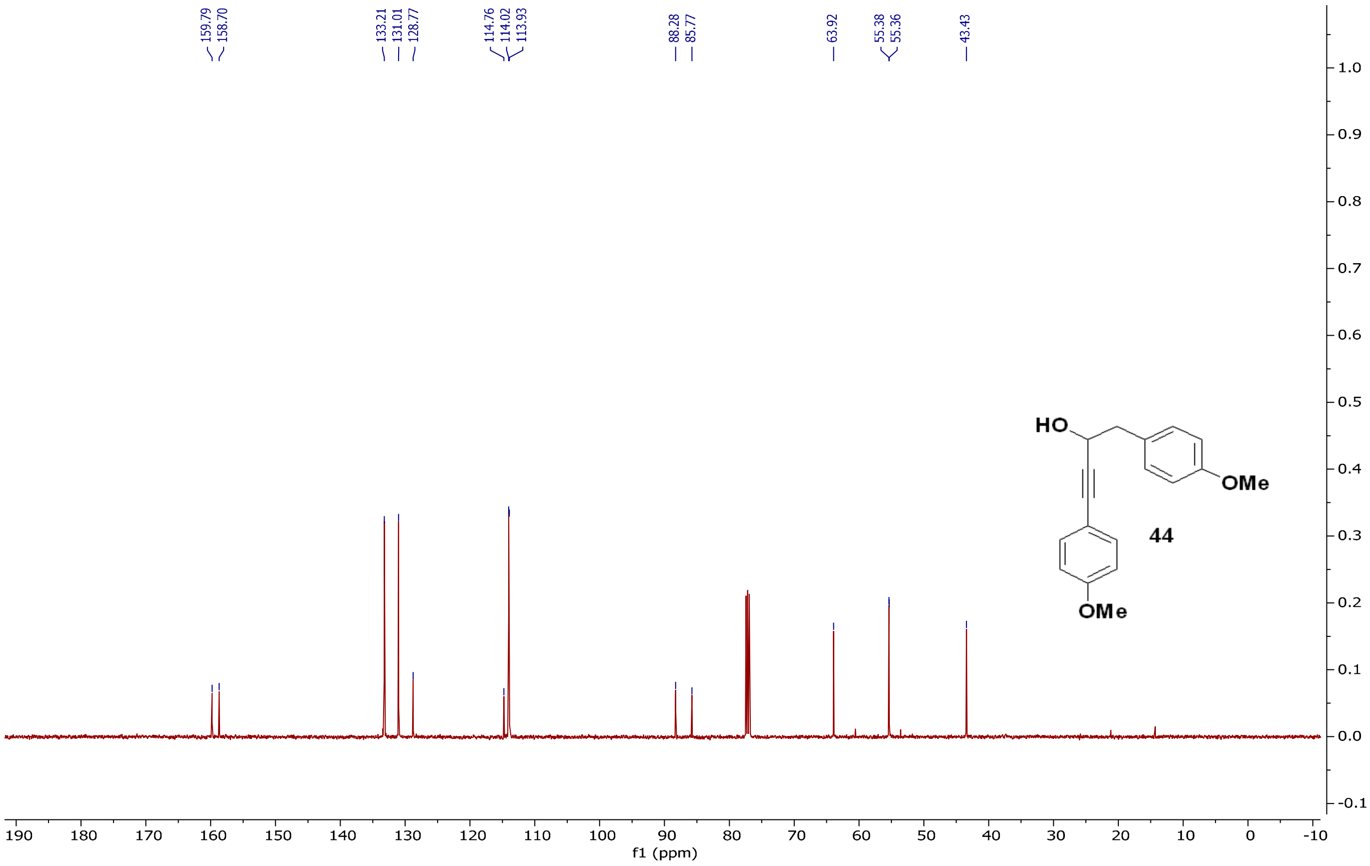

^{1}H and ^{13}C NMR of 45 in $CDCl_3$:

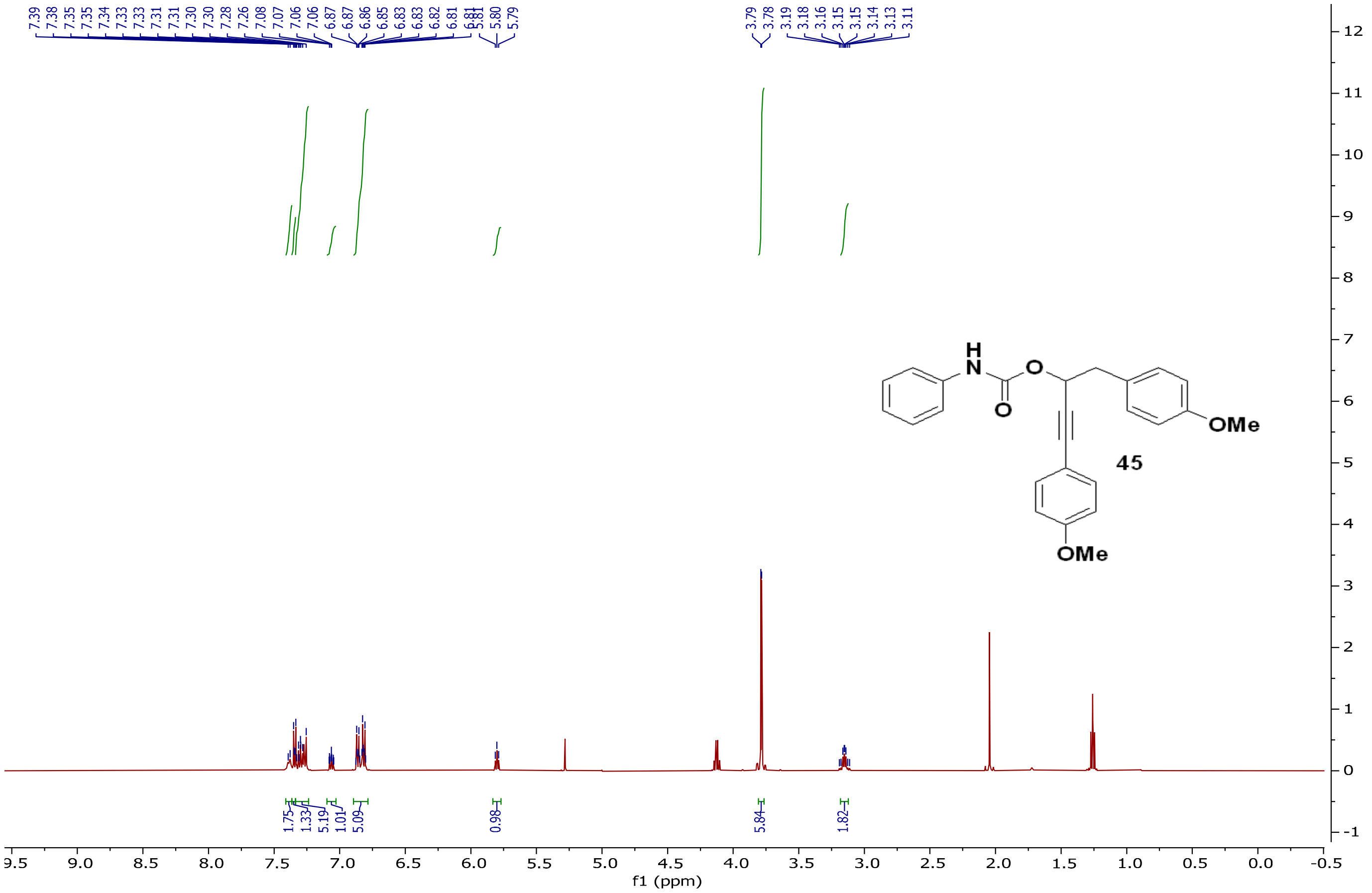

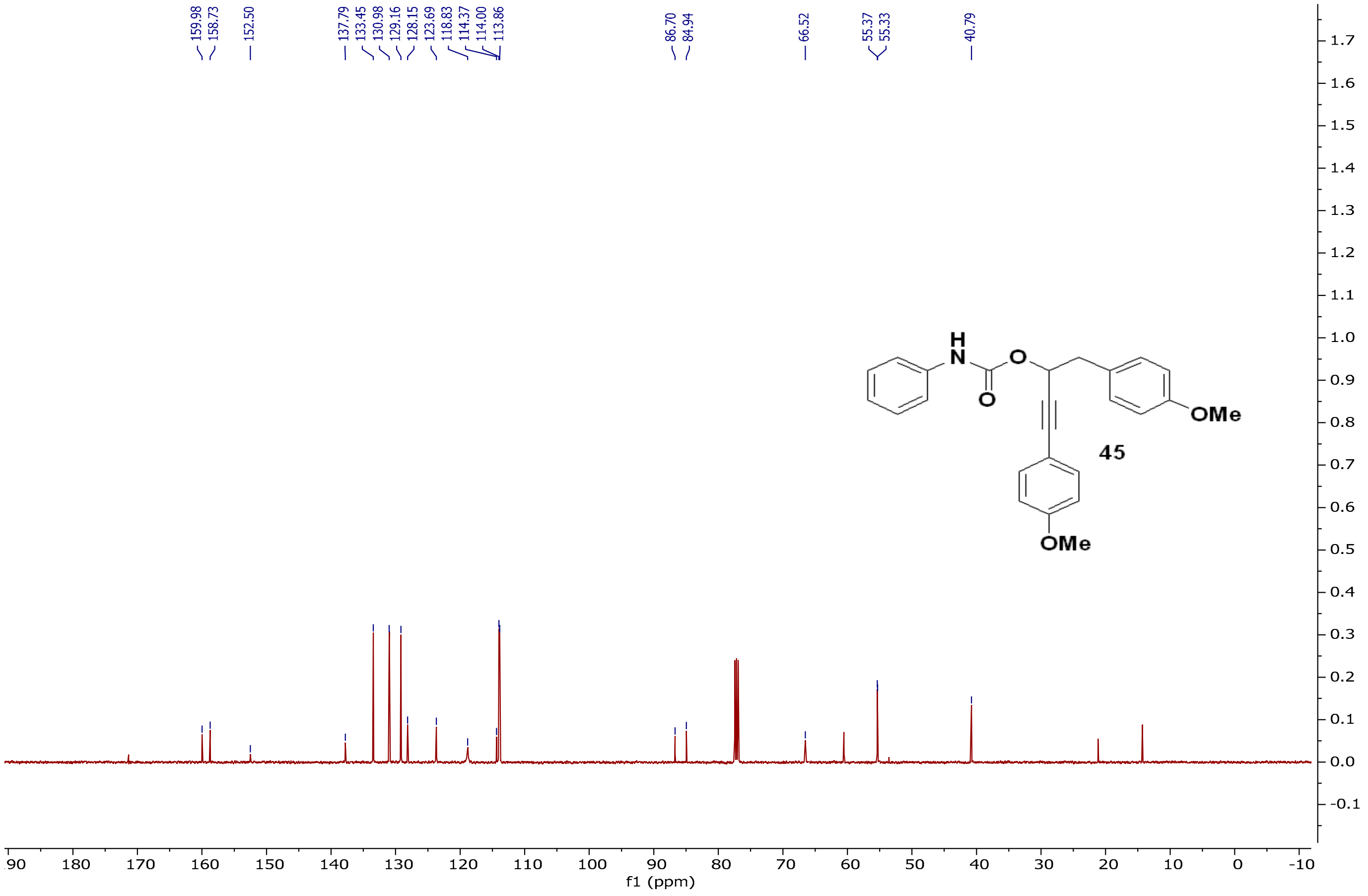

^{1}H of 47 and ^{13}C NMR of 47+47a in $CDCl_3$:

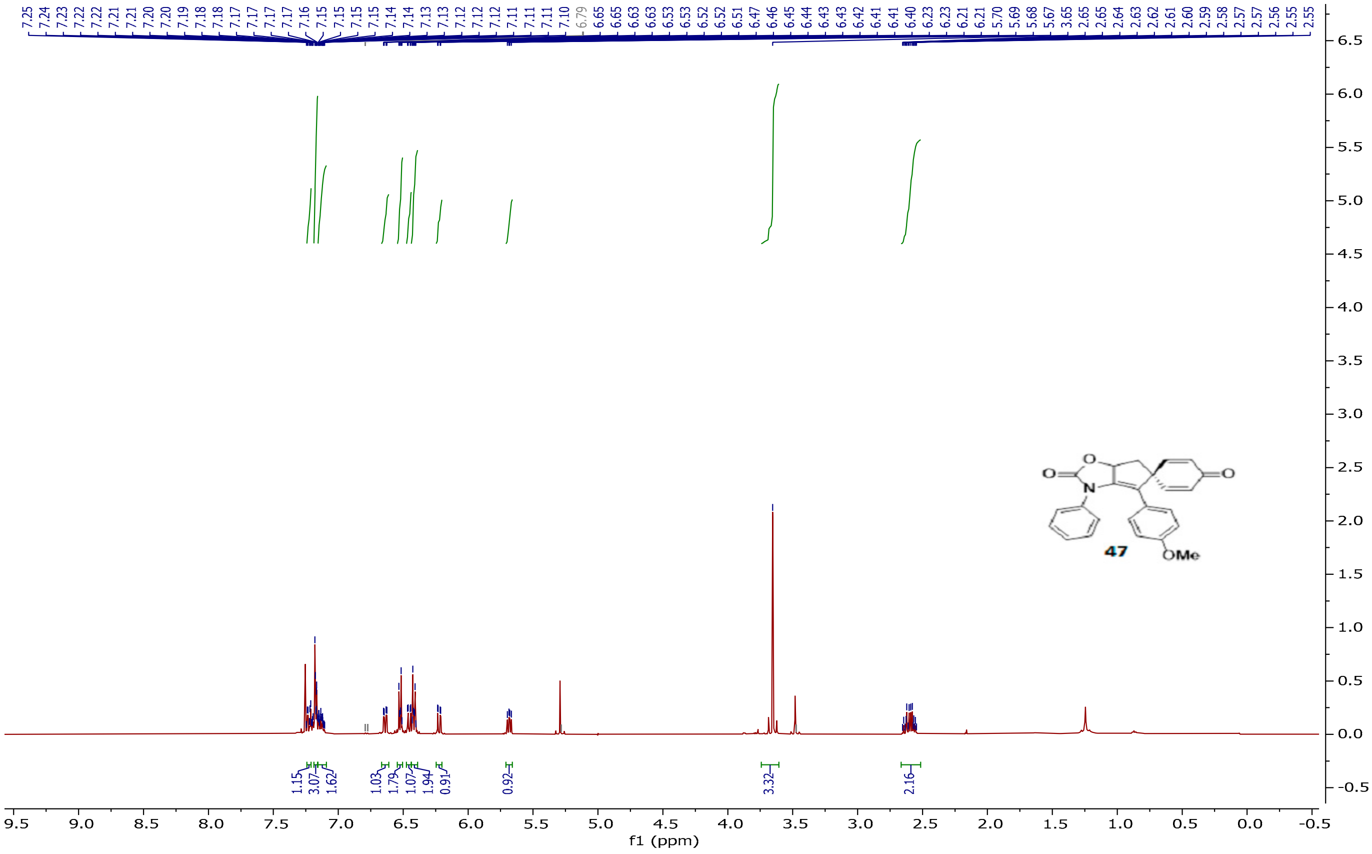

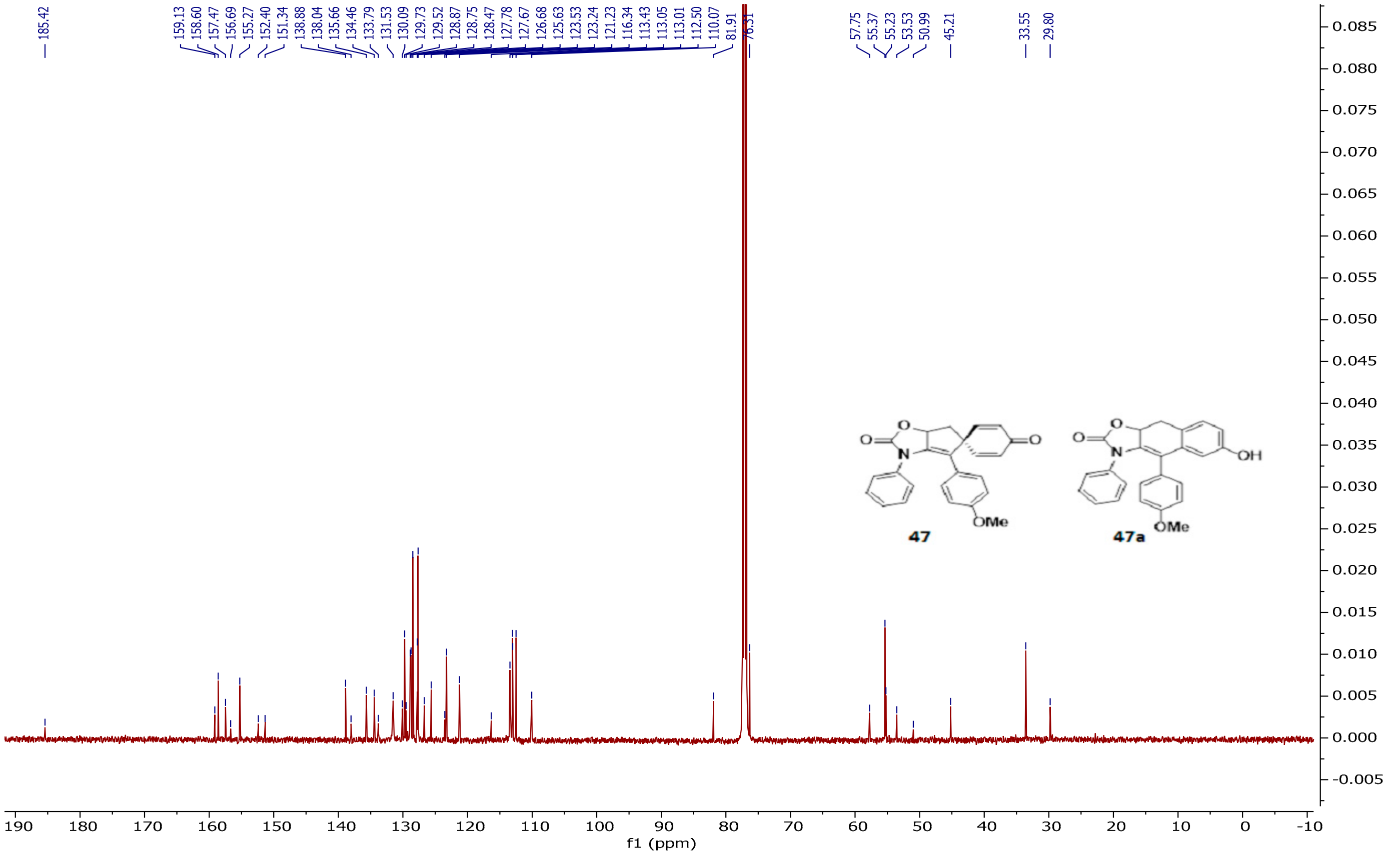

^{1}H and ^{13}C NMR of 48 in MeOD:

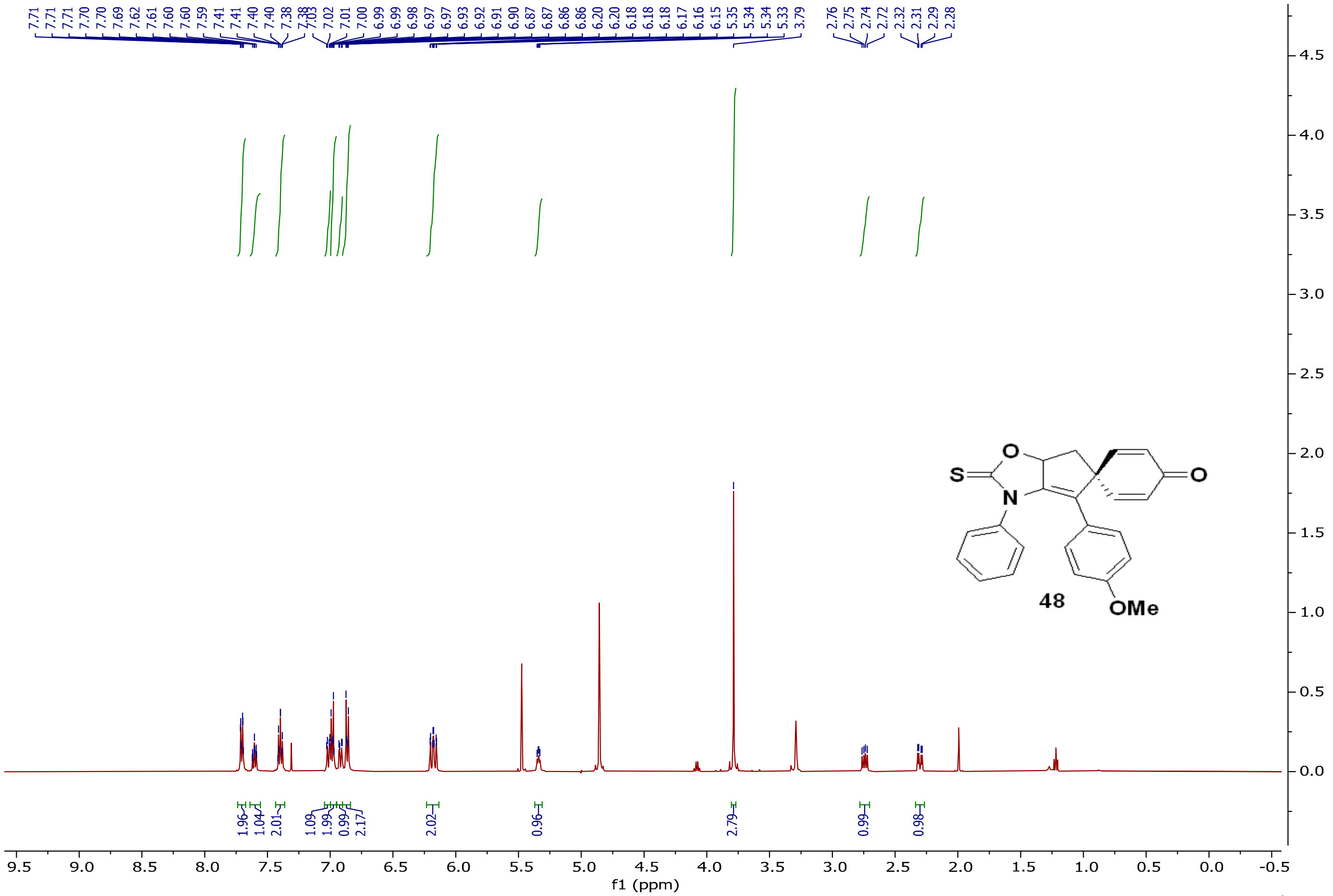

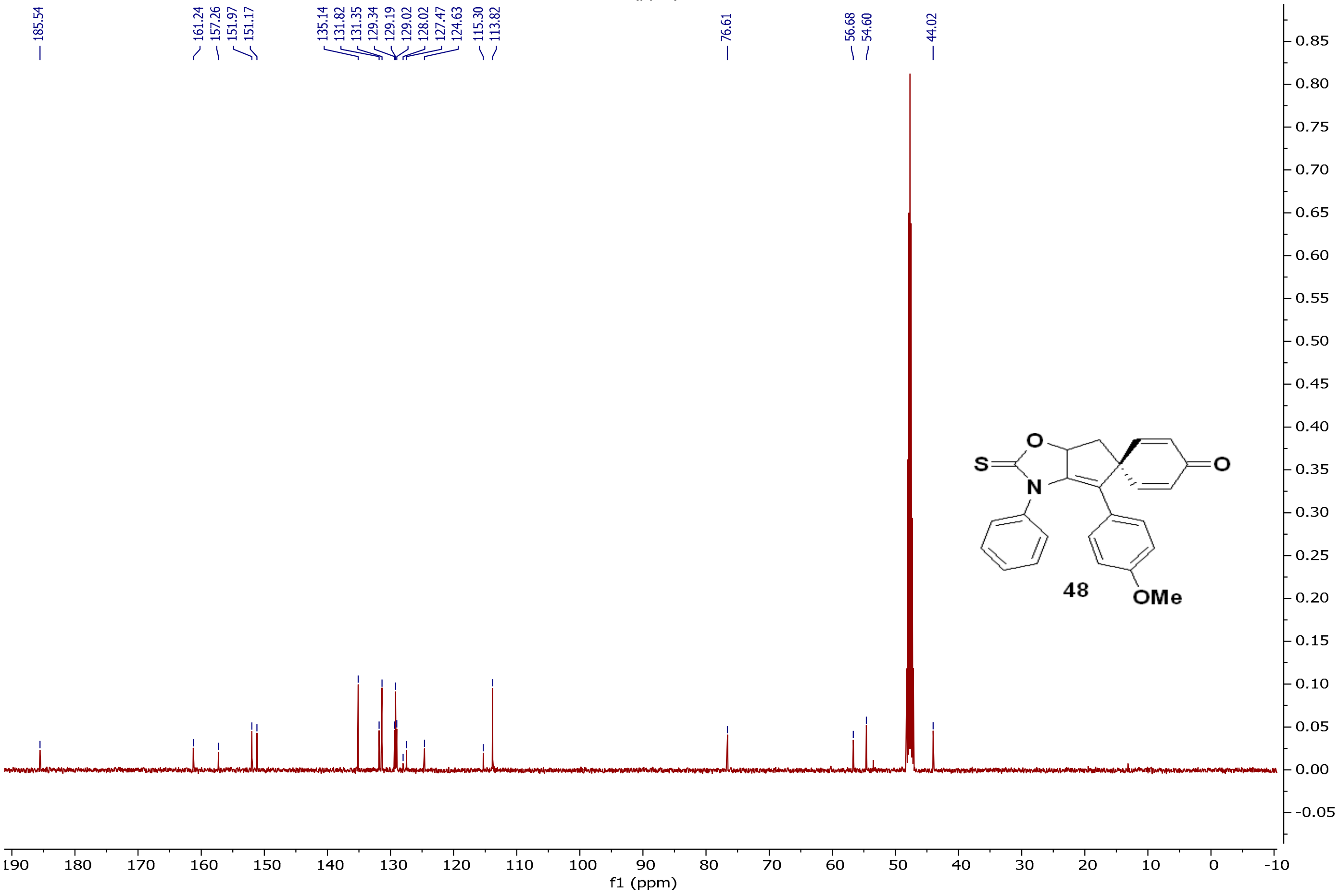

^{1}H and ^{13}C NMR of 49 in MeOD:

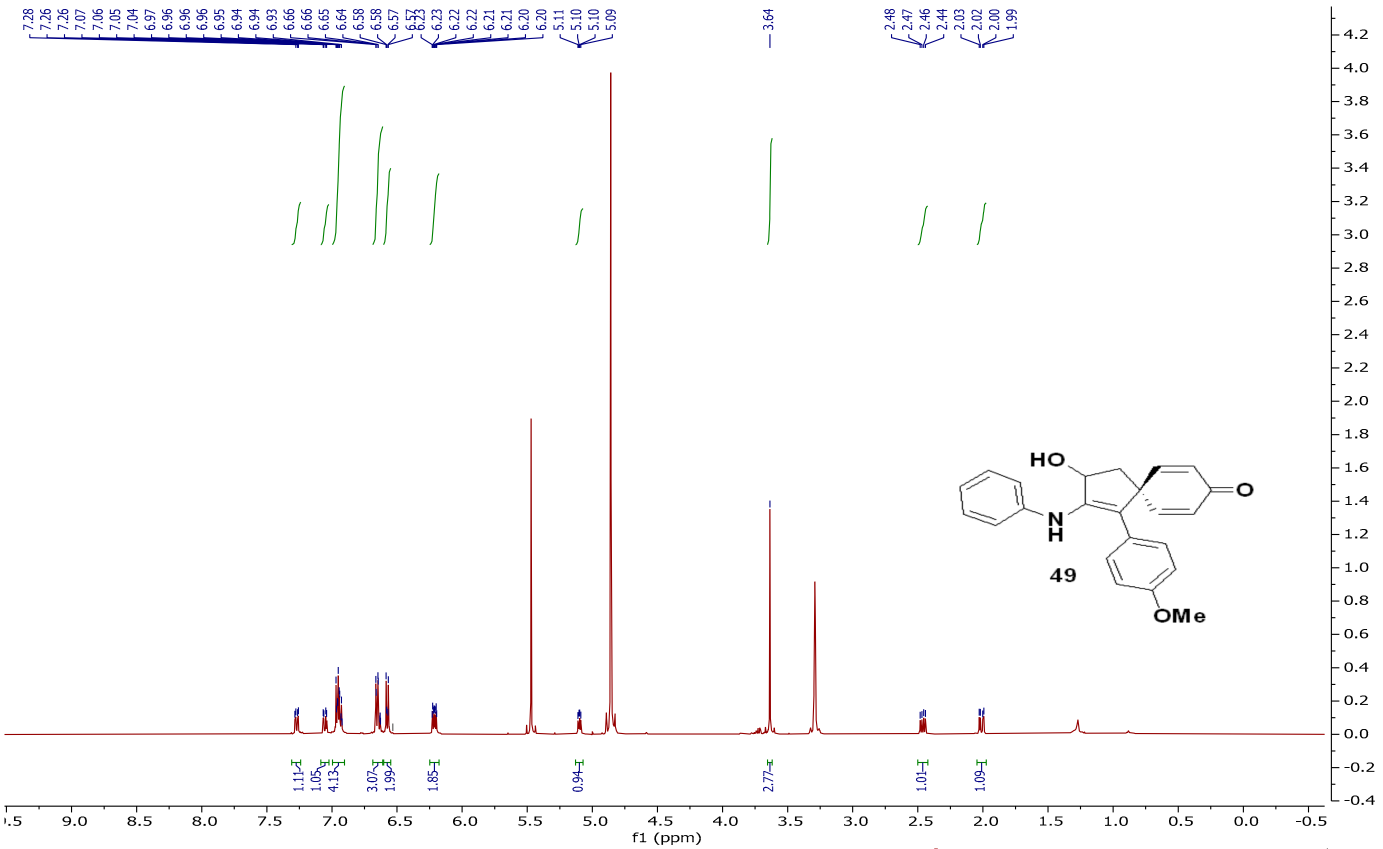

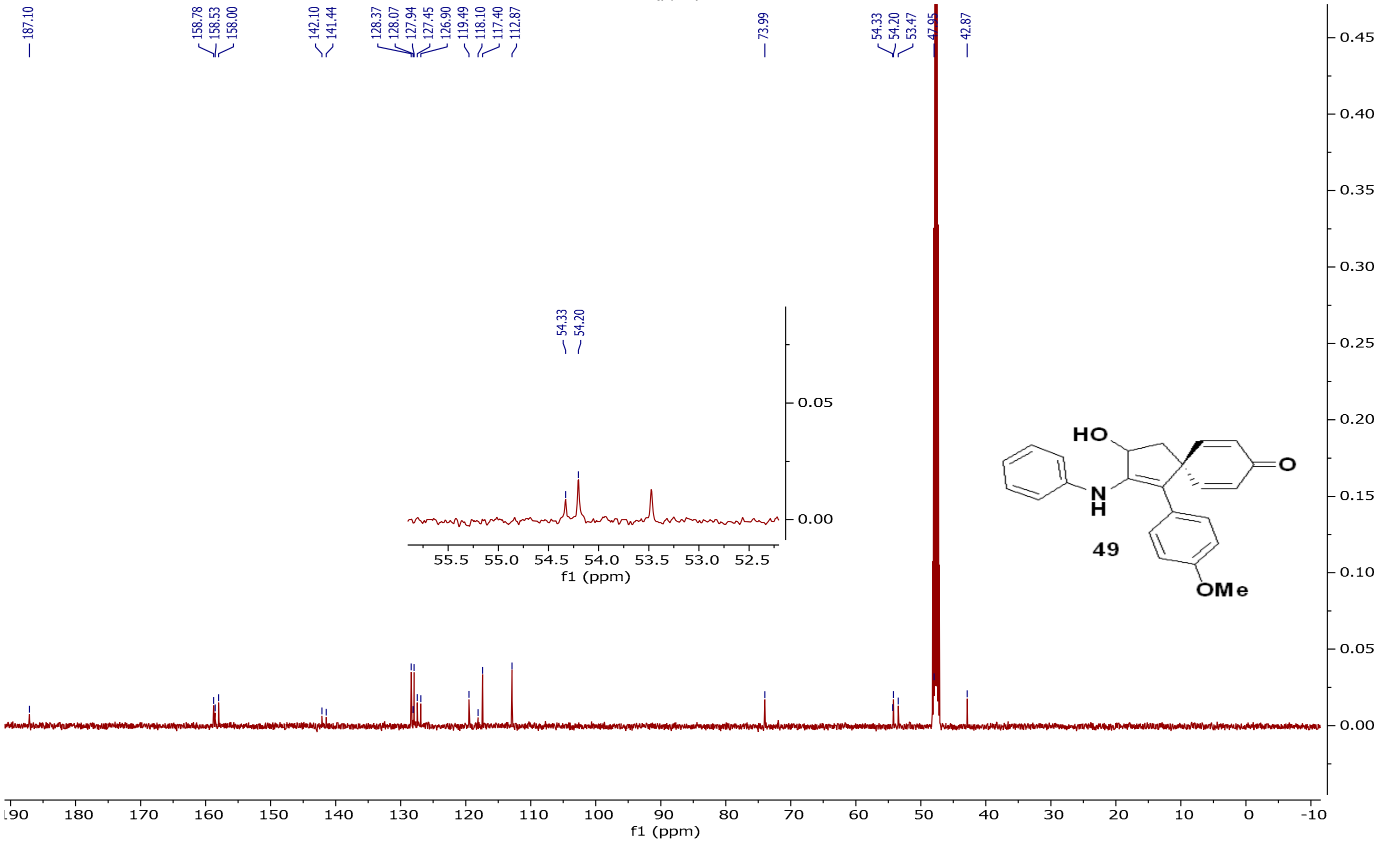

^{1}H and ^{13}C NMR of 51 in CDCl$_3$:

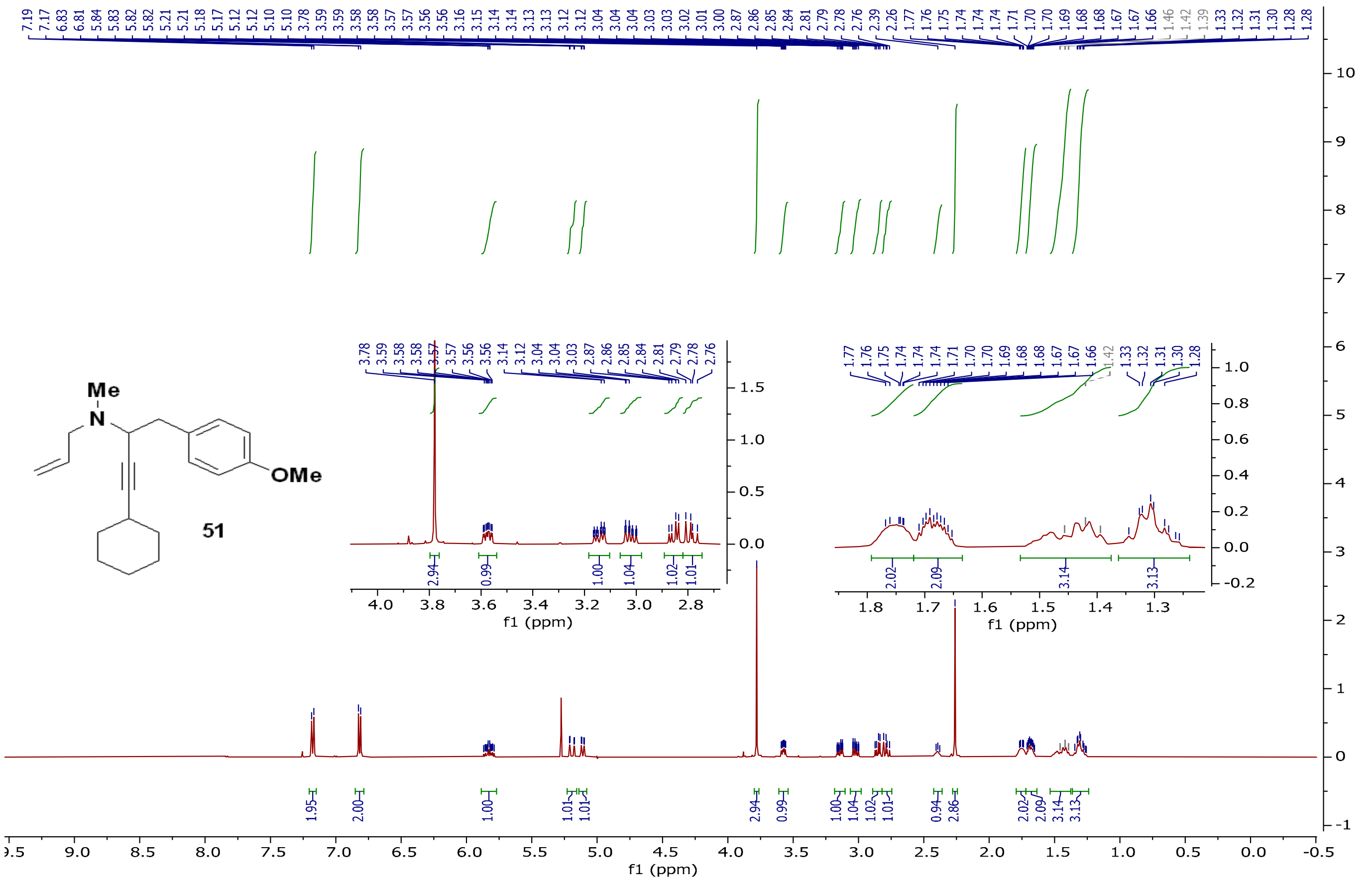

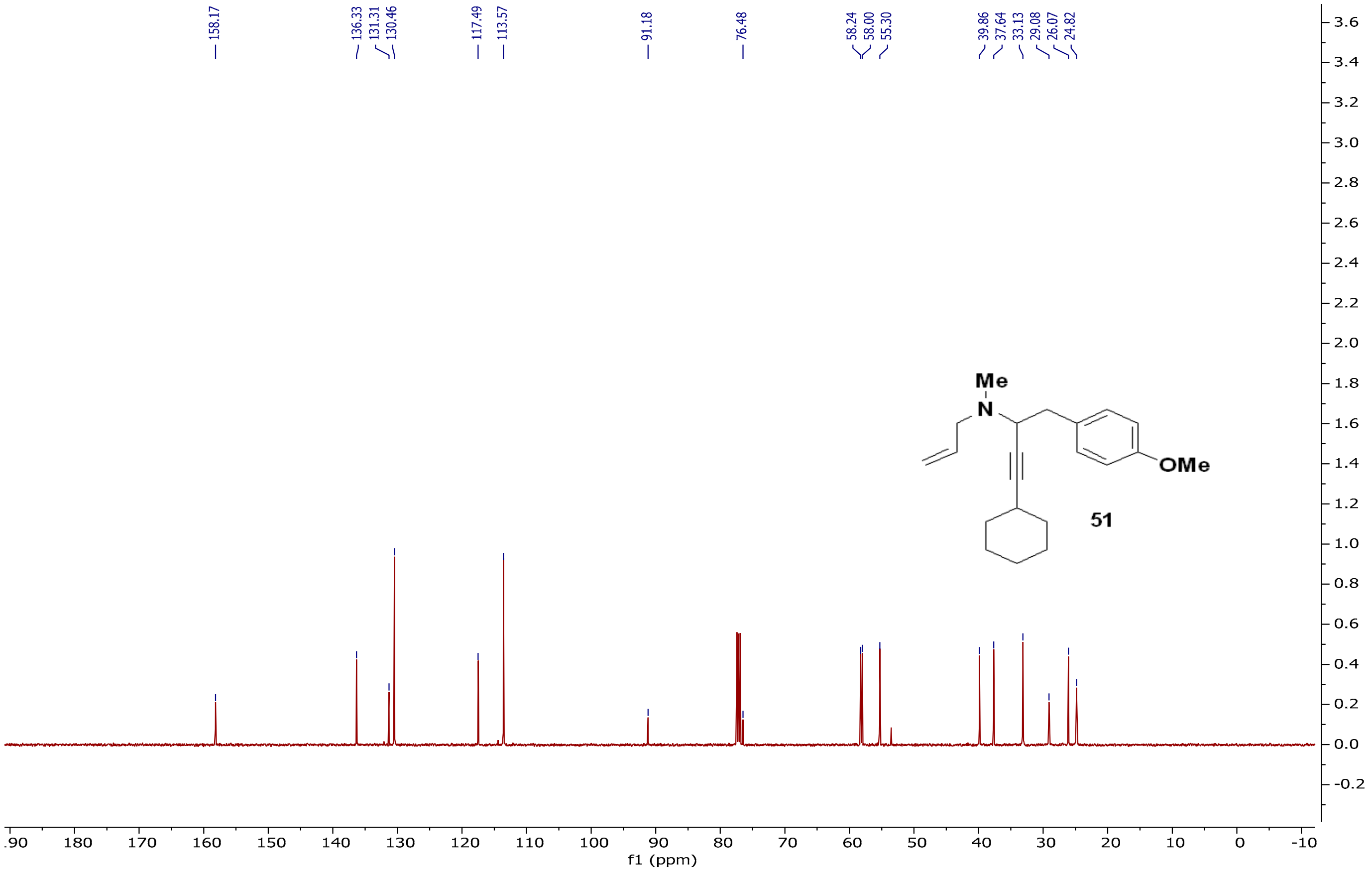

^{1}H and ^{13}C NMR of 52 in $CDCl_3$:

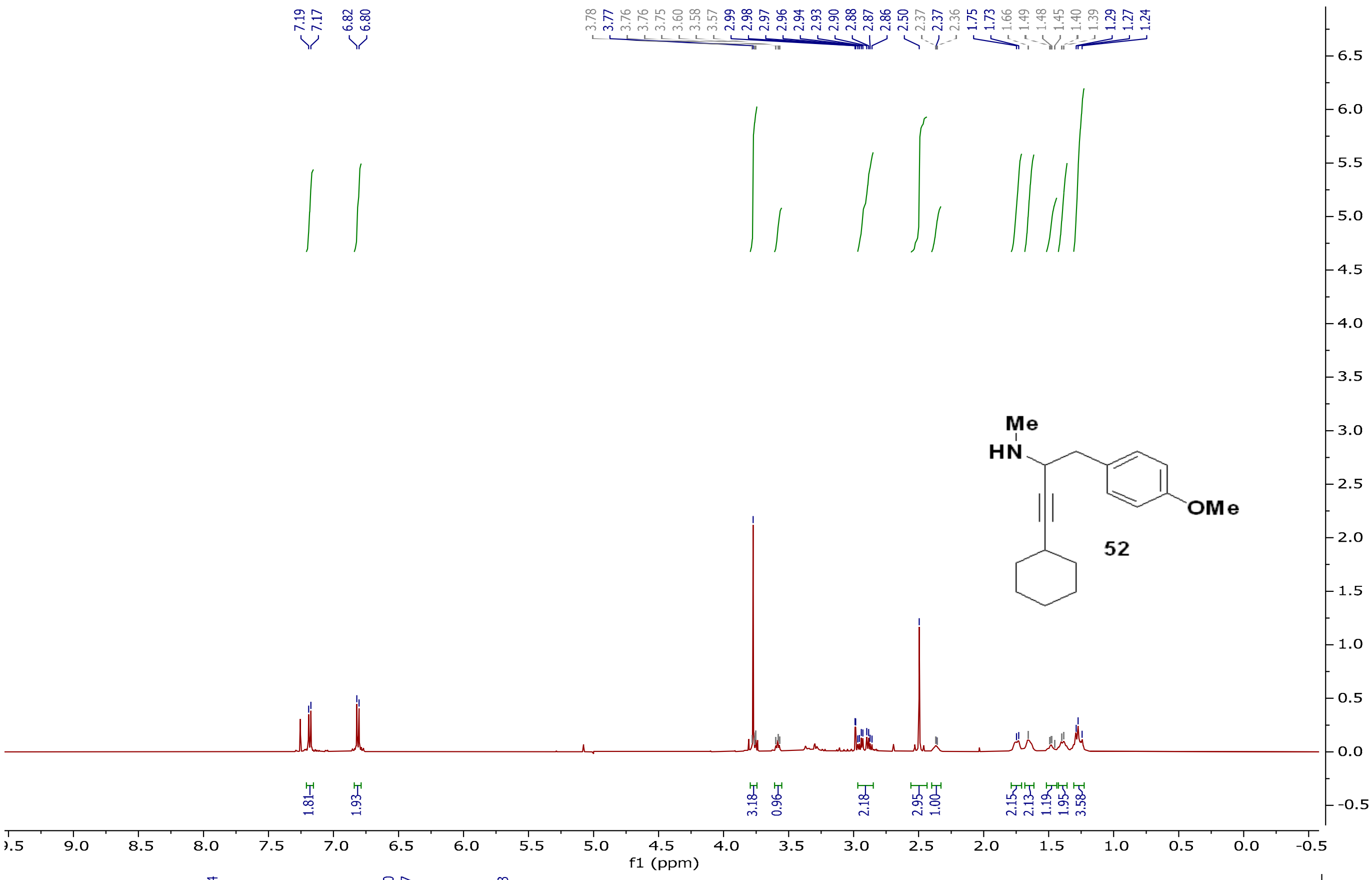

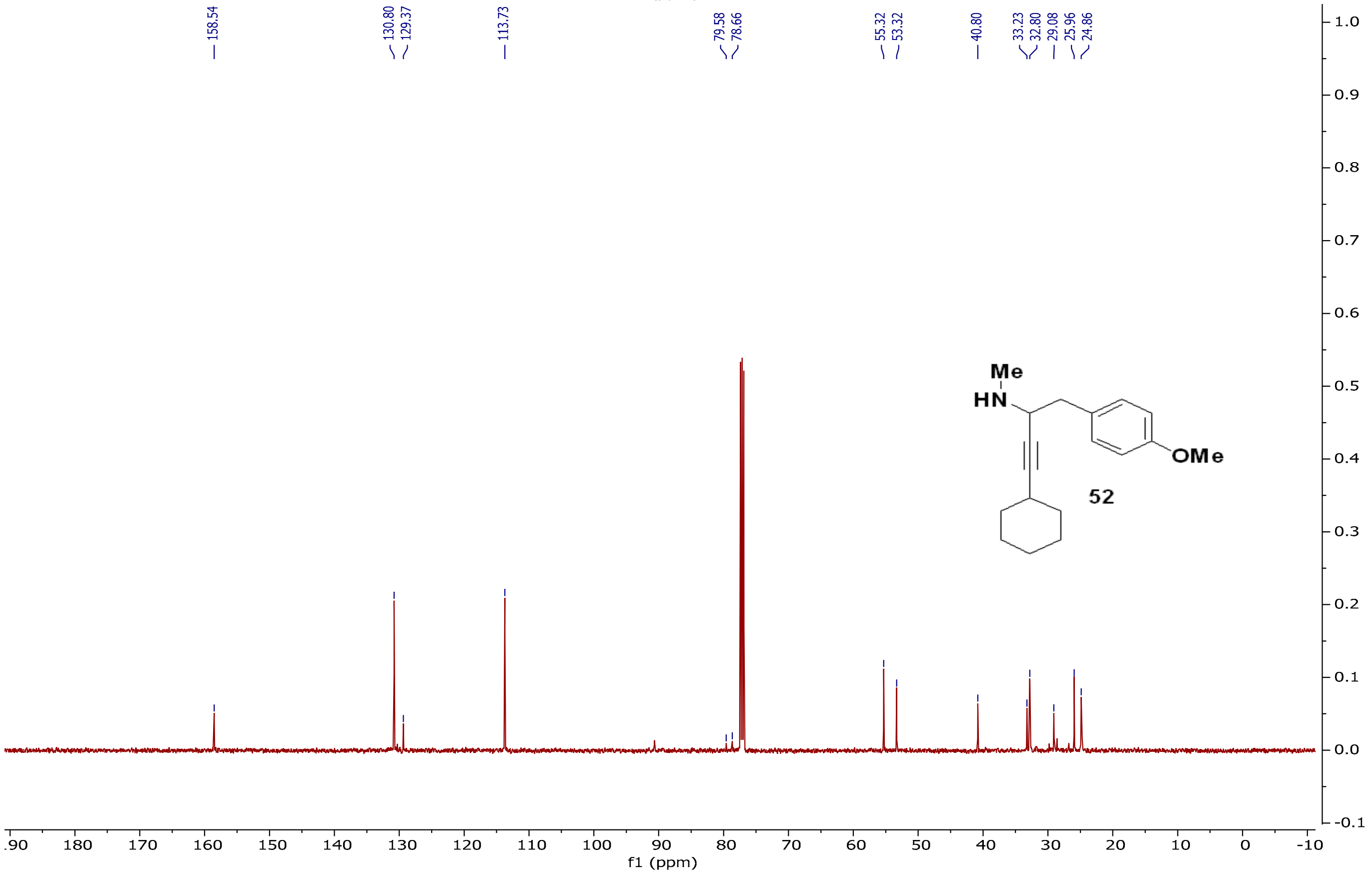

^{1}H and ^{13}C NMR of 53 in $CDCl_3$:

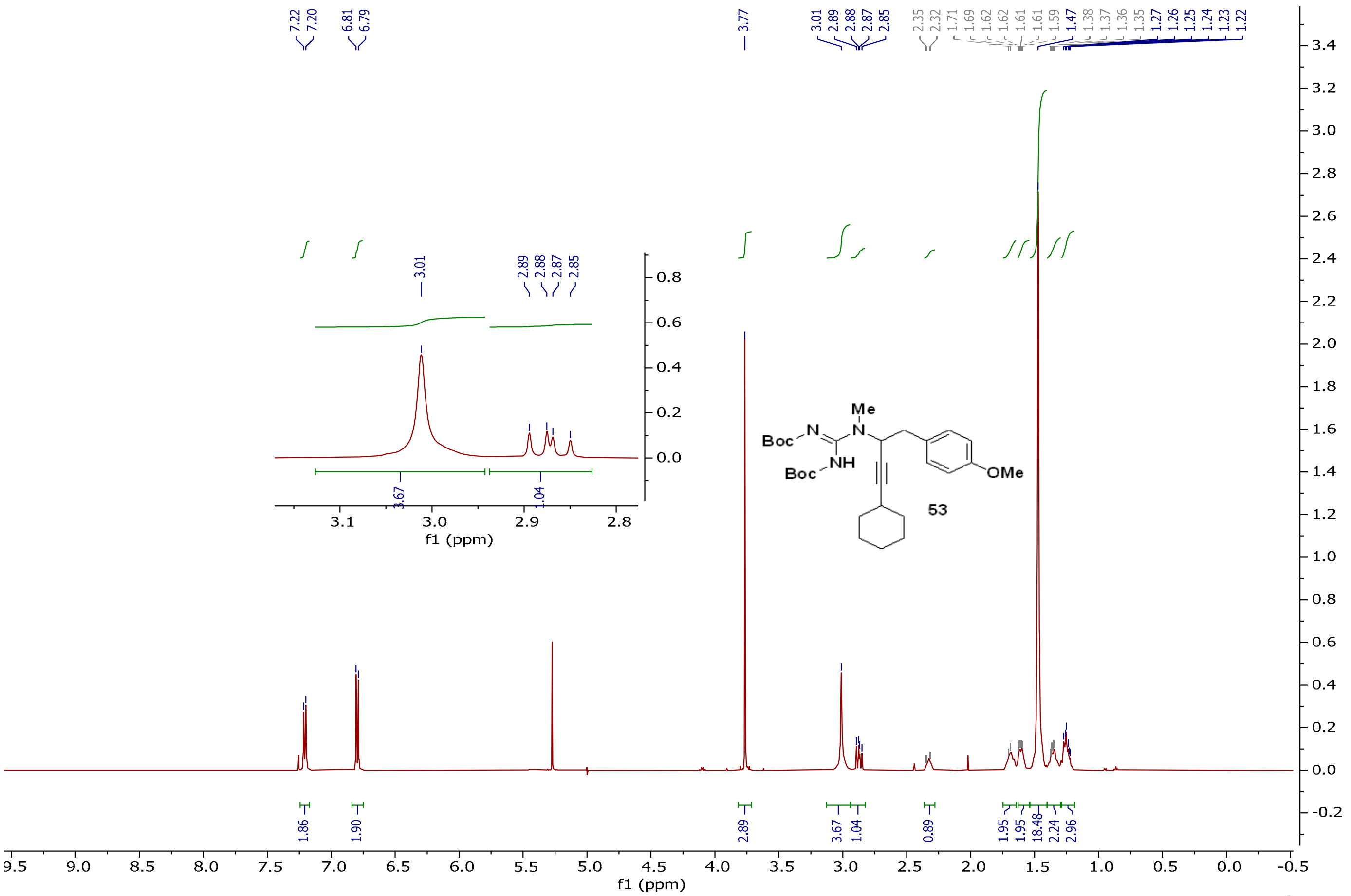

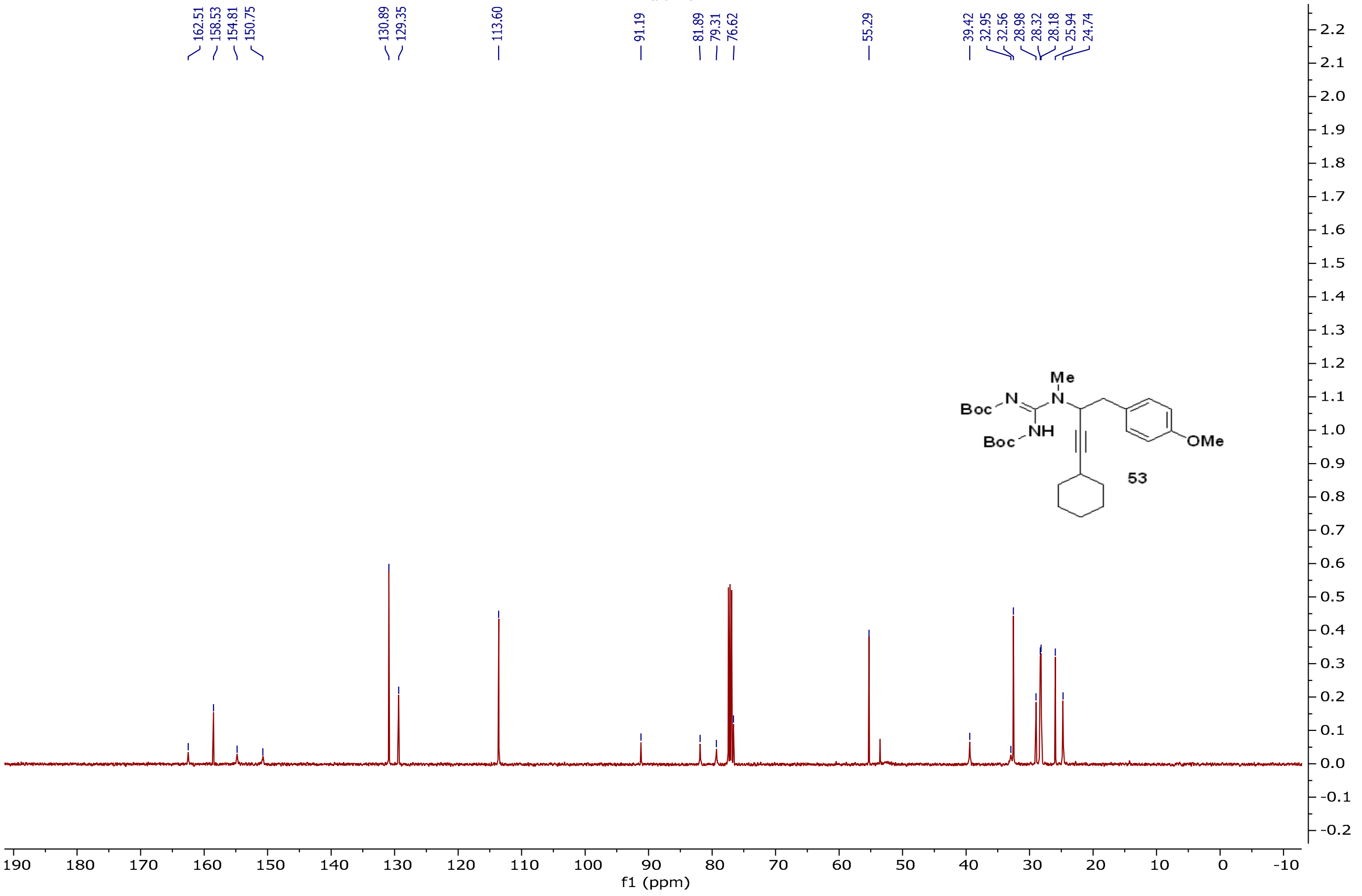

^{1}H and ^{13}C NMR of 58 in $CDCl_3$:

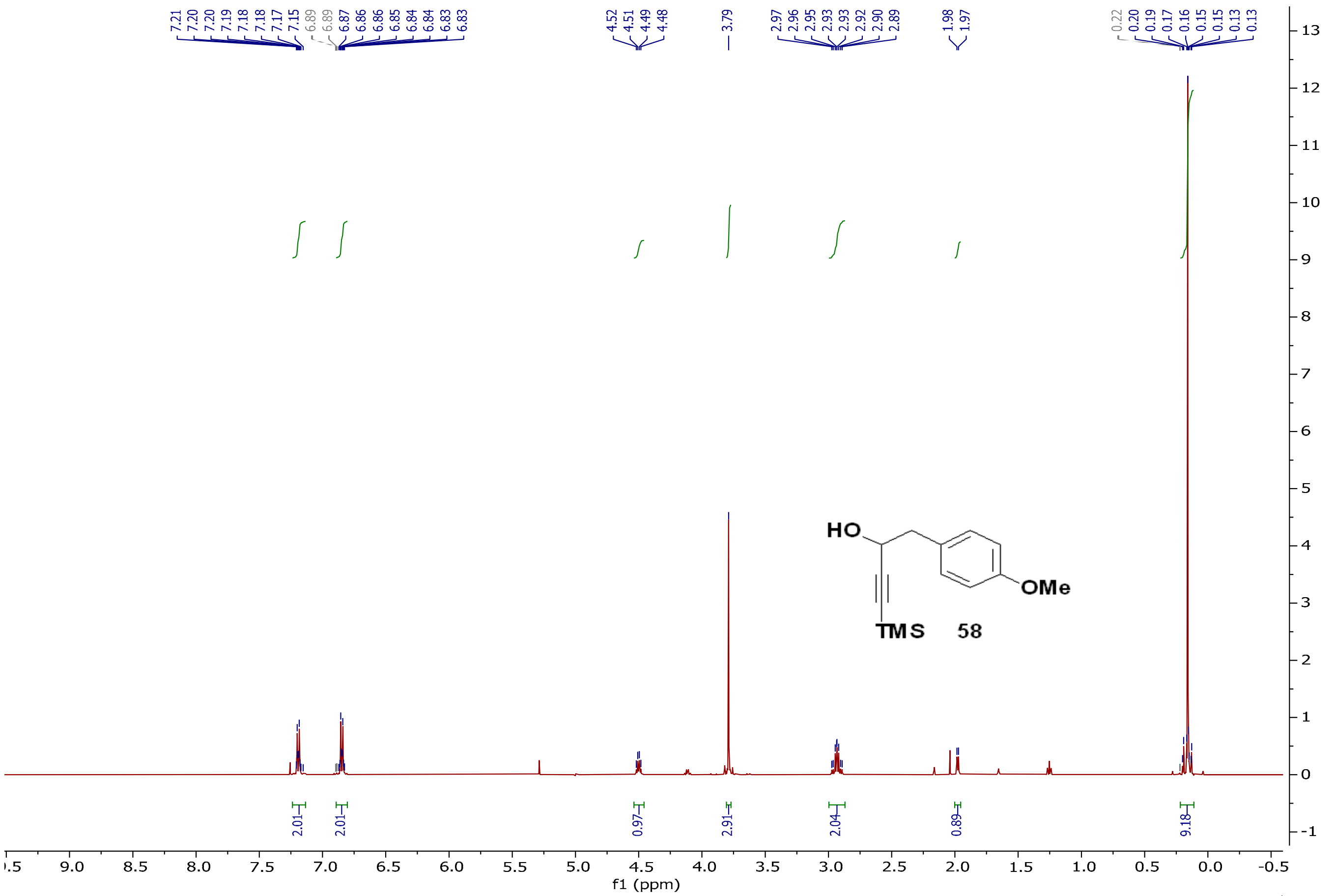

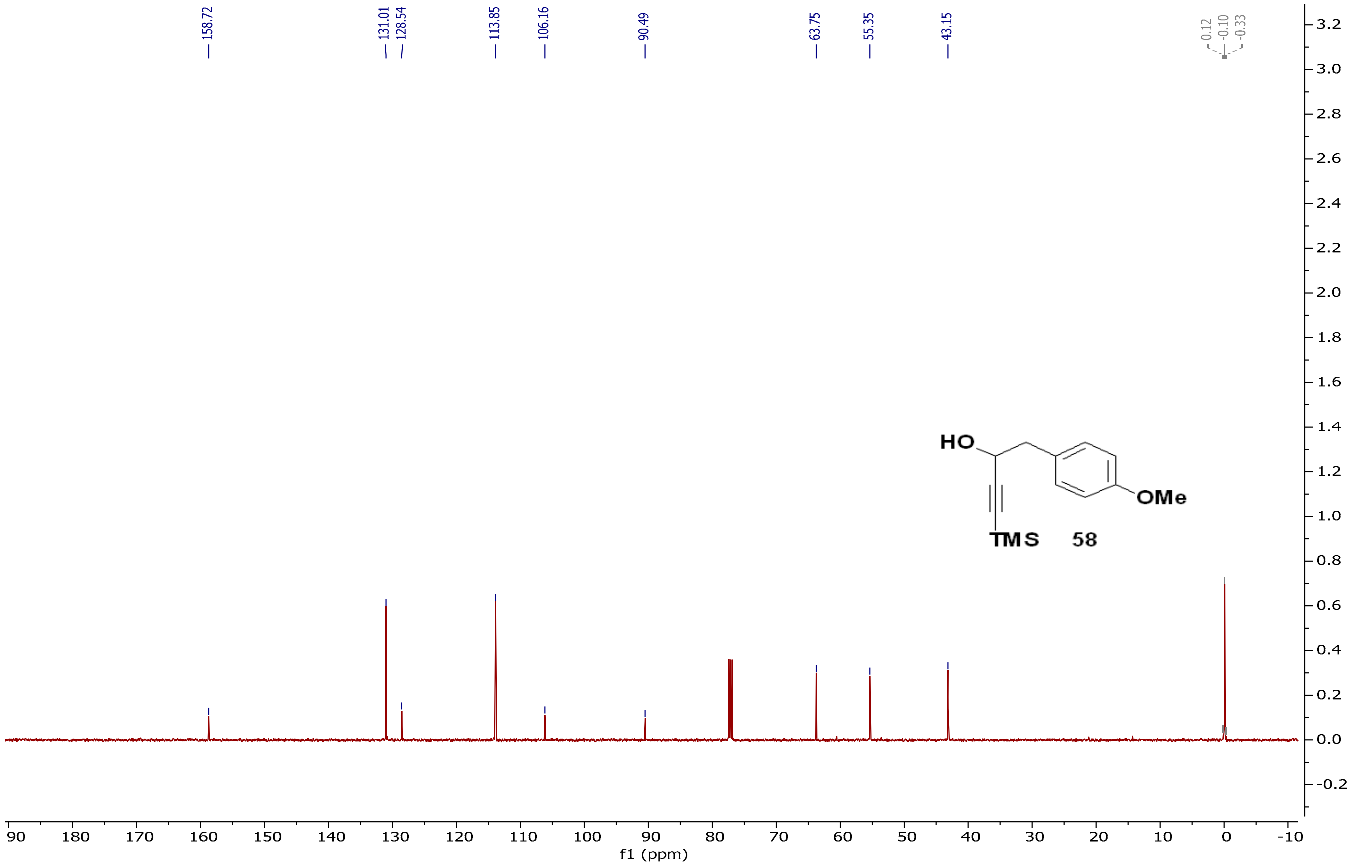

^{1}H and ^{13}C NMR of 59 in $CDCl_3$:

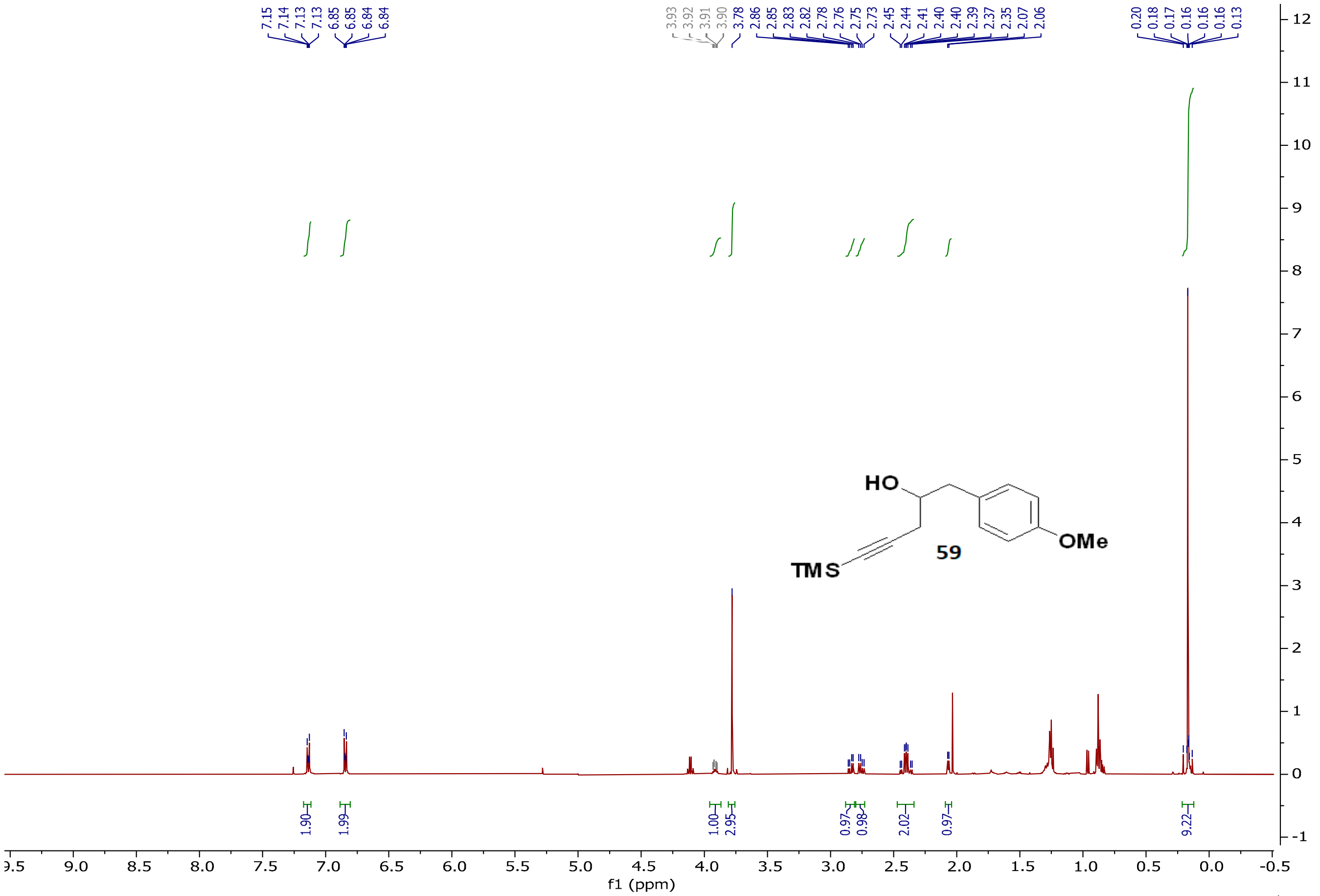

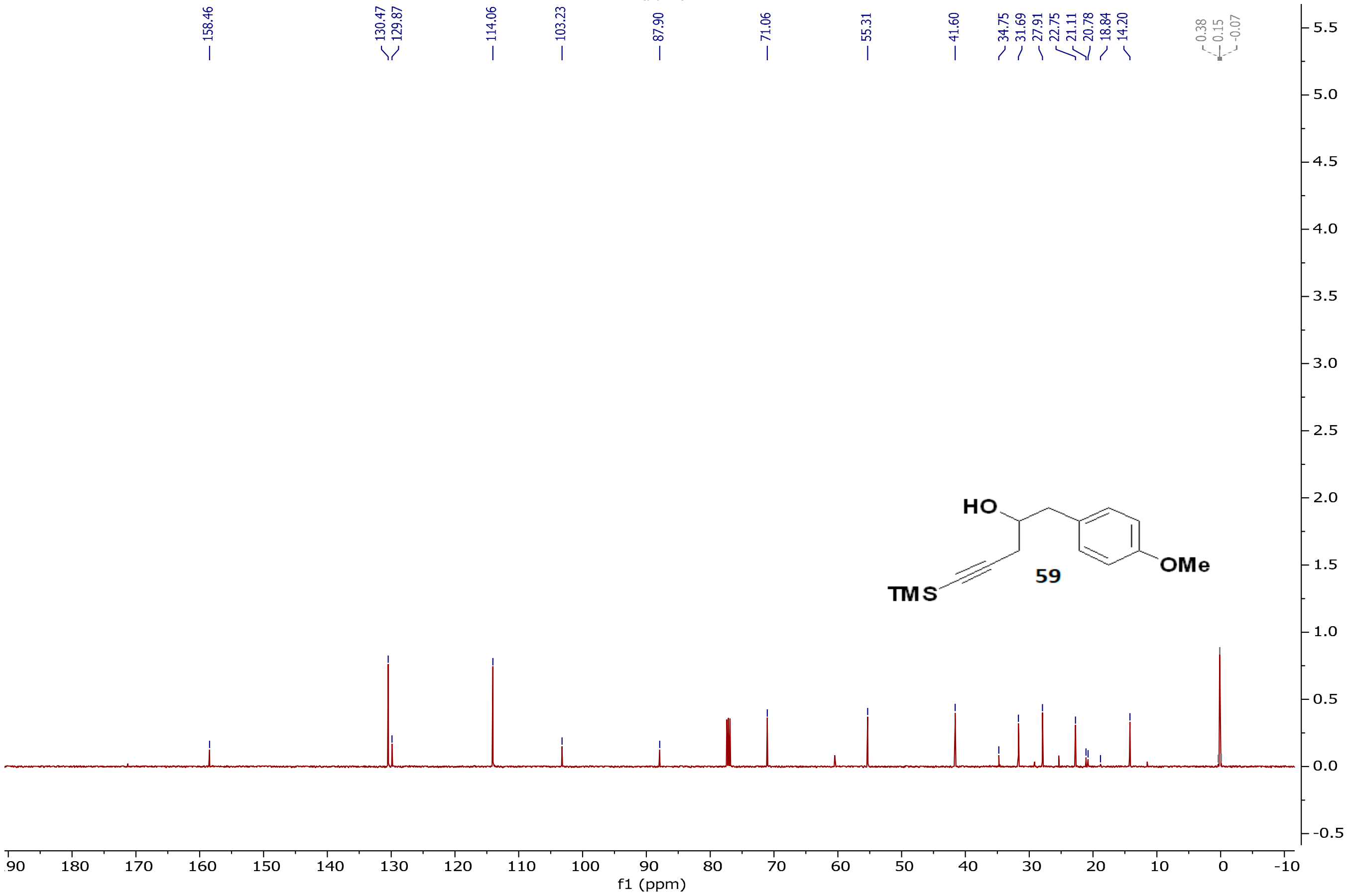

^{1}H and ^{13}C NMR of 60 in $CDCl_3$:

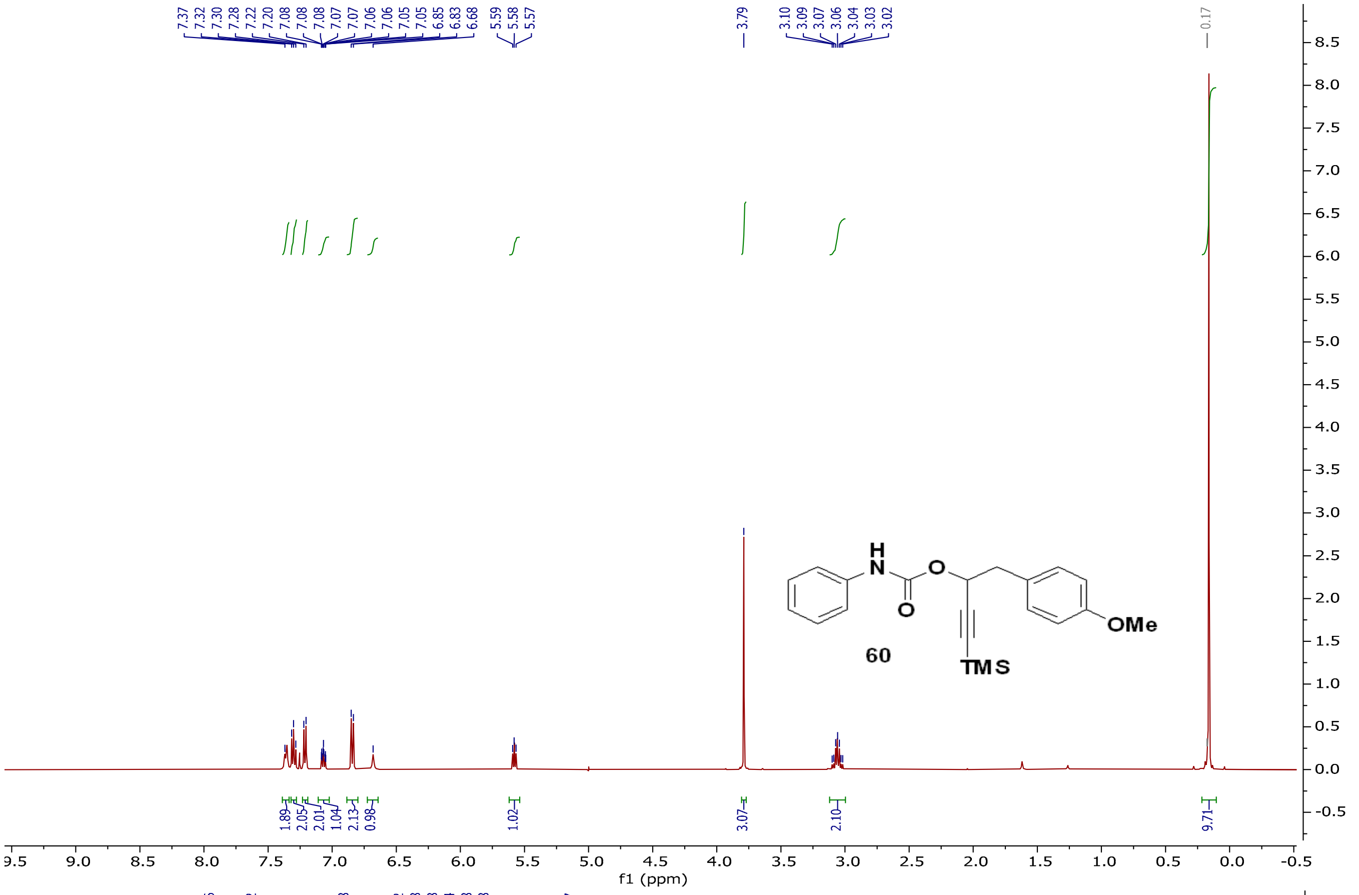

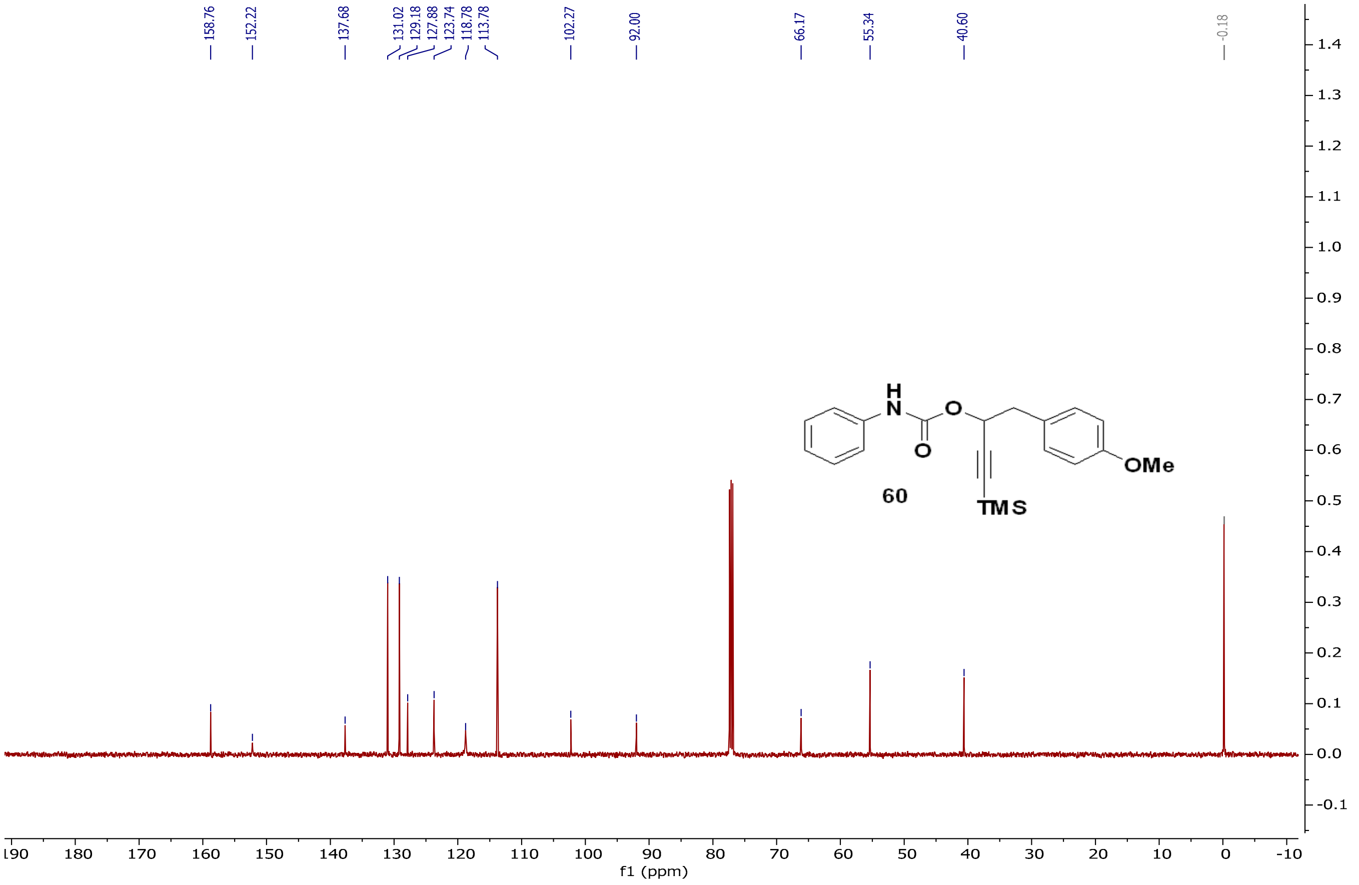

^{1}H NMR of 61 in $CDCl_3$:

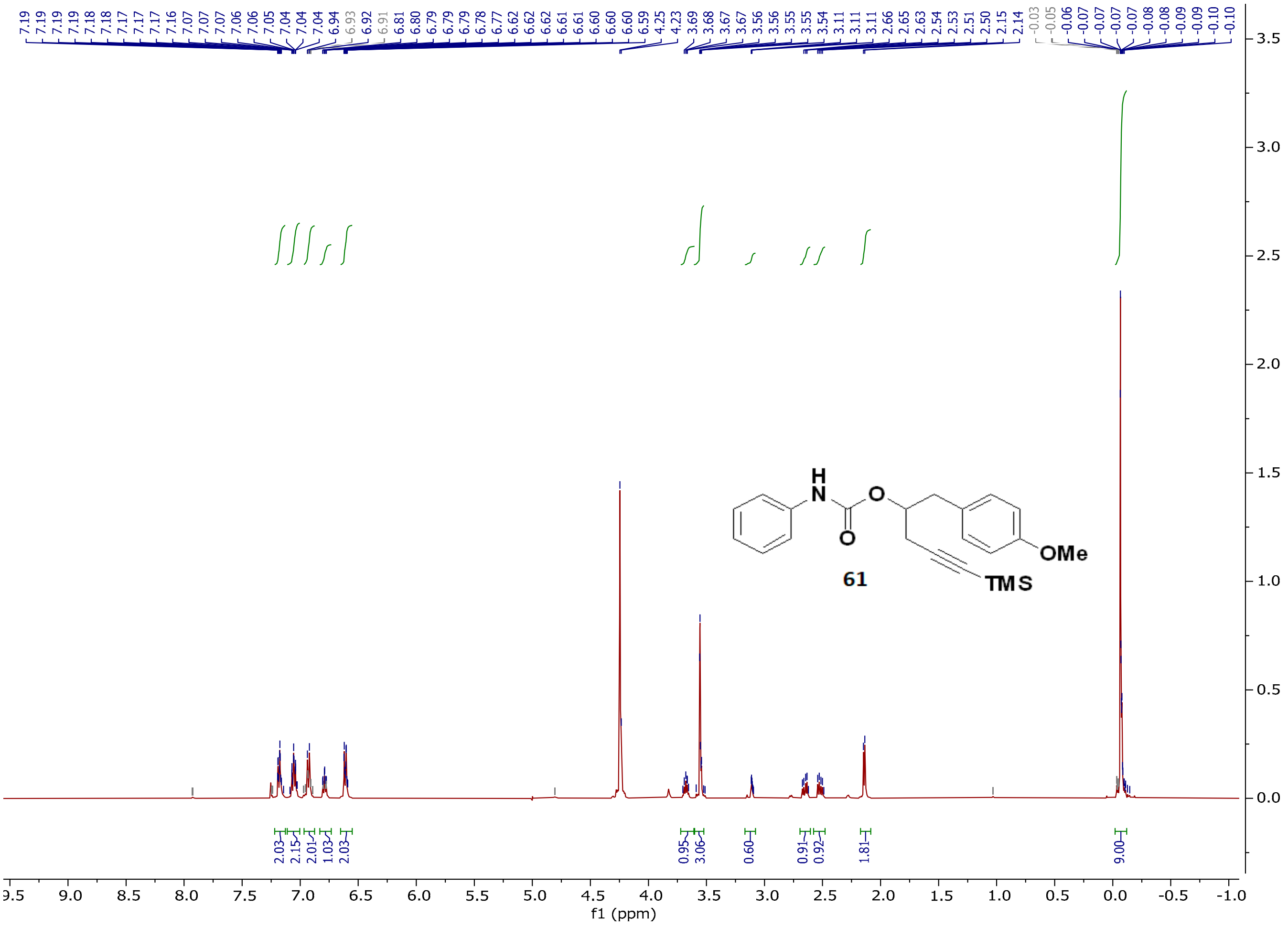

^{1}H and ^{13}C NMR of 65 in MeOD:

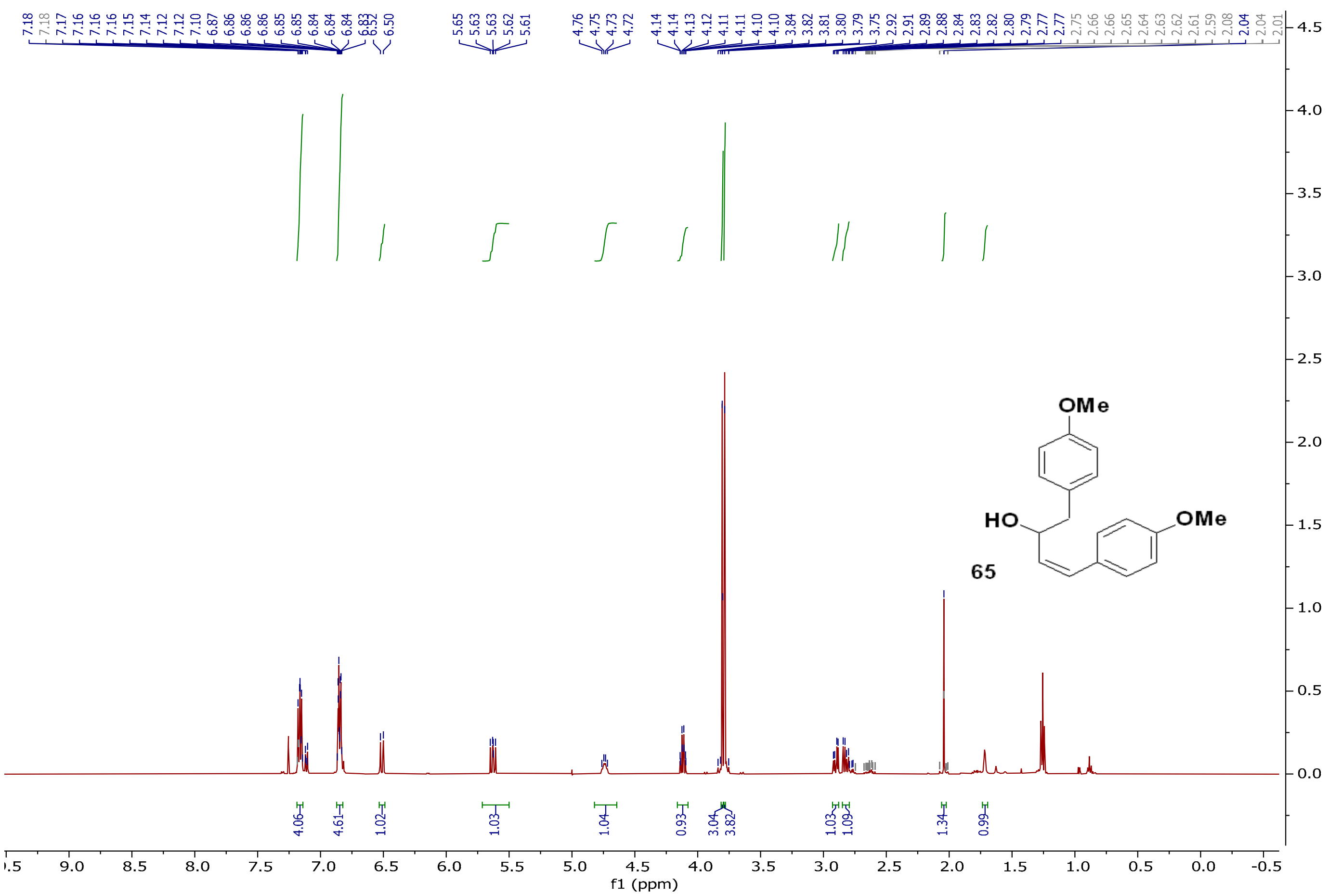

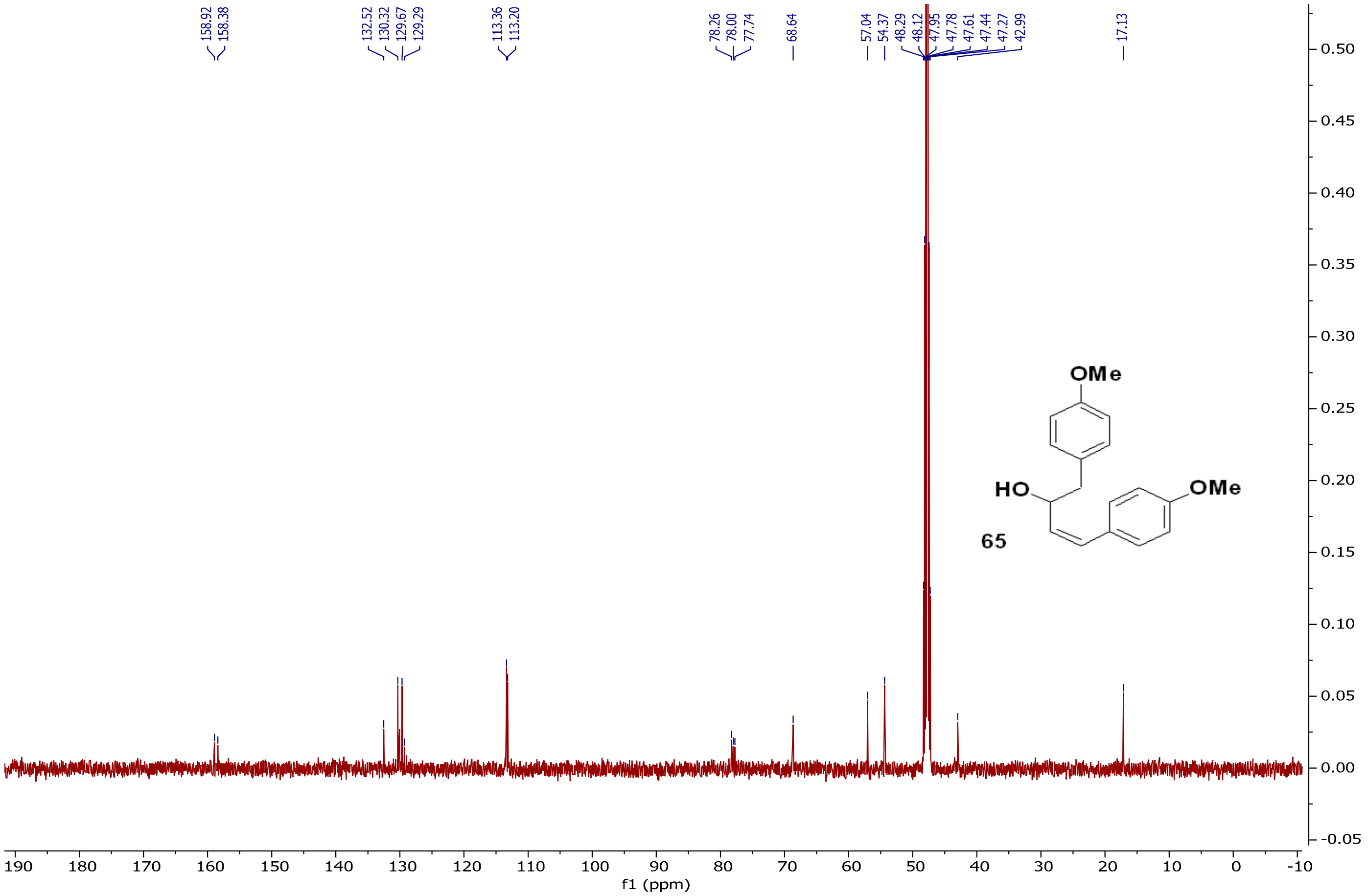

^{1}H and ^{13}C NMR of 66 in $CDCl_3$:

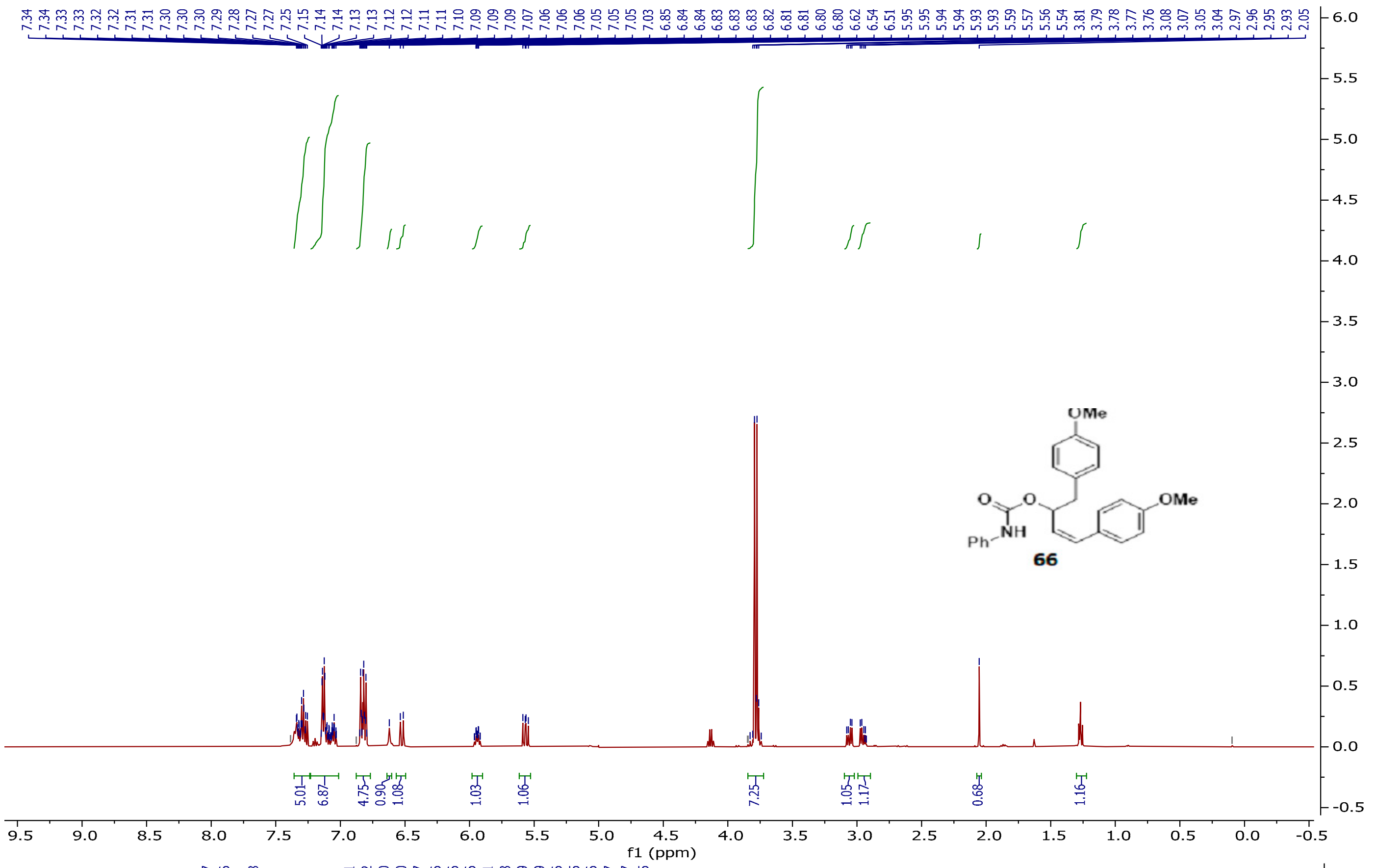

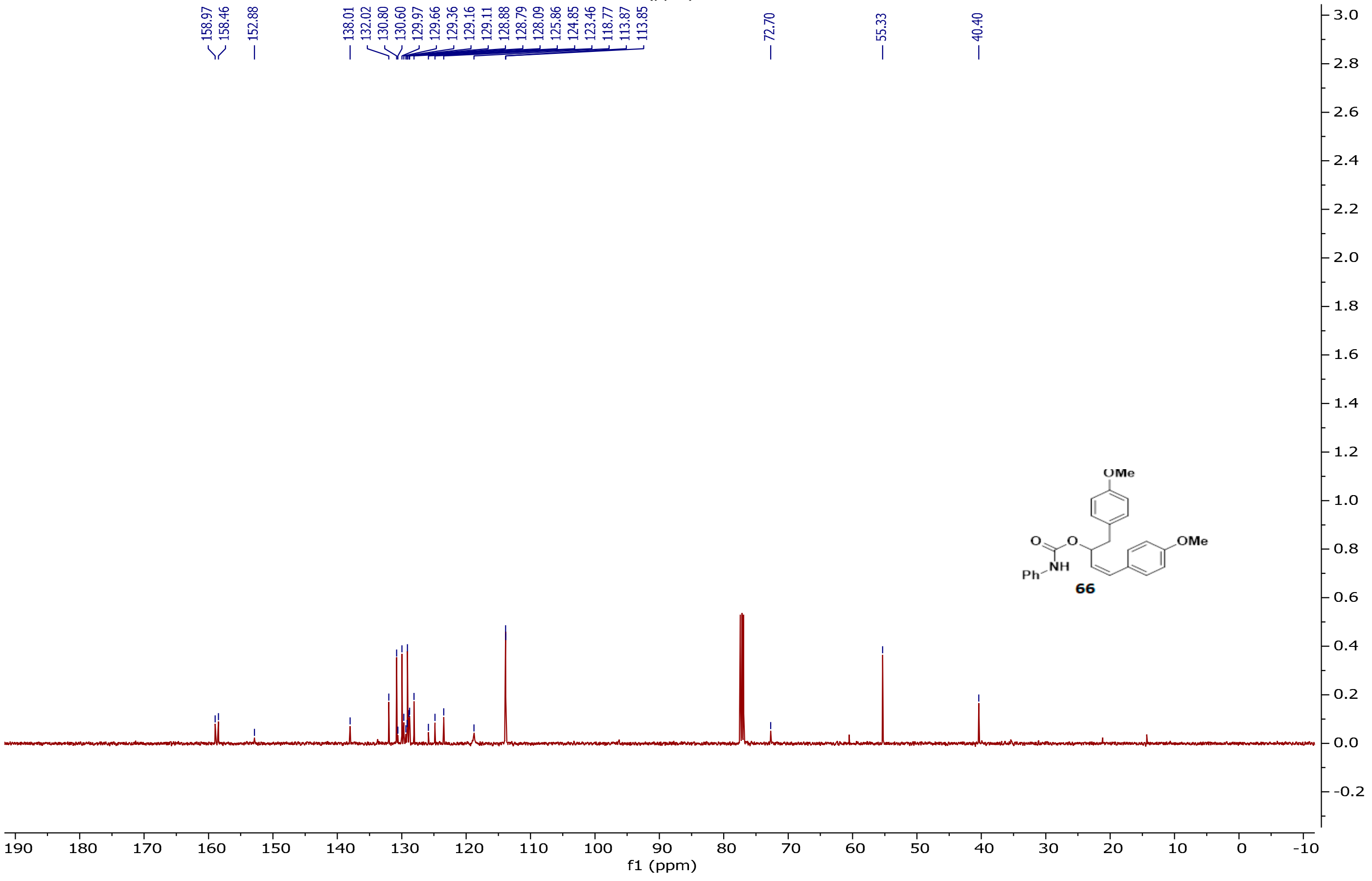

^{1}H and ^{13}C NMR of 69 in $CDCl_3$:

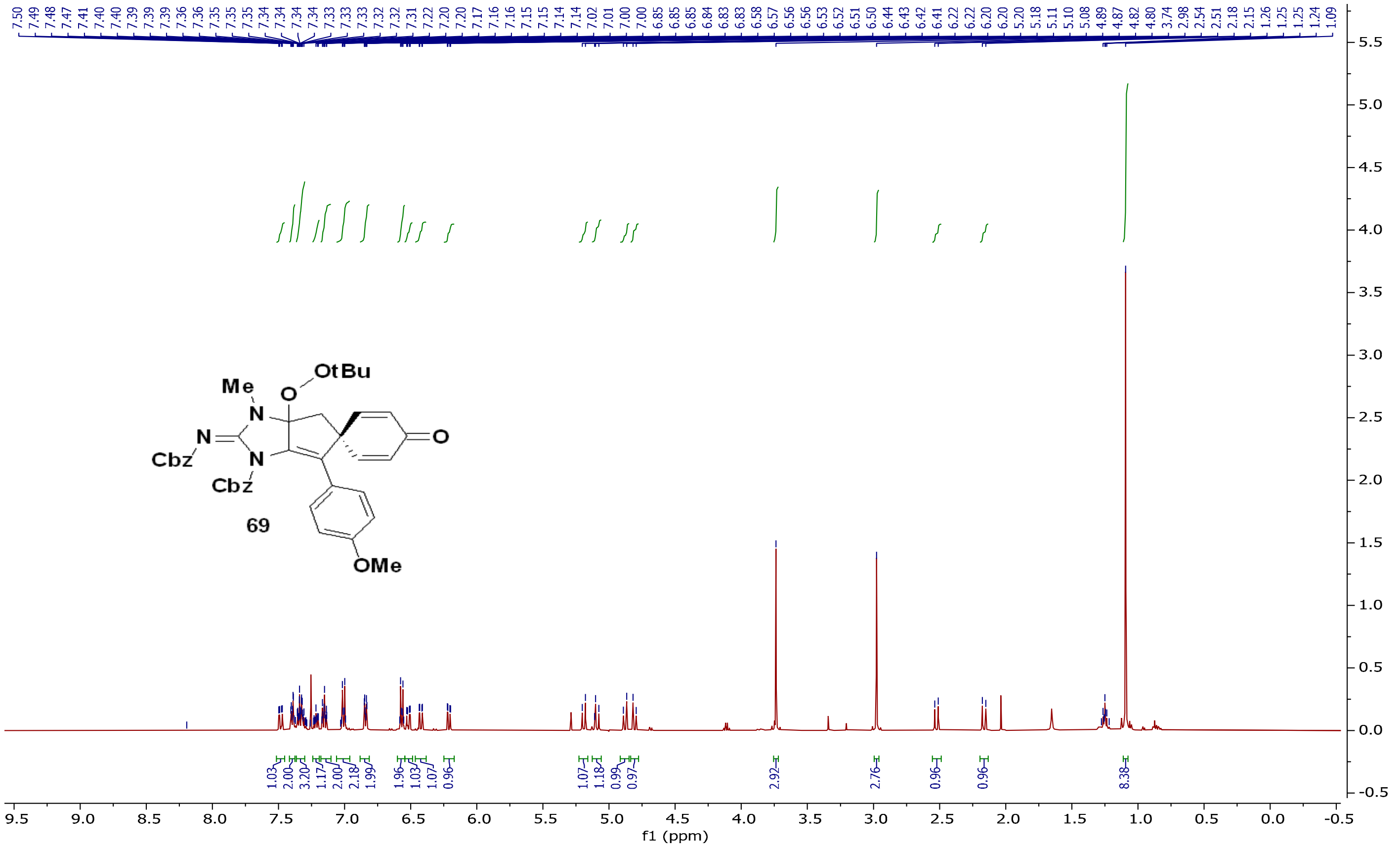

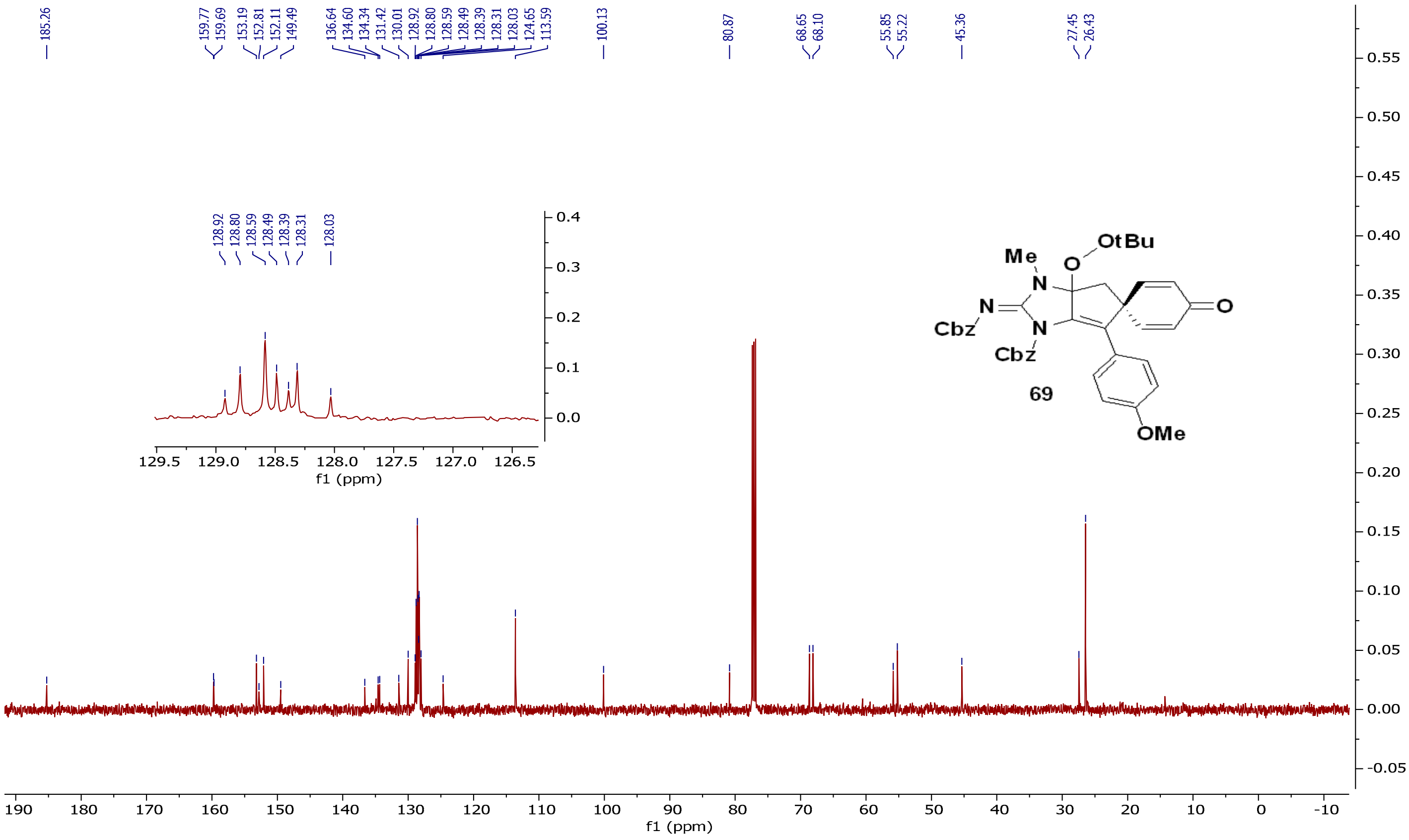

^{1}H of 70 in $CDCl_3$ and ^{13}C NMR in benzene d_6:

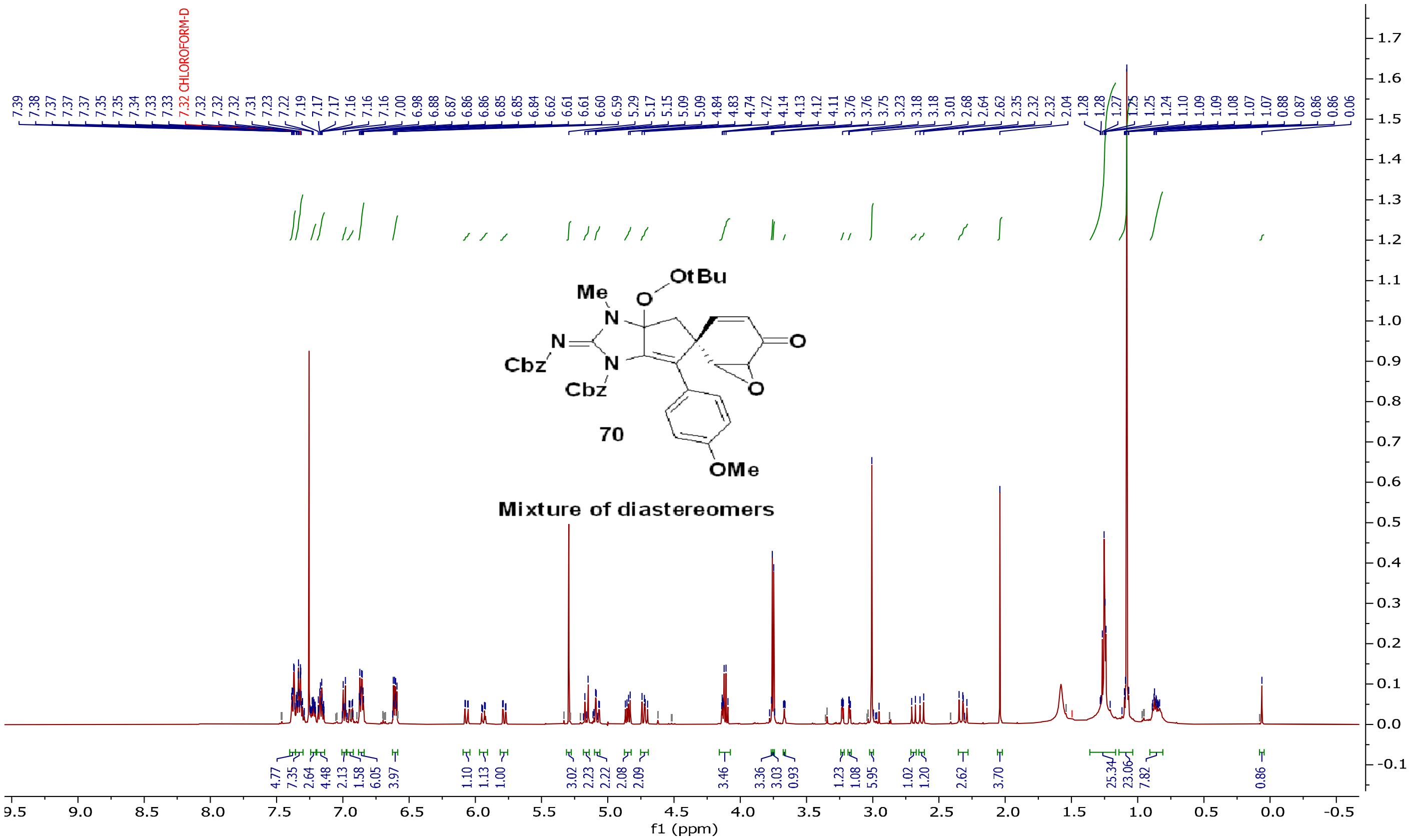

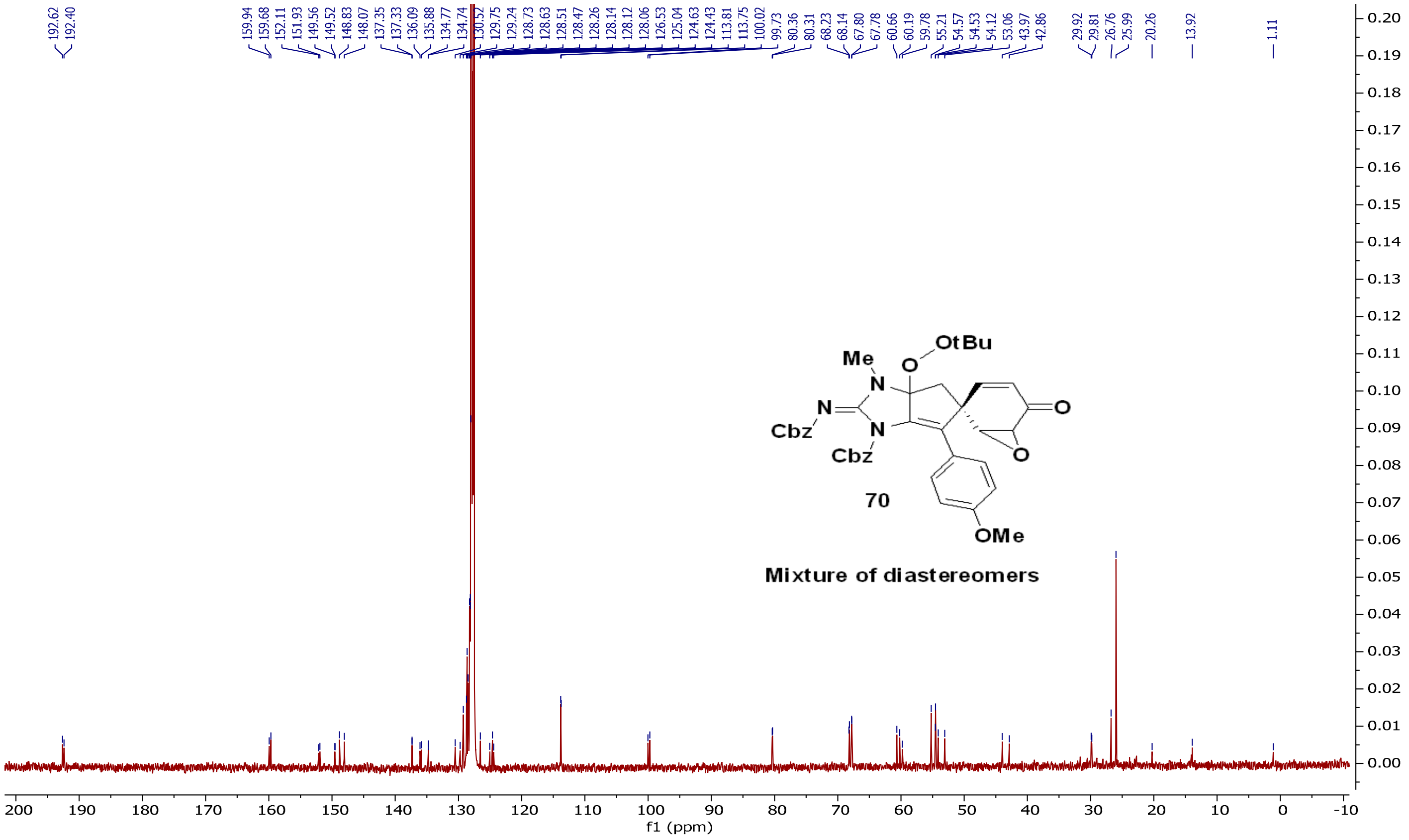

^{1}H and ^{13}C NMR of 71 in $CDCl_3$:

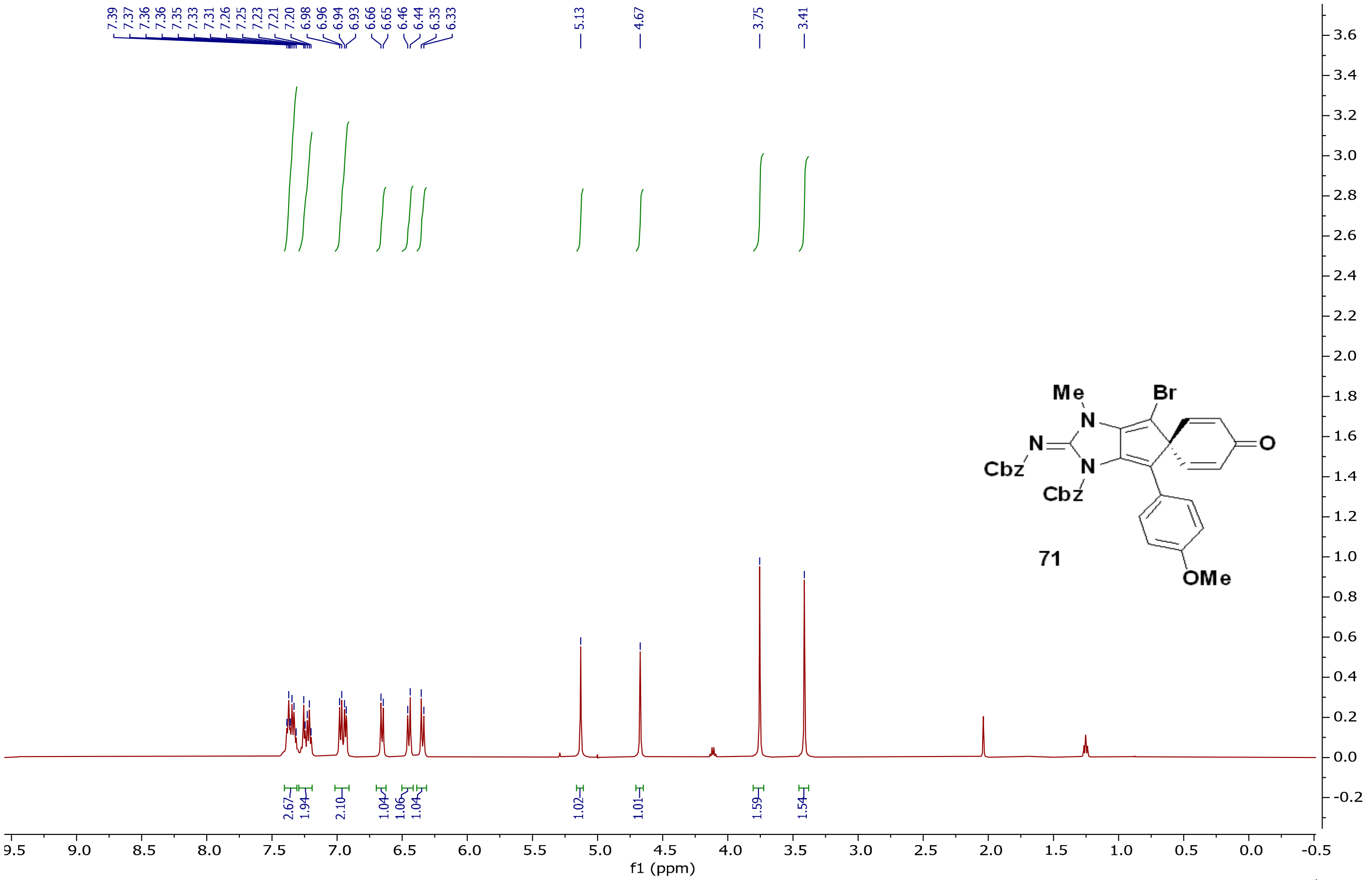

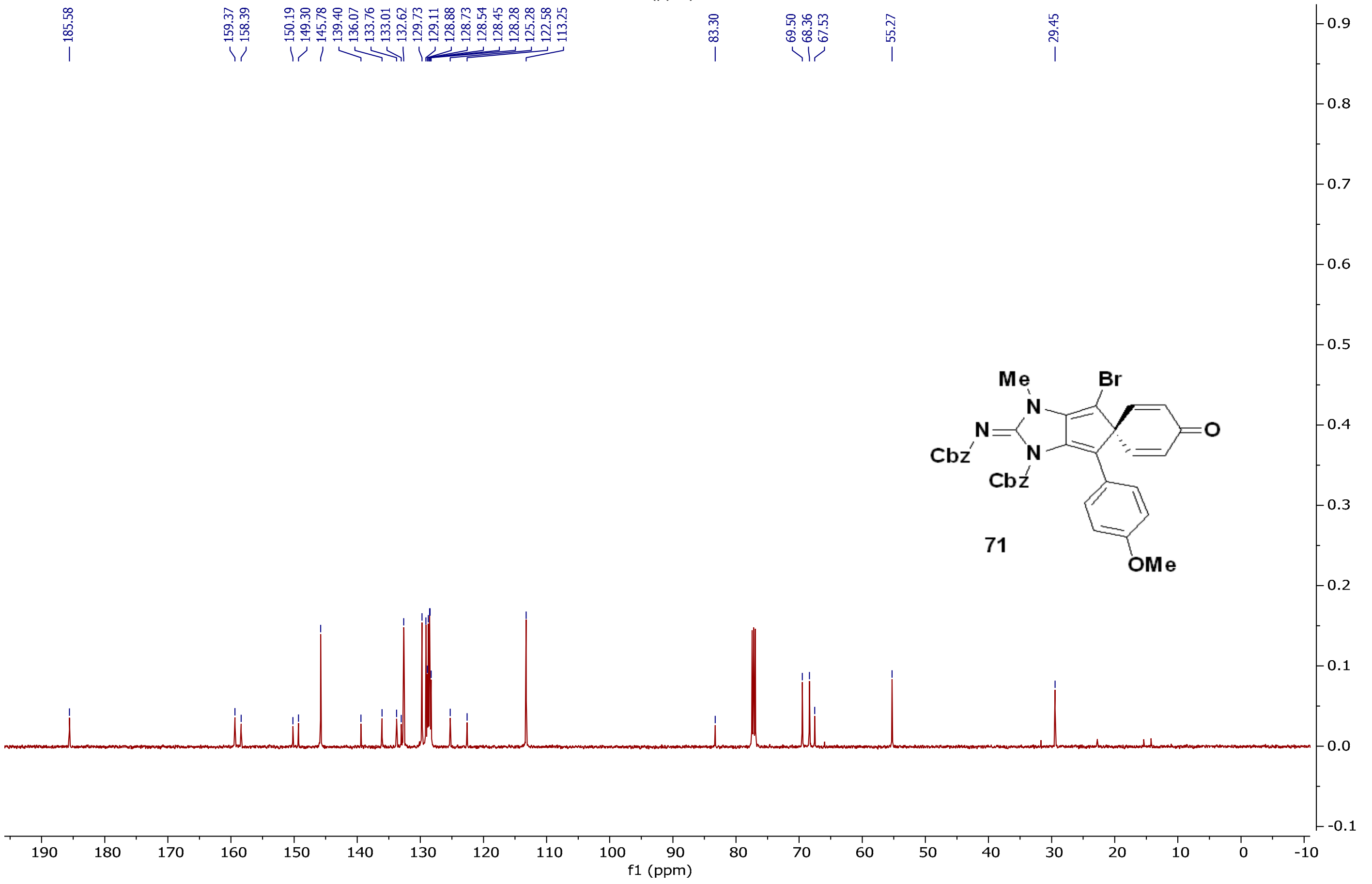

Supplement: Supplementary file 1 [file molecules-30-01143-s001.zip › molecules-3493725-supplementary.pdf]
